# In Vivo Ribosome-Amplified MetaBOlism, RAMBO, Effect Observed by Real Time Pulse Chase, RTPC, NMR Spectroscopy

**DOI:** 10.1021/acs.biochem.5c00086

**Published:** 2025-05-27

**Authors:** Jianchao Yu, Nicholas Sciolino, Leonard Breindel, Qishan Lin, David S. Burz, Alexander Shekhtman

**Affiliations:** † Department of Chemistry, University at Albany, State University of New York, Albany, New York 12222, United States; ‡ RNA Epitranscriptomics & Proteomics Resource, University at Albany, State University of New York, Albany, New York 12222, United States

## Abstract

Quinary interactions between proteins and ribosomes play an important role in regulating biological activity through a phenomenon termed the Ribosome-Amplified MetaBOlism, RAMBO, effect. This effect has been documented in vitro but not in vivo. Real time pulse chase, RTPC, NMR spectroscopy, coupled with isotopic flux analysis in Escherichia coli was used to validate the RAMBO effect in vivo. The ribosomal-targeting antibiotic chloramphenicol was employed to disrupt the quinary structure of pyruvate kinase, the final enzyme in glycolysis. Kinetic flux profiling demonstrated that the in vitro deactivation of the RAMBO effect by chloramphenicol was also observed in vivo, thereby confirming the potential role of ribosomes in regulating glycolysis. The noninvasive modular design of the RTPC-NMR platform allows for high-resolution metabolic monitoring across different cell types, providing broad applicability for studying the real-time metabolic responses to external stimuli in living cells.

## Graphical abstract

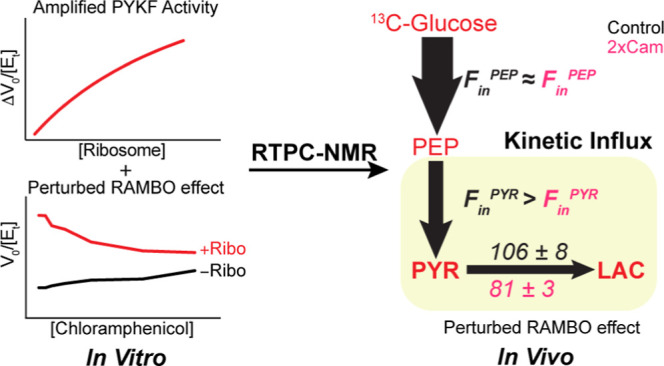

## Introduction

Weak, transient specific protein interactions historically referred to as quinary interactions, are crucial for maintaining protein stability, activity and intracellular homeostasis.^1–4^ Due to their high, micromolar, abundance ribosomes have been identified as key participants in quinary interactions.^5–8^ Ribosome binding to enzymes modulates activity in vitro,^4,6,9^ a process dubbed **R**ibosome-**A**mplified **M**eta**BO**lism or RAMBO.^9^ RAMBO has been proposed to play a regulatory role in metabolism. Recently, an electrostatic mechanism underlying the RAMBO effect was experimentally validated in vitro.^10–12^ The mechanism demonstrated that the ribosomal external electric field, EEF, can interact with the substrate dipole of triosephosphate isomerase, TPI, to generate an interaction energy that enhances ribosome-bound enzyme activity.^10,12–15^ EEF-mediated RAMBO may be ubiquitous across all biological domains given the highly conserved physical properties such as overall structure and charged surface of ribosomes from various sources despite differences in composition and regulatory function.^5,10,16^

To determine whether the RAMBO effect occurs in vivo, attention was focused on the glycolysis pathway, an ancient and extensively studied metabolic pathway for glucose oxidation common to most organisms. In vivo and in vitro proteomics studies have revealed a strong association between cytosolic ribosomes and glycolytic enzymes^17–20^ (Figure 1) and functional assays have confirmed that bacterial ribosomes enhance the activity of the glycolytic enzymes triosephosphate isomerase, TPI, and pyruvate kinase, PYK.^9,10^ Interactions between mitochondrial and chloroplast ribosomes and glycolytic isozymes have also been identified;^21,22^ the similarity between bacterial ribosomes and the idea of common evolutionary origin suggest the potential universality of the RAMBO effect, although broad acceptance and empirical validation remain limited, particularly regarding whether this phenomenon can be observed in vivo.

**Figure 1:**
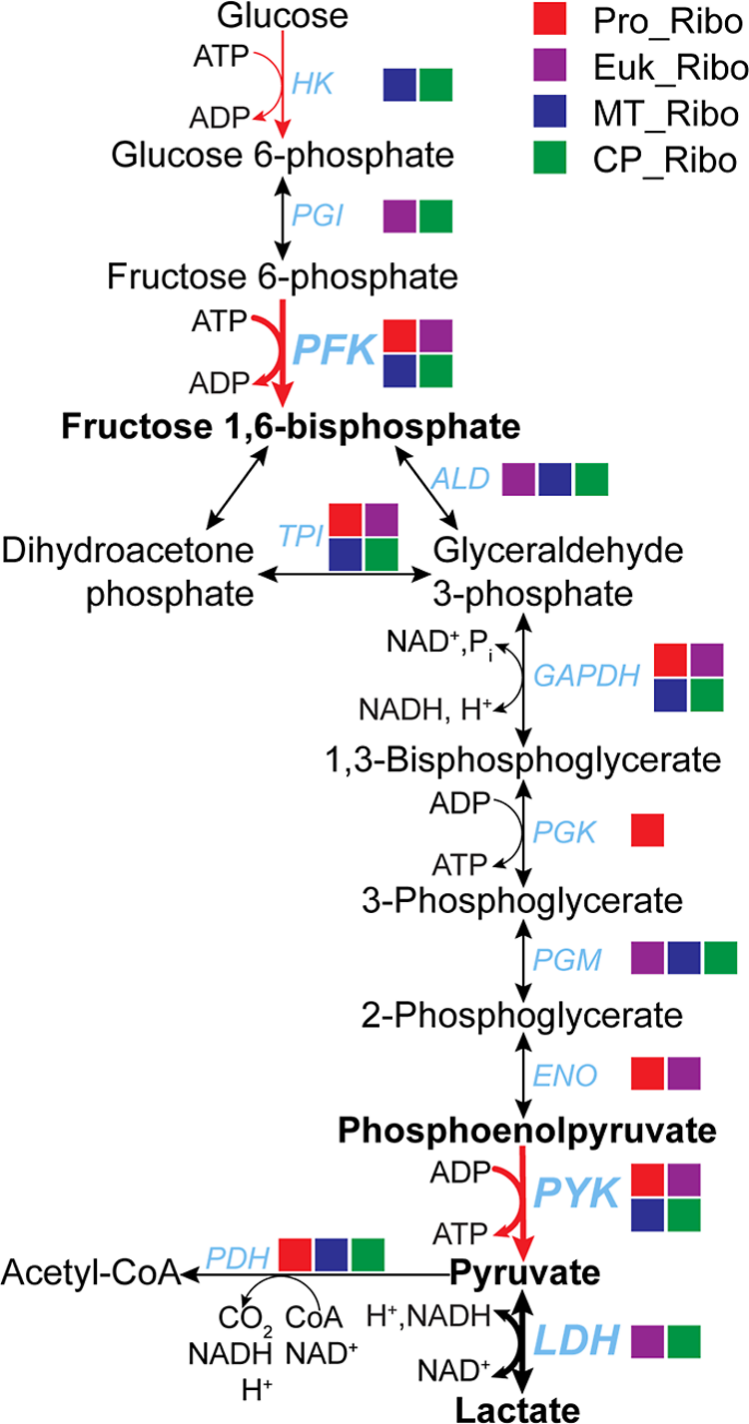
Glycolysis may be regulated by glycolytic enzyme–ribosome interactions. Three major rate-limiting steps of glycolysis are highlighted (red arrows). All enzymes in the pathway have been shown from various sources to be associated with intact ribosomes: prokaryotic ribosomes (Pro_Ribo),^9,10,18,19^ eukaryotic ribosomes (Euk_Ribo),^17^ mitochondrial ribosomes (MT_Ribo)^21^ and chloroplast ribosomes (CP_Ribo).^22^ Abbreviations: HK, hexokinase; PGI, phosphoglucose isomerase; PFK, phosphofructokinase; ALD, aldolase; TPI, triosephosphate isomerase; GAPDH, glyceraldehyde 3-phosphate dehydrogenase; PGK, phosphoglycerate kinase; PGM, phosphoglycerate mutase; ENO, enolase; PYK, pyruvate kinase; LDH, lactate dehydrogenase; PDH, pyruvate dehydrogenase.

Ribosomal-targeting antibiotics that bind to specific sites on the ribosome critical for protein synthesis have been shown to disrupt ribosome-mediated quinary interactions in living cells.^23–25^ Because quinary interactions between ribosomes and metabolic enzymes are typically weak, micromolar affinity, antibiotic binding can alter the local conformation of the ribosome without affecting the activity of a ribosome-bound enzyme.^4–6,9,10^ If the binding disrupts the quinary structure interface of an enzyme that is susceptible to the RAMBO effect, the activity can be altered even if the enzyme remains bound to the ribosome. Monitoring changes in enzyme activity upon exposure to antibiotics can be used to ascertain the RAMBO effect in vivo when it occurs on a time scale that is much faster than the specific effects of the antibiotic. To do this in living cells requires a real-time analysis of living cells, comparing metabolic flux rates in the absence and presence of ribosomal antibiotics. Five ribosomal-targeting antibiotics were tested to determine if the antibiotic–ribosome interactions affect the kinetic activity of the ribosome-bound enzymes.^24,25^ Chloramphenicol was found to be an ideal probe to disrupt ribosome-mediated quinary interactions effectively deactivating the RAMBO effect for pyruvate kinase in vitro rendering it suitable for use in vivo flux analyses of pyruvate metabolism.

RTPC-NMR spectroscopy, which combines bioreactor technology with pulse-chase analysis, has been shown to be effective at monitoring drug-induced metabolism in mammalian cells.^26^ RTPC-NMR monitors the flux of isotopically labeled nutrients through metabolic pathways over time while maintaining cell viability and a high adenylate energy charge, over >24 h, within a 5 mm NMR tube.^27^ By using an ultrasensitive cryoprobe and incorporating carbon selective ^13^C-edited proton NMR pulse programs, temporal resolution of metabolic fluxes has been reduced to 47 s thereby facilitating the capture of intracellular glycolytic fluxes that typically occur within minutes.^28,29^ Previous work demonstrated that kinetic pulse profiles could be observed for lactate, glutamate and alanine.^26^ RTPC-NMR methodology will be used to determine whether the RAMBO effect can be observed in vivo.

Escherichia coli was selected as the model organism for this study due to its extensive use in biochemical genetics and metabolic engineering supported by comprehensive databases such as EcoCyc, which describes the genome, metabolic pathways and regulatory networks of E. coli strain K-12, the Kyoto Encyclopedia of Genes and Genomes, KEGG, a collection of completed genome sequences, Protein Data Bank, PDB, the archive of structural data for biological macromolecules including ribosomes, and numerous relevant publications that aid in determining metabolic phenotypes and quinary binding interfaces.^30–36^ The rapid growth rate of E. coli will rapidly generate the billions of cells required for RTPC-NMR experiments and can be used to produce a high yield of ribosomes and recombinant enzymes for in vitro assays. The common laboratory B strain BL21­(DE3), typically utilized for recombinant protein production, was chosen for this work.^30^ The genome of this strain has been fully sequenced and is similar in size and organization to that of E. coli K-12 MG1655 sharing approximately 99% sequence identity across 92% of the genome.^37^ Note that the absence of flagella in BL21 renders it immobile making it ideal for real time NMR by minimizing cell leakage from encapsulated beads and ensuring stable NMR signals within the sample window.

## Materials and Methods

### Purification of Pyruvate Kinase

Pyruvate kinase I from E. coli, PYK-F (Uniport P0AD61), was purified as previously described with slight modifications.^38,39^ Plasmid pET-28a­(+)-PYK-F, which confers kanamycin resistance and expresses the 50 kDa 470 residue PYK-F monomer with an N-terminal 6 × His-tag, was purchased from Genscript and transformed into E. coli strain BL21 (DE3) for overexpression. Fifty mL of Luria broth, LB, medium containing 50 μg/mL of kanamycin was inoculated with a single colony of transformed cells and incubated at 37 °C and 200 rpm overnight. The overnight culture was diluted to an OD_600_ of ∼0.075 in 1 L of LB medium containing 50 μg/mL of kanamycin and incubated at 37 °C and 200 rpm until an OD_600_ of 0.7–0.8 was reached. Expression of PYK-F was induced with 1 mM isopropyl β-d-thiogalactoside, IPTG, and the cells were incubated at 25 °C and 200 rpm for ∼6 h. The ∼0.6 g cell pellet harvested from 0.2 L of culture was resuspended in 20 mL of ice-cold lysis buffer, 50 mM Tris-HCl, pH 7.5, 10 mM MgCl_2_, 200 mM NaCl, 100 mM KCl, 10% glycerol and 10 mM imidazole containing 10 mM 2-mercaptoethanol and a mini tablet of EDTA-free AEBSF (Roche), as protease inhibitor. The cell suspension was sonicated on ice for 2 min per cycle at 30% amplitude, using a model 250 Digital Sonifier (Branson) with a pulse of 0.3 s on, 1.1 s off. The resulting lysate was centrifuged at 20,000*g* for 20 min at 4 °C, and the supernatant was filtered by using 0.45 μm syringe filters (Pall) before incubation in a 10 mL bed volume of nickel-nitrilotriacetic acid, Ni-NTA, agarose resin equilibrated with lysis buffer at 4 °C for 30 min. The resin suspension was transferred to a batch purification column and rinsed three times with 50 mL of ice-cold lysis buffer containing 30 mM imidazole. The protein was eluted with 2 mL of ice-cold lysis buffer containing 250 mM imidazole. The elution step was repeated 12 times and each fraction was assessed on 10% sodium dodecyl sulfate-polyacrylamide gels, SDS-PAGE. Samples with a purity over 95% were pooled into a 1000 MWCO dialysis bag (SpectraPor, Repligen) and dialyzed twice against 5 L of storage buffer, 10 mM Tris-HCl, pH 7.5, 5 mM KCl, 5 mM MgCl_2_ and 1 mM DTT, to remove imidazole. The purified protein was concentrated by using a 10,000 MWCO centrifugal filter (Amicon) into storage buffer containing 20% glycerol, aliquoted and stored at −80 °C for later experiments. The PYK-F concentrations were determined from the absorbance at 280 nm measured on a NanoDrop 2000 (Thermo Fisher). An extinction coefficient of 29,800 M^–1^ cm^–1^ was calculated using the ProtParam tool of Expasy Server.^40^

### Purification of Ribosomes

Intact 70S ribosomes were isolated from E. coli strain MRE600 at mid log phase and purified using previously published protocols^9,41^ with slight modifications. To minimize the contamination by multienzyme complexes, a high-salt, 20 mM Tris-HCl, pH 7.2, 1 M ammonium chloride, 10 mM magnesium chloride, 0.5 mM EDTA and 6 mM 2-mercaptoethanol, resuspension of the crude ribosome pellet was washed and clarified by centrifugation at 20,000*g* for 30 min at 4 °C. The supernatant was centrifuged at 214,000*g* for 1.25 h at 4 °C in an Optima LE-90K ultracentrifuge using a Ti-90 rotor to isolate 70S ribosomes. The high-salt wash step was repeated at least four times to remove dehydrogenase complex contaminants and residual ATPase from the intact ribosomes. Ribosome purity was assessed by PAGE analysis. The absence of E. coli trigger factor, MW ∼ 50 kDa, and presence of the flexible ribosomal protein S1 of the bacterial ribosome, which migrates as an ∼68 kDa band, confirmed that the high-salt wash effectively removed ribosome-associated factors and did not compromise the integrity of the ribosome prep. The absorbance at 260 nm was used to determine ribosome concentrations using an ε_0.1%_ of 15 mL × mg^–1^ cm^–1^. Only ribosome solutions with a 260:280 ratio of 1.96–2.00 were used.

### Enzyme Activity Assays

All enzyme activity assays were performed in assay buffer, 10 mM sodium phosphate, pH 7.5, 50 mM KCl, 10 mM MgCl_2_, and at 283 K (10 °C) to slow the turnover rate of glycolysis. Phosphofructokinase, PFK-A, from Bacillus stearothermophilus (Uniport entry P00512) and recombinant lactic dehydrogenase, LDH-A, from E. coli (Uniport entry P33232) were purchased from Sigma-Aldrich. Both enzymes, in lyophilized powder form, were reconstituted in a storage buffer containing 20% glycerol, aliquoted and stored at −80 °C for a long-term storage. Concentrations were determined by absorbance at 280 nm measured on a NanoDrop 2000 (Thermo Fisher) using extinction coefficients of 75,640 M^–1^ cm^–1^ for PFK-A and 219,560 M^–1^ cm^–1^ for LDH-A.

PYK-F activity was measured using adenosine 5′-diphosphate, ADP, (Sigma-Aldrich) and phosphoenolpyruvate, PEP, (Bachem) as substrates.^9^ Phosphate buffer was used to minimize background proton signals, and included K^+^ and Mg^2+^ as cofactors for functional PYK-F.^36^ For the ADP saturation curve, the assay was conducted in 0.5 mL of assay buffer containing 10% (v/v) D_2_O (Sigma-Aldrich) and 2 mM PEP in both the absence and presence of 2 μM ribosomes. Reactions were initiated by adding PYK-F to a final concentration of 120 nM. The intensity of the pyruvate methyl proton peak at 2.11 ppm was monitored to determine the rate of product formation. The dependence of PYK-F activity on ribosomes was measured at 2 mM PEP and 4 mM ADP. Five different ribosomal antibiotics were tested to determine if they alter PYK-F activity in the absence and presence of 3 μM ribosomes: streptomycin sulfate, Str, (OmniPur, EMD), Kanamycin monosulfate, Kan, (Gold Biotechnology), Chloramphenicol, Cam, (Sigma-Aldrich), Thiostrepton from Streptomyces azureus, Thr, (Sigma-Aldrich), and Erythromycin, Erm, (Sigma-Aldrich). Water-insoluble antibiotics, Cam, Ths and Erm, were prepared in 100% DMSO-*d*_6_ stock solutions resulting in a final DMSO concentration of ≤14 mM or 0.1% (v/v) in the assays. Controls showed that DMSO did not affect PYK-F activity. The PEP and ADP concentrations remained fixed at 2, and 4 mM, respectively.

PFK-A activity was measured by using adenosine 5′-triphosphate, ATP, (Sigma-Aldrich) and fructose-6-phosphate, F6P, (Sigma-Aldrich), as substrates. For the ATP saturation curve, the assay was performed in 0.5 mL of assay buffer containing 10% D_2_O and 1 mM F6P in the absence of ribosomes. Reactions were initiated by adding PFK-A to a final concentration of 1 nM. The intensity of the fructose-1,6-biphosphate, FBP, proton peak at 3.92 ppm was monitored to determine the rate of production formation. The dependence of PFK-A activity on ribosomes and Cam was measured at 1 mM F6P and 1 mM ATP.

LDH-A activity was measured by using sodium pyruvate, PYR, (Gibco) and β-nicotinamide adenine dinucleotide, reduced disodium salt hydrate, NADH, (Sigma-Aldrich), as substrates. For the pyruvate saturation curve, the assay was performed in 0.5 mL of assay buffer containing 10% D_2_O and 0.5 mM NADH in the absence of ribosomes. Reactions were initiated by adding LDH-A to a final concentration of 0.04 nM. The intensity of the lactate methyl proton peak at 1.06 ppm was monitored to determine the rate of production formation. The dependence of LDH-A activity on ribosomes and Cam was measured at 125 μM PYR and 0.5 mM NADH.

All kinetic experiments were performed at least three times. Data acquisition and analysis followed methods previously described.^9^ Proton peak volumes, exported from MestReNova 14.0.0 (Mestrelab Research), were converted to molar concentrations by normalizing to a 1 mM standard. Initial velocities were calculated from the first 10 data points. Substrate saturation curves were fit to the Michaelis–Menten equation using GraphPad Prism 9

1 $$V_{0}=V_{\max}\left[S\right]/\left(K_{M}+\left[S\right]\right)$$

or to a modified version to include substrate inhibition

$$V_{0}=V_{\max}\left[S\right]/\left[K_{M}+[S]\times \left(1+\left[S\right]/K_{I}\right)\right]$$ 2

where *V*_0_ is the initial reaction rate, *V*_max_ is the maximum velocity, [*S*] is the substrate concentration, *K*_M_ is the Michaelis–Menten constant and *K*_I_ is the dissociation constant for substrate binding. The rate constant *k*_cat_ was calculated as *V*_max_/[*E*_t_], where [*E*_t_] is the total enzyme concentration.

Ribosome titrations of PYK-F activity data were modeled using the “one site-specific binding” model in GraphPad Prism 9

3 $$\Delta V_{0}=V_{\max}\left[R\right]/\left(K_{d}+\left[R\right]\right)$$

where Δ*V*_0_ is the difference in initial velocity relative to the reference velocity, *V*_0_, in the absence of ribosomes, *V*_max_ is the maximum reaction velocity, [*R*] is the ribosome concentration and *K*_d_ is the apparent dissociation constant for the PYK-F-ribosome interaction.

### Cross-Linking and Mass Spectrometry Analysis

Chemical cross-linking experiments were conducted with 25 μM PYK-F and 10 μM ribosomes in 40 μL of cross-linking buffer, 10 mM sodium phosphate, pH 7.5, 100 mM KCl and 10 mM MgCl_2_, with or without 0.125, 0.25, 0.5, and 1 mM of two homobifunctional amine-to-amine cross-linkers: bis-sulfosuccinimidyl suberate, BS_3_, space arm 11.4 Å, and bis-N-succinimidyl-(pentaethylene glycol) ester, BS­(PEG)_5_, space arm 21.7 Å. Reactions were incubated at RT for 30 min and quenched by adding 50 mM Tris-HCl, pH 7.6. Cross-linking reactions were verified by capturing and eluting His-tagged PYK-F from Ni-NTA agarose resins under denaturing conditions (8 M urea) following Qiagen’s standard protocol. Results were visualized by using 10% SDS-PAGE. Two cross-linked PYK-F-ribosome bands were excised from the gels. As controls, cross-linked PYK-F, cross-linked ribosome and a blank at the same molecular weight positions were excised. To control for potential false positives, bands of cross-linked PYK-F, cross-linked RPs and mock-loaded gel at ∼183 and ∼126 kDa were also isolated and processed as previously published.^42^ The excised pieces were minced to ∼1 mm^3^ followed by in-gel tryptic digestion as described by Xue et al.^42^ The tryptic peptides were extracted from gel using 50% acetonitrile plus 5% formic acid three times. The resulting peptide solutions were lyophilized and redissolved in 20 μL of 5% formic acid and 3% acetonitrile for LC–MS/MS analysis. Cross-linking LC–MS/MS measurements were performed using an Orbitrap Velos system (Thermo Scientific) in combination with an Acquity ultra performance liquid chromatography system (Waters) and autosampler, a stream-select module configured for precolumn plus analytical capillary column and an Orbitrap Velos mass spectrometer fitted with an H-ESI probe and utilizing Xcalibur 2.2 software. Twenty microliter samples were trapped and desalted with 0.1% formic acid for 6 min at 40 μL/min on a 15 mm × 500 μm I.D, 5 μm hand packed C18 precolumn cartridge (Grace Davison Discovery Sciences). The peptides were eluted from the precolumn and separated on a 15 cm × 500 μm I.D., 5 μm ACE C18-300 capillary column (Advanced Chromatography Technologies). The C18 column was connected in-line with the mass spectrometer. Peptides were eluted at 20 μL/min with a 40 min gradient of 5–80% acetonitrile in 0.1% formic acid.

Standard mass spectrometry parameters were used: spray voltage 2.3 kV; sheath gas flow 11; heated capillary temperature 275 °C. The system was operated in data dependent acquisition mode. Full scan mass spectra (350–1800 *m*/*z*, and 30,000 resolution) were detected in the orbitrap analyzer after accumulation of one million ions. For every full scan, MS were collected during a 3 s cycle time. Ions were isolated (2.3 *m*/*z* isolation width) for a maximum of 250 ms or 75% of automatic gain control target with the automatic maximum injection time setting enabled for parallelization. Ions were fragmented by high energy collision dissociation with 32% normalized collision energy at a resolution of 45,000. Charge states <2 and >4 were excluded, and precursors were excluded from selection for 30 s if fragmented *n* = 2 times within a 20 s window.

### Analysis of Cross-Linked Peptides

All tandem spectrum data was processed using Mascot 2.8 distiller (Matrix Science). The tandem spectrum peak list was searched using pLink 2 software^43^ against a targeted protein list with modified settings: 500 Da < peptide mass < 6000 Da, 5 < peptide length < 60, precursor tolerance of ±5 ppm, fragment tolerance of ±10 ppm and false discovery rate of <5%. For the cross-linker BS­(PEG)_5_ a monoisotopic linker mass shift of 302.136555 Da and a mono mass shift of 320.14712 Da were set. Cysteine carbamidomethylation and methionine oxidation were selected as fixed and variable modifications, respectively. To maximize the outputs of potential cross-linked peptides, a parameter including either trypsin, allowing up to five missed cleavages, or nonspecific was used for tandem MS spectrum assignment. Only intermolecular cross-links with a precursor mass error within ±4 ppm were considered for the next step after searching. Fragmentation spectra were manually checked by pLabel,^44^ a software tool of pFind. The solvent accessibility of cross-linked residues from TPI and ribosomal proteins, RPs, was analyzed by using GETAREA with a water probe radius of 1.4 Å.^45^ Manual inspection was performed by using rigid structural models in UCSF ChimeraX version 1.3.^46^

### Cell Growth

Plasmid pET-21a­(+), which confers ampicillin resistance, was transformed into E. coli strain BL21­(DE3). Forty milliliters of LB medium containing 100 μg/mL of carbenicillin was inoculated with a single colony of transformed cells and incubated at 37 °C and 200 rpm overnight. The overnight culture was diluted into to 500 mL of LB medium containing 100 μg/mL of carbenicillin to an OD_600_ of ∼0.075 and incubated at 37 °C and 200 rpm until an OD_600_ of 0.56 ± 0.014 was achieved. The culture was harvested by centrifugation at 2000*g* for 25 min at 4 °C, and the resulting pellet was washed twice with hybrid growth medium salts buffer, HGM, 50 mM HEPES, pH 7.5, 118 mM NaCl, 4.7 mM KCl, 1.0 mM MgSO_4_, 2.5 mM CaCl_2_, 3 mM NaH_2_PO_4_, 100 μg/mL carbenicillin and 12% (v/v) D_2_O. The growth medium and salts buffer were sterilized using 0.2 mm Nalgene Rapid-Flow bottle top filters with poly­(ether sulfone) membranes or syringe filters (Thermo Scientific). The final cell resuspension was centrifuged at 2000*g* for 7 min at room temperature, RT, and prepared for casting in alginate.

### Cell Casting

Cell casting was performed as previously described with slight modifications.^27^ Ten mL of HGM was added to 0.2 g of alginate powder (Sigma-Aldrich) and stirred gently overnight at RT to dissolve the alginate. Approximately 2 × 10^11^ pelleted E. coli strain BL21­(DE3) cells (∼500 μL) were mixed 1:1 (v/v) with the alginate solution. The mixture was transferred to a 3 mL BD syringe fitted with a Luer–Lok tip connected to 40 mm of 0.79 mm I.D. Tygon tubing with a blunt 21-gauge needle. The needle was oriented at 45° with respect to the surface of 40 mL of 150 mM CaCl_2_ in a 50 mL beaker. The cell-alginate suspension was injected into an atomizer at 300 μL/min using an NE-300 syringe pump (New Era). The atomizer consisted of a vertically oriented 5 mL pipet with an airflow of 5 L/min. As the suspension drops contacted the CaCl_2_ solution, the alginate polymerized into beads that encapsulated the cells.^47^

The CaCl_2_ solution was decanted and replaced with 10 mL of HGM supplemented with 11.1 mM (2 g/L) glucose and 18.7 mM (1 g/L) NH_4_Cl. The bead suspension was gently poured over presterilized lab sieves with 304 stainless steel wire cloth (mesh diameter 0.5 mm, LABALPHA) to remove all tiny beads (Figure S1A). The retained beads were rinsed twice with fresh HGM. After rough size selection by lab sieves, beads larger than 0.5 mm in diameter were transferred to a presterilized 100 × 20 mm glass Petri culture dish (Pyrex) with fresh HGM. Uniformly shaped cell beads were manually picked using 200 mL large-orifice pipet tips (Fisherbrand) and transferred to a standard 5 mm screw-cap NMR tube. Overall, it takes 0.5–1 h to package cell beads and for the cells to adapt to HGM at RT. This procedure produces a uniform dispersion of cell beads 0.91 ± 0.017 mm in diameter that can resist the hydrodynamic drag resulting from a 100–200 mL/min flow rate and ensure homogeneous nutrient uptake to yield consistent metabolic phenotypes (Figure S1B).^27,47^ Magnified images of cell beads were captured using an Evos FL cell imaging system (Thermo Fisher). The captured images were processed and analyzed using ImageJ software.

### Bioreactor Setup

The NMR bioreactor was used as previously described with slight modifications.^26,27^ The perfusion bioreactor comprised three principal modules: an inlet from the medium reservoir with an injectable loop, a drip irrigation stem with a microporous diffuser tip and an outlet to waste driven by a peristaltic pump. The reservoir was filled with fresh HGM supplemented with glucose and NH_4_Cl at room temperature to continuously deliver fresh nutrients to the cells.^27^ An injector (Rheodyne) was connected to the inlet line 120 cm from the reservoir and fitted with a 15 mL injection loop constructed out of 1.19 mm I.D. polyethylene tubing (Clay Adams).

Approximately 600 μL of cell beads were loaded into the NMR tube to ∼5 cm from the tube bottom to cover the entire detection window while leaving about 10% free space for the inlet stem. To maximize signal detection, cell beads were immobilized within the NMR detection window by inserting a 4.5 mm diameter, 100 μm, 1.2 mm thick hydrophilic frit to into the NMR tube above the beads. A hole was drilled in the center of the frit to accommodate the drip irrigation stem. The microporous irrigation stem was inserted into the bottom of the packaged cell beads to ensure an even distribution of fresh medium across the entire sample. Medium was flowed through the packaged NMR tube for 0.5 h at RT to test for leaks and to allow the cells to adapt to conditions inside the NMR tube.

Metabolic products and unconsumed nutrients were removed by an outlet tubing at the top of the bioreactor at a flow rate of 100–200 μL/min controlled by a PeriPumpONE peristaltic pump (New Era) and directed to a waste reservoir. To perform an RTPC experiment the pump was programmed in three stages: (i) 100 μL/min for 20 min, (ii) 200 μL/min for 20 min, and (iii) 100 μL/min until turning off the pump. Flow-through was collected in ∼1.8 mL fractions (20 min each) over the course of the experiment starting at the initiation of the ^13^C pulse to assess cell leakage, extracellular metabolic products and the concentration of Cam. To slow the rapid turnover of glycolysis in E. coli, which typically occurs within minutes under optimal conditions, the experimental temperature was adjusted to 283 K to capture enough kinetic data points for accurate flux modeling.^28,29^

To measure metabolic fluxes the injector loop was loaded with 10.8 mM [U–^13^C] glucose (2 g/L) and 18.7 mM NH_4_Cl (1 g/L) in fresh HGM in the absence and presence of 35 μg/mL Cam (1 × Cam) and 70 μg/mL Cam (2 × Cam). By switching the valve to injection mode, the ^13^C-labeled medium pulse is uninterruptedly flowed into the bioreactor.

### NMR Spectroscopy

All NMR experiments were recorded at 283 K using a 600 MHz Bruker AVANCE III NMR spectrometer equipped with a QCI-P cryoprobe. To measure kinetics in vitro, pseudotwo dimensional ^1^H NMR experiments were recorded with 16 transients and a 2.13 s interval between transients, 1 s acquisition time and a 1 s relaxation delay. These experiments incorporated td1 = 50 data points as the second dimension. The spectral width in the proton dimension was 20 ppm.

Two dimensional, 2D, heteronuclear single quantum coherence, ^1^H–^13^C HSQC, spectra were recorded to monitor metabolite fluxes and to assign ^13^C-labeled metabolites. 2048 and 512 points were acquired in the ^1^H and ^13^C dimensions, respectively, with 32 transients. The spectral widths in the proton and carbon dimensions were 16 and 80 ppm, respectively. Peaks were assigned by using the metabolomics database in ECMDB^48^ and BMRB^49^ and were quantitated by comparing to 1 mM standards prepared in HGM from a metabolite library, which includes 28 metabolites from glycolysis and the TCA cycle, 21 essential amino acids and 6 nucleotides. The genome and pathway databases of EcoCyc^31^ and KEGG^32^ were used for metabolic pathway and expressed enzyme verification in E. coli strain BL21 (DE3). All spectra were processed with Topspin 4.2.0 (Bruker) and analyzed using CARA software.

To assess the energy state of the cells, proton-decoupled ^31^P spectra were collected at 3.5 h intervals for each experiment prior to administering the ^13^C-glucose pulse and after the ^12^C chase. ^31^P spectra were recorded with 6144 scans and a 1 s recycle delay, centered at −10 ppm, corresponding to 242.935 MHz, and a spectral width of 30 ppm. The γ-NTP peak intensity at −5.97 ppm was integrated from −5.50 to −6.18 ppm. Two peaks of phosphorylated compounds, including, glucose-6-phosphate, G6P, at 4.05 ppm and FBP at 4.37 ppm, were integrated from 4.14 to 3.83 ppm for G6P and from 4.48 to 4.26 ppm for FBP to track the changes in early glycolysis upon 2 × Cam treatment. Moreover, the peak intensity at −11.48 ppm from the cellular redox couples of NAD­(H) and NADP­(H), including NAD^+^/NADH, and NADP^+^/NADPH, respectively, was integrated from −11.45 to −11.54 ppm and used to assess changes in the overall redox pool upon 2 × Cam treatment. All assigned peaks were determined by using 1 mM standards of 17 phosphorylated compounds in HGM.

To improve the temporal resolution of metabolic rates in glycolysis, a 1D ^13^C-edited version of ^1^H–^13^C HSQC was employed to acquire data at 47 s intervals; 2048 points and 1 point were acquired in the proton and carbon dimensions, respectively, with 32 transients. Prior to the ^13^C pulse, a background reading of the 1D ^1^H–^13^C HSQC spectrum was recorded to quantitate the ^13^C natural abundance of metabolites. Following the ^13^C pulse, a total of 500 spectra were collected consecutively over ∼6.5 h to monitor the ^13^C pulse and ^12^C chase phases. All RTPC-NMR 1D experiments with and without Cam treatments were conducted at least in triplicate.

### Kinetic Flux Profiling Analysis

All 1D ^1^H–^13^C HSQC spectra were processed and analyzed using MestReNova 14.0.0 (Mestrelab Research). Peak amplitudes were exported from the Peaks Graph function of MestReNova and further analyzed using Excel (Microsoft). Relative peak amplitudes, A, were calculated as

4 $$A=\left(A_{N}/A_{HEPES\text{\_}N}\right)-\left(A_{0}/A_{HEPES\text{\_}0}\right)$$

where *N* = 1–500 represents the 1D experiment number after initializing the ^13^C pulse. *A*_*N*_/*A*_HEPES_N_ is the normalized peak amplitude before background subtraction, and *A*_0_/*A*_HEPES_0_ represents the background signal at *T*_0_. *A*_HEPES_ is the peak amplitude of HEPES at 2.91 ppm. The normalized amplitudes, *A*, of the metabolite peaks were converted to molarity by plotting against the natural ^13^C abundance of in vitro samples. The resulting calibration curves (Figure S2) were fit to a linear equation

5 $$c^{\prime \prime }=kA+b$$

where *c*″ is the prescaled molarity in mM, *k* is the slope of the line, and *b* is the *y*-intercept. To compare amplitudes from each experiment, the molarities were scaled to the γ-NTP levels observed in the first experimental trial

6 $$c^{\prime }=c^{\prime \prime }\left(I_{\gamma NTP\text{\_}1}/I_{\gamma NTP\text{\_}n}\right)$$

where *c*′ is the scaled molarity in mM, *I*_γNTP_1_ is the integrated intensity of γ-NTP in the initial experiment, and *I*_γNTP_*n*_ is the integrated intensity of γ-NTP in the ensuing experiments. Finally, the scaled molarities were divided by the intensity of 1 μM γ-ATP for per-cell level estimation

7 $$c=\left(c^{\prime }/I_{\gamma ATP}\right)\times 1000$$

where *c* is the molarity in μM per μM ATP.

The signal-to-noise ratio of the formate signal was low, so formate data were exported to OriginPro 2022 for data smoothing. The adjacent-averaging method was used for smoothing with the option of weighted average and 50 as the points of window, with symmetry set for the boundary condition. The change in the relative concentration, c, of each metabolite during the ^13^C pulse was analyzed using GraphPad Prism 9. The leading edge of each metabolite ^13^C pulse (≤50 min) was modeled as a one-phase association followed by a plateau

8 $$c=c_{0},\;\text{for}\;T<T_{0}$$

9 c=c0+(Plateau−c0)×{1−exp[−K(T−T0)]},forT≥T0

where *T*_0_ is the time at which the ^13^C pulse was initiated, *c*_0_ is the average relative concentration up to time *T*_0_, Plateau is the maximum relative concentration and *K* is the rate constant of the association. All outputs must pass the 95% confidence level under the asymmetrical option of GraphPad. The rate of production of each metabolite, *F*_in_, was computed as

10 $$F_{in}=Plateau\times K$$

### Statistical Analysis

The independent sample *t*-test assumed with equal variances was used to assess the statistical significance of data obtained in this study and two-tailed *p* values were computed by *t* scores by using the online *p* value calculator of GraphPad (https://www.graphpad.com/quickcalcs/pValue1/). Cohen’s *d*, *d* values, measures were calculated to determine the magnitude of effect. Commonly, *d* values of 0.2, 0.5, and 0.8 indicate small, medium, and large differences in the mean experimental values, respectively.^50^ All values with error stated in the text are mean ± the standard error of mean (SEM).

## Results

### Ribosomes Alter PYK-F Activity

Pyruvate kinase is the critical step in glycolysis that irreversibly produces ATP, converting PEP to pyruvate, PYR. E. coli has two types of pyruvate kinase: PYK-F, encoded by *PYK-F*, and PYK-A, encoded by *PYK-A*. PYK-F is allosterically activated by fructose 1,6-biphosphate, FBP, and inhibited by ATP and succinyl-CoA, while PYK-A is allosterically activated by AMP and monophosphorylated sugars.^36,51–53^ Both enzymes function as homotetramers and are regulated independently. PYK-A contributes ∼7% of pyruvate kinase activity under aerobic conditions and ∼30% under anaerobic conditions.^51^ Due to the predominant physiological role of PYK-F this work focused on the effect of ribosomes and ribosome-targeting antibiotics on the steady-state kinetics of PYK-F.

Intact ribosomes were purified from E. coli strain MRE600 to match the physiological source of PYK-F. Traditional methods for ribosome purification result in contamination with multienzyme complexes such as pyruvate dehydrogenase, PDH, and α-ketoglutarate dehydrogenase, α-KGDH, which have molecular weights comparable to the 70S ribosome.^54,55^ Solutions containing these complexes are yellow due to flavin cofactors.^56^ Additionally, ribosome-associated ATPase and translational factors attach to intact ribosomes during ultracentrifugation, potentially affecting enzyme activity assays as both ATP and NAD^+^/NADH are involved in key catabolic steps.^41^ A high-salt wash was used and repeated at least four times to remove these contaminants. Further verification of ribosome purity was accomplished by monitoring the decay in ATPase and redox activity accompanying each wash step by using pseudo-2D proton NMR (Figure S3).

Purified N-terminal His-tagged E. coli PYK-F was prepared (Figure S4) and PYK-F activity was characterized using a direct in vitro NMR kinetic assay previously validated for pyruvate kinase, PYK, from Bacillus stearothermophilus, *Bs* PYK.^9^ The assay monitors the proton peak intensity of substrates and/or products over time and is applicable to most metabolic enzymes.^9,10^ All assays were performed at 10 °C to slow the catalytic reaction and to allow a direct comparison with in-cell results also run at 10 °C to slow the characteristic time of glycolysis to 10–20 min, for glycolytic flux analysis. Note that this temperature setting allows decoupling of the temporal RAMBO effect from long-term transcription and translation by slowing the E. coli doubling time to days.^30,57–61^ PYK-F activity increased with increasing amounts of intact ribosomes up to 8 μM, approaching the intracellular ribosome concentration in E. coli during exponential growth (Figure 2A).^7^ The effect did not reach saturation because higher ribosome concentrations resulted in more than 10% loss of ADP during the reaction dead time leading to inaccurate initial velocity rates. An apparent affinity of 10 ± 3.7 μM (*R* ^2^ = 0.94) was resolved for the binding of intact ribosomes to PYK-F suggesting that the interaction is specific.

**Figure 2:**
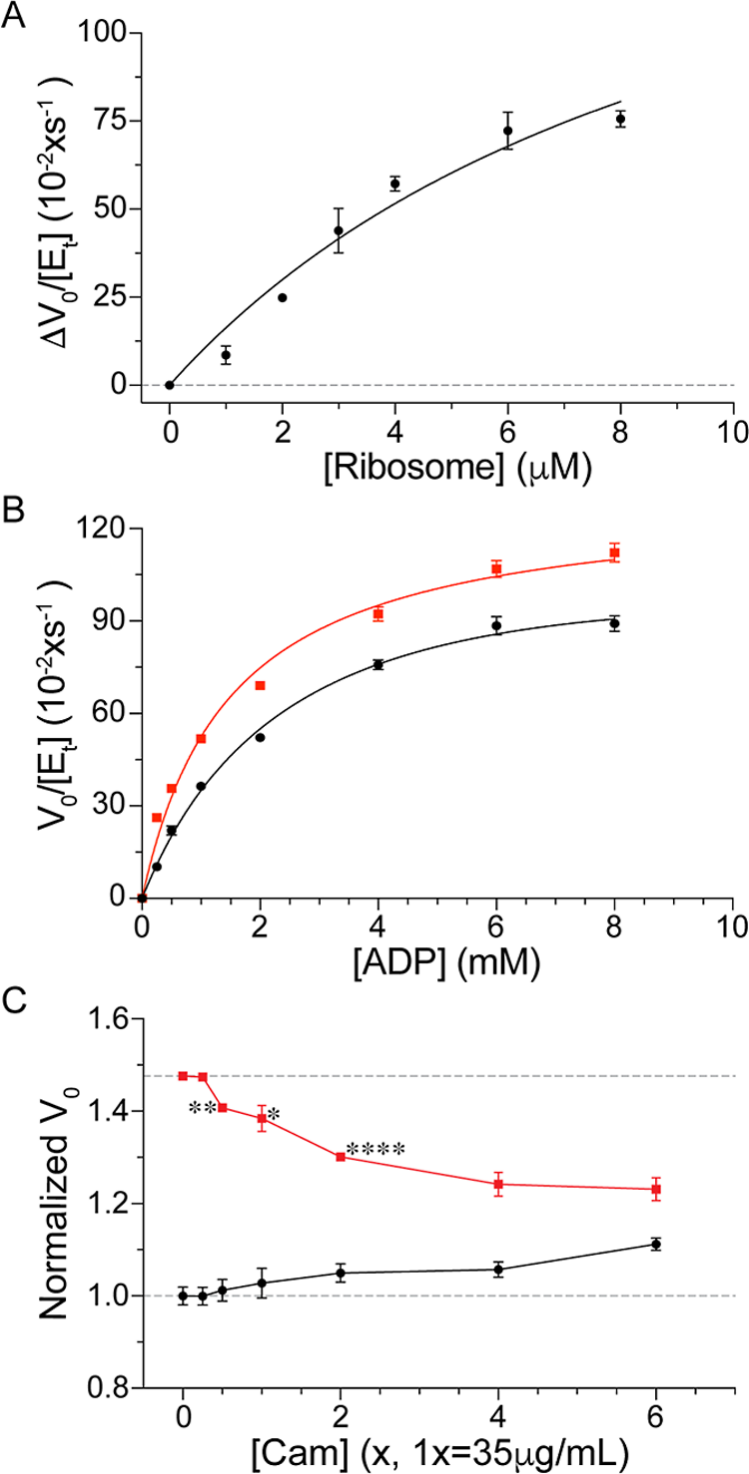
Ribosomes affect PYK-F kinetic activity. (A) Ribosome binding to PYK-F increases activity. The change in activity, Δ*V*_0_, is relative to PYK-F activity in the absence of ribosomes. [*E*_t_] is the total concentration of PYK-F. The concentrations of ADP and PEP were 4 and 2 mM, respectively. Data were fit to a one-site binding model (eq 3), (B) saturation kinetic curves in the absence (black) and presence (red) of 2 μM ribosomes. The concentration of PEP was 2 mM. Data were fit to the Michaelis–Menten eq (eq 1). (C) Effect of Cam on free (black) and ribosome-bound (red) PYK-F activity. The concentrations of ADP and PEP were 4 and 2 mM, respectively. *V*_0_ was normalized to the initial velocity of PYK-F in the absence of 3 μM intact ribosomes and Cam. The statistical significance of the changes in the normalized *V*_0_ for ribosome-bound PYK-F is shown for 0.5×, 1× and 2× Cam concentrations (****, *p* < 0.0001 and *d* > 2.0; **, *p* < 0.01 and *d* > 2.0; *, *p* < 0.05 and *d* > 2.0). Error bars show means ± standard errors of the mean (SEM) from three independent trials.

In the absence of ribosomes a *K*_M_ of 2.3 ± 0.18 mM was resolved for PYK-F, in agreement with the previously determined value of 1.6 mM for *Bs* PYK, however *k*_cat_ was ∼500× smaller (Table 1). In the presence of 2 μM ribosomes, *K*_M_ decreased by 35% (*p* < 0.001, *d* > 1), comparable to the decrease in *K*_M_ previously observed and *k*_cat_ increased by ∼10% (*p* < 0.05 and *d* > 0.5) somewhat less than previously observed (Figure 2B). The effect of ribosomes on activity is statistically significant, as determined by independent samples *t*-test and Cohen’s *d* measures.^62^ The substantial decrease in *k*_cat_ was expected due to the lower assay temperature, 10 °C vs 30 °C, resulting in slower substrate diffusion and nonspecific binding by inorganic phosphate at the active site. Overall, the trends observed are consistent with previously observed ribosome-mediated *Bs* PYK activity (Table 1).^9^

**Table 1:** Steady-State Kinetic Parameters Resolved for PYK-F^g^

	no ribosomes			ribosomes		
substrate	*K*_M_ (mM)	*k*_cat_ (10^–2^ × s^–1^)	*R* ^2^	*K*_M_ (mM)	*k*_cat_ (10^–2^ × s^–1^)	*R* ^2^
ADP^a^	2.3 ± 0.18	118 ± 3.3	0.99	1.5 ± 0.13^b^	130 ± 3.6^c^	0.99
ADP^d^	1.6 ± 0.17	63,900 ± 500	0.99	1.0 ± 0.12^e^	79,500 ± 1100^f^	0.98

a This work direct NMR kinetic assay, 10 °C, pH 7.5, 2 mM PEP, 120 nM E. coli PYK-F.

b *K*_M_ decreased significantly in the presence of ribosomes (***, *p* < 0.001 and *d* > 1.0).

c *k*_cat_ increased significantly in the presence of ribosomes (*, *p* < 0.05 and *d* > 0.5).

d Previous work^9^ direct NMR kinetic assay, 30 °C, pH 7.2, 2 mM PEP, 0.3 nM *Bs* PYK and 0.5 μM ribosomes.

e *K*_M_ decreased significantly in the presence of ribosomes (**, *p* < 0.01 and *d* > 1.0).

f *k*_cat_ increased significantly in the presence of ribosomes (***, *p* < 0.001 and *d* > 1.0).

g Note: Values are means ± SEM.

The PYK-F tetramer–dimer equilibrium has a dissociation constant, *K*_d_, estimated to be in the picomolar range, indicating very tight binding.^63^ The concentration of PYK-F used for all measurements was 120 nM, ≥4 orders of magnitude above the estimated *K*_d_. Thus, tetrameric PYK-F is the sole species interacting with the ribosome and ribosome-mediated alterations of the dimer–tetramer equilibrium are unlikely to contribute to changes in PYK-F activity. In addition, the intact ribosome particles used in functional assays were free of ribosome-associated factors (Figure S3). Therefore, the enhancement in the catalytic rate of PYK-F can be attributed exclusively to the quinary interaction between tetrameric PYK-F and the ribosome.

### Ribosomes Do Not Alter PFK-A or LDH-A Activity

To enable analysis of the metabolic fluxes associated with glycolysis and the TCA cycle, the effect of ribosomes on the enzymatic activity of two regulatory enzymes, phosphofructokinase,^52,64^ PFK (Figure S5), and lactate dehydrogenase,^65^ LDH (Figure S6), was assessed using the direct NMR kinetic assay. PFK converts fructose-6-phosphate, F6P, to fructose 1,6-biphosphate, FBP, by irreversibly hydrolyzing ATP to ADP (Figure S5A) and cytosolic lactate dehydrogenase LDH-A, reversibly converts pyruvate to lactate by oxidizing NADH to NAD^+^ (Figure S6A). B. stearothermophilus PFK-A, which has a high degree of homology to E. coli PFK-A^64,66,67^ that conserves the active and allosteric sites, were used in these experiments. E. coli has two PFK isoforms: PFK-A, responsible for over 90% of activity in both aerobic and anaerobic conditions and allosterically regulated by PEP, and PFK-B, which contributes little to kinase activity and lacks allosteric sites. Hence, only PFK-A was characterized.

The Michaelis–Menten equation, modified to include substrate inhibition (eq 2), was used to estimate values for *K*_M_, *k*_cat_ and *K*_I_. PFK-A activity was measured by monitoring the increase in the FBP peak volume at 3.92 ppm over time (Figure S5B). Less than 10% of the starting substrate concentration was lost during the reaction dead time (Figure S5C). Kinetic parameters *K*_M_ = 84 ± 9.8 μM, *k*_cat_ = 36 ± 1.5 s^–1^ and *K*_I_ = 5.6 ± 1.2 mM were resolved for the ATP saturation curve (Figure S5D and Table S1). LDH-A activity was measured by monitoring the increase in LAC peak volume at 1.075 ppm over time (Figure S6B). Less than 10% of the starting substrate concentration was lost during the reaction dead time (Figure S6C). Kinetic parameters *K*_M_ = 12 ± 1.6 μM, *k*_cat_ = 116 ± 5.5 s^–1^, and *K*_I_ = 495 ± 74.0 μM were resolved for the PYR saturation curve (Figure S6D and Table S2).

The kinetics resolved for PFK-A were in general agreement with data obtained for PFK-A from both B. stearothermophilus and E. coli^66,68^ (Table S1). The slightly larger *K*_M_ observed at 10 °C may result from competitive binding by inorganic phosphate to the active sites, which was not present in previous assays. The kinetics resolved for LDH-A did not agree with values reported for E. coli LDH-A (Table S2) raising doubts about the source of the commercial LDH-A.^69–71^ Importantly, the activity of both PFK-A and LDH-A were unaffected by the presence of ribosomes (Figures S5E and S6E) and therefore the production of lactate from pyruvate by LDH-A is unaffected by the presence of ribosomes. A major component of the RAMBO effect results from the ribosomal external electric field, EEF, interacting with substrate dipoles at the active sites. This interaction depends on the orientation of the enzyme bound to the ribosome and is not observed at every binding site.^10,12–15^ Thus, although no evidence for an interaction between bacterial ribosomes and either PFK-A or LDH-A based on a change in enzymatic activity was found, PFK-A and LDH-A may still bind to the ribosome.

### Cam Perturbs PYK-F Binding to Ribosomes

Ribosomal-targeting antibiotics were used to probe the PYK-F-ribosome quinary structure interface.^24,25^ Disruption of the interface by antibiotic binding is expected to alter the binding affinity and/or orientation of ribosome-bound PYK-F, which would in turn alter the magnitude of the RAMBO effect observed in vitro for PYK-F. Five antibiotics were tested: chloramphenicol, Cam, erythromycin, Erm, and thiostrepton, Ths, which bind to the 50S subunit, and kanamycin, Kan, and streptomycin, Str, which bind to the 30S subunit. Concentrations were ranged from 0 to 8× of the working concentration (1×) defined as 5–10× the minimum inhibitory concentration, MIC: Cam (35 μg/mL), Erm (50 μg/mL), Ths (8.5 μg/mL), Kan (50 μg/mL) and Str (50 μg/mL).^72^

Erythromycin had no effect on PYK-F activity in either the absence or presence of 3 μM ribosomes, while increasing amounts of Ths, Kan and Str reduced PYK-F activity in both the presence and absence of ribosomes (Figure S7A). In the presence of 3 μM ribosomes, PYK-F activity increased by ∼35% (Figure 2A) and the introduction of Cam decreased PYK-F activity (Figure 2C). Inhibition of ribosome-bound PYK-F activity became highly significant (*p* < 0.0001, *d* > 2.0) at a Cam concentration of 2× (70 μg/mL). In the absence of ribosomes PYK-F activity increased by ∼10% at 6× Cam (210 μg/mL), though this activation was not statistically significant. Importantly, PFK-A and LDH-A activities were not affected by 2× Cam (Figures S5F and S6F) suggesting that Cam is a suitable candidate to perturb the PYK-F-ribosomal binding interaction for kinetic flux analysis of pyruvate metabolism.

### Ribosomal Binding Sites for PYK-F

To understand how Cam perturbs the PYK-F-ribosomal quinary binding interface, it is necessary to identify the PYK-F binding site on the ribosome and its orientation relative to the Cam binding site. Chemical cross-linking and mass spectrometry, XL-MS, analysis were used to identify possible PYK-F binding sites on intact ribosomes. Two types of homobifunctional amine-to-amine cross-linkers were tested with spacer arms 11.4 and 21.7 Å in length. Only BS­(PEG)_5_ with a 21.7 Å spacer arm produced analyzable results (Figure 3A). The purified His-tagged 55 kDa PYK-F monomer is shown in Figure 3A, lane 1. When treated with 1 mM BS­(PEG)_5_, PYK-F migrated as cross-linked oligomers (Figure 3A, lane 2). Cross-linking ribosomes reduced the intensity of bands at 70 and 100 kDa, as previously observed^10^ (Figure 3A, lane 4). Cross-linking between PYK-F and ribosomes yielded multiple faint bands larger than 100 kDa (Figure 3A, lane 6). To confirm that these faint bands contained cross-linked PYK-F and ribosomes, the reactions in lanes 2, 4, and 6 were loaded separately onto Ni-NTA beads under denaturing conditions to capture and elute His-tagged PYK-F. Lanes 2 and 7 (**E2**) were almost identical confirming that only PYK-F was present. No PYK-F was evident in lane 8 (**E4**), which contained only ribosomes and BS­(PEG)_5_. Lane 9 (**E6**) showed cross-linked PYK-F and unique bands at ∼183 and ∼126 kDa, labeled XL_1 and XL_2, respectively. Given that multiple oligomeric states were observed for PYK-F, the cross-linked mixture at XL_1 could be a PYK-F trimer (165 kDa) and ∼25 kDa ribosomal proteins, RPs, or a PYK-F dimer (116 kDa) and ∼65 kDa RPs, while XL_2 likely involves a PYK-F dimer and ∼10 kDa RPs. Hence, RPs with molecular weights of ∼10, ∼25 and ∼65 kDa were considered in the candidate library of PYK-F binding partners and submitted for proteomics analysis.

**Figure 3:**
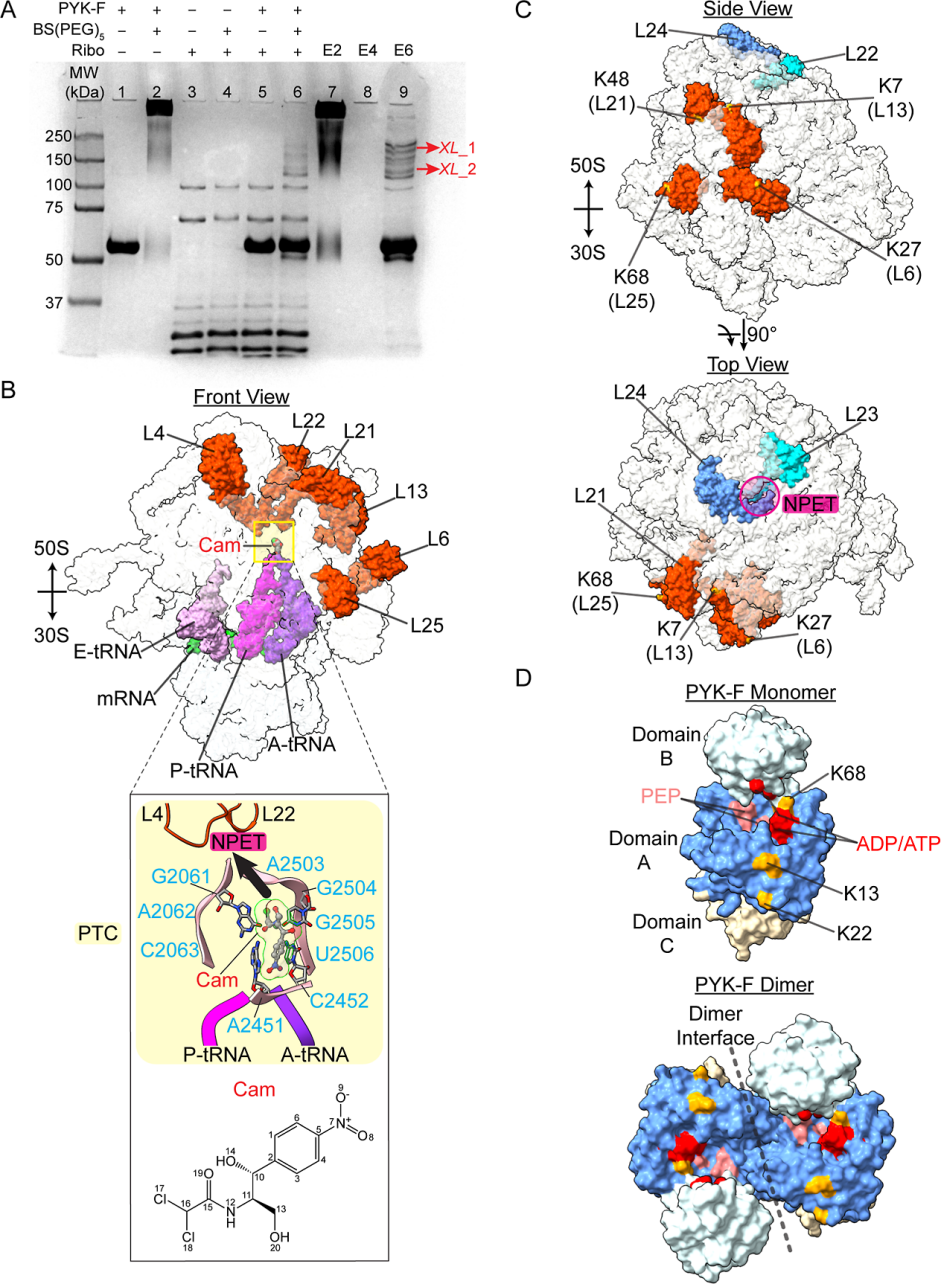
Ribosomal binding sites for PYK-F and Cam. (A) SDS-PAGE of cross-linking reactions. Purified His-tagged PYK-F without (lane 1) and with (lane 2) 1 mM BS­(PEG)_5_, purified ribosomes without (lane 3) and with (lane 4) 1 mM BS­(PEG)_5_, His-tagged PYK-F and ribosomes without (lane 5) and with (lane 6) 1 mM BS­(PEG)_5_, and cross-linked mixtures from lane 2 (E2, lane 7), lane 4 (E4, lane 8), and lane 6 (E6, lane 9) after eluting cross-linked His-tagged PYK-F from Ni-NTA affinity beads. Two bands, XL_1 and XL_2, highlighted in red in lane 9 were excised for MS analysis. (B) Ribosomal binding site for Cam at the Peptidyl Transfer Center, PTC (yellow square). Ribosome surface model (PDB entry 4V7T)^34^ showing the Cam binding site (inset) and potential PYK-F interactors RPs L4, L6, L13, L21, L22, and L25 (orange red). Ribosomal A-site (blue violet), P-site (deep pink), and E-site (plum) site tRNA and mRNA (lime) are from a cryo-EM structure of an elongating ribosome (PDB entry 6WDE).^35^ MatchMaker was used to align the peptidyl transferring ribosome onto the Cam-bound ribosome. Inset top, close-up of Cam bound to the ribosomal A-site crevice. Cam is depicted as a ball-and-stick model (C: gray, N: blue, O: red, Cl: green with 50% surface transparency). 23S rRNA bases that interact with Cam, G2061-C2063, A2451-C2452 and A2502-C2506, are in light pink ribbons, also shown are P-tRNA (deep pink ribbon), A-tRNA (blue violet ribbon), L4 and L22 (orange red). The direction of nascent peptide elongation is indicated by an arrow (black) in the direction of the Nascent Peptide Exit Tunnel, NPET (magenta). Inset bottom, chemical structure of Cam. The image was generated using ChemDraw, with atom numbers indicated following ChemDraw’s notation rules. (C) Cross-linked RPs lysine residues. Top, Cross-linked RPs L6, L13, L21, L25 (orange red), and region indicator RPs L23 (cyan) and L24 (blue) for NPET are shown on the ribosome surface (PDB entry 6BU8). Cross-linked lysine residues K27 (L13), K7 (L13), K48 (L21) and K68 (L25) are in yellow. Bottom, cross-linked RPs L6, L13, L21 and L25 (orange red) do not overlap with the NPET (magenta circle) indicated by L23 (cyan) and L24 (blue). (D) Cross-linked PYK-F lysine residues do not overlap with the active sites or the dimerization interface. Top, surface model of monomeric PYK-F (PDB entry 4YNG)^53^ showing A (blue), B (light cyan) and C (light yellow) domains. The ADP and PEP binding sites are highlighted in red and coral, respectively. Lysines K13, K22 and K68, cross-linked to RPs, are highlighted in orange. Bottom, surface diagram of dimeric PYK-F indicated with a dashed line as the dimer interface. All images were generated by using UCSF ChimeraX 1.5.^46^

The XL_1 and XL_2 bands were excised and enzymatically digested in-gel prior to analysis using a bottom-up proteomics strategy. Typical MS spectra are shown in Figure S8. RP candidates and PYK-F were input to a database for pLink 2.0 ^43^ to search for intermolecular cross-links and to identify the solvent accessibility of cross-linked residues (Table S3). Fifteen RPs L4, L6, L9, L13, L17, L21, L22, L25, L27, L31, S1, S3, S9, S12 and S20 were identified as potential PYK-F-binding partners (Figure S9), all cross-linked peptides agreed to within 4 ppm of the theoretical masses. The RPs match ∼50% of the RP candidates previously determined to bind with PYK-F in published proteomic studies (Table S4).^9,18^

Six RPs, L4, L6, L13, L21, L22 and L25 are located close to the peptidyl transfer center, PTC of the ribosome, which is the binding site for Cam (Figure 3B). Most of these RPs are core components of the 50S subunit and contribute to its assembly.^73^ L4, L20 and L22 bind to the 5′ end of the 23S rRNA, while L13 and L21 bind to L20, which is associated with the 23S rRNA, all are involved in the early stages of 50S assembly. L6 binds to the 23S rRNA during the late stage of 50S assembly and L25 binds to the 5S rRNA, mediating its contact with the 23S rRNA. The extended loop regions of L4 and L22 line the nascent peptide exit tunnel, NPET.^25,74^ L4 and L22 are also determinants of the erythromycin binding site on the ribosome. Because Erm did not affect PYK-F activity in either the absence or presence of intact ribosomes in vitro, it is unlikely that L4 and L22 are involved in the PYK-F-ribosomal interaction. Therefore, L6, L13, L21 and L25 are the most likely candidate RPs to participate directly in the PYK-F-ribosomal binding interface (Figure 3C).

Cam binds to the ribosomal A-site crevice with ∼2.8 μM binding affinity blocking peptidyl transfer and inhibiting protein synthesis.^24,75^ This catalytic crevice includes ribonucleotides G2061-C2063, A2451-C2452 and A2503-U2508, with a surface pocket formed by the bases of G2061, A2451, C2452 and G2504,^25,34^ which contact Cam and are stabilized primarily by π-stacking interactions involving C2452. The positioning overlaps with the placement of incoming aminoacyl-tRNAs thereby obstructing the accommodation of aminoacyl-tRNA in the PTC active site (Figure 3B).^34,75^

Overall, four distinct cross-linked complexes were identified, placing residue pairs K27­(L6)-**K68** (PYK-F), K7­(L13)-**K13** (PYK-F), K48­(L21)-**K68** (PYK-F) and K68­(L25)-**K22** (PYK-F) within 21.7 Å of one another. These putative candidate RPs are concentrated on the top of the L7/L12 stalk region, making it unlikely that PYK-F binding to these RPs blocks the exit site of the NPET or competes with binding of the translational folding components, including L23, L24 and the trigger factor^76^ (Figure 3C).

Each PYK-F monomer consists of three domains.^36,63^ Domain A is sandwiched between domains B and C and is involved in dimerization, while domain C mediates tetramerization. Cross-linked residues K13, K22 and K68 are located in domain A, which is close to R32 and N34–H37 of domain A and R73 and K156 of domain B, the binding site for ADP/ATP, and K220, G245, D246 and T278 of domain A, the binding site for PEP (Figure 3D).^36,53^ The location of the cross-linked residues suggests that binding to the ribosome is unlikely to disturb the association equilibria between monomers, dimers and tetramers, or with the binding of substrates to the active sites. Confirmation of the exact orientation will require in vitro analysis and modeling to determine the geometry of the binding interface.^10^ However, the close proximity of the active sites to the PYK-ribosome quinary interface suggests that the ribosomal EEF may interact with substrates in the active sites to elicit the RAMBO effect.^10,12–14^

### Real Time NMR Monitors Living E. coli during Mixed Acid Fermentation

Alginate encapsulated E. coli BL21­(DE3) cells^27^ were placed into the bioreactor and incubated with [U–^13^C]-glucose in hybrid growth medium salts buffer, HGM, salts to produce labeled metabolites (Figure 4A). Proton-decoupled one-dimensional ^31^P spectra, Pre-^31^P, were recorded for the first 3.5 h of the experiment. A two-dimensional heteronuclear single quantum coherence, ^1^H–^13^C HSQC, spectrum^77^ was acquired over the next 6.5 h to identify and assign metabolites derived from the isotopically labeled glucose (Figure 4B–D). Proton-decoupled ^31^P spectra, Post-^31^P, were recorded for the final 3.5 h of the experiment. The ^13^C–^13^C couplings observed in the carbon dimension ensured the metabolic products were catabolized from uniformly ^13^C_6_-labeled glucose.

**Figure 4:**
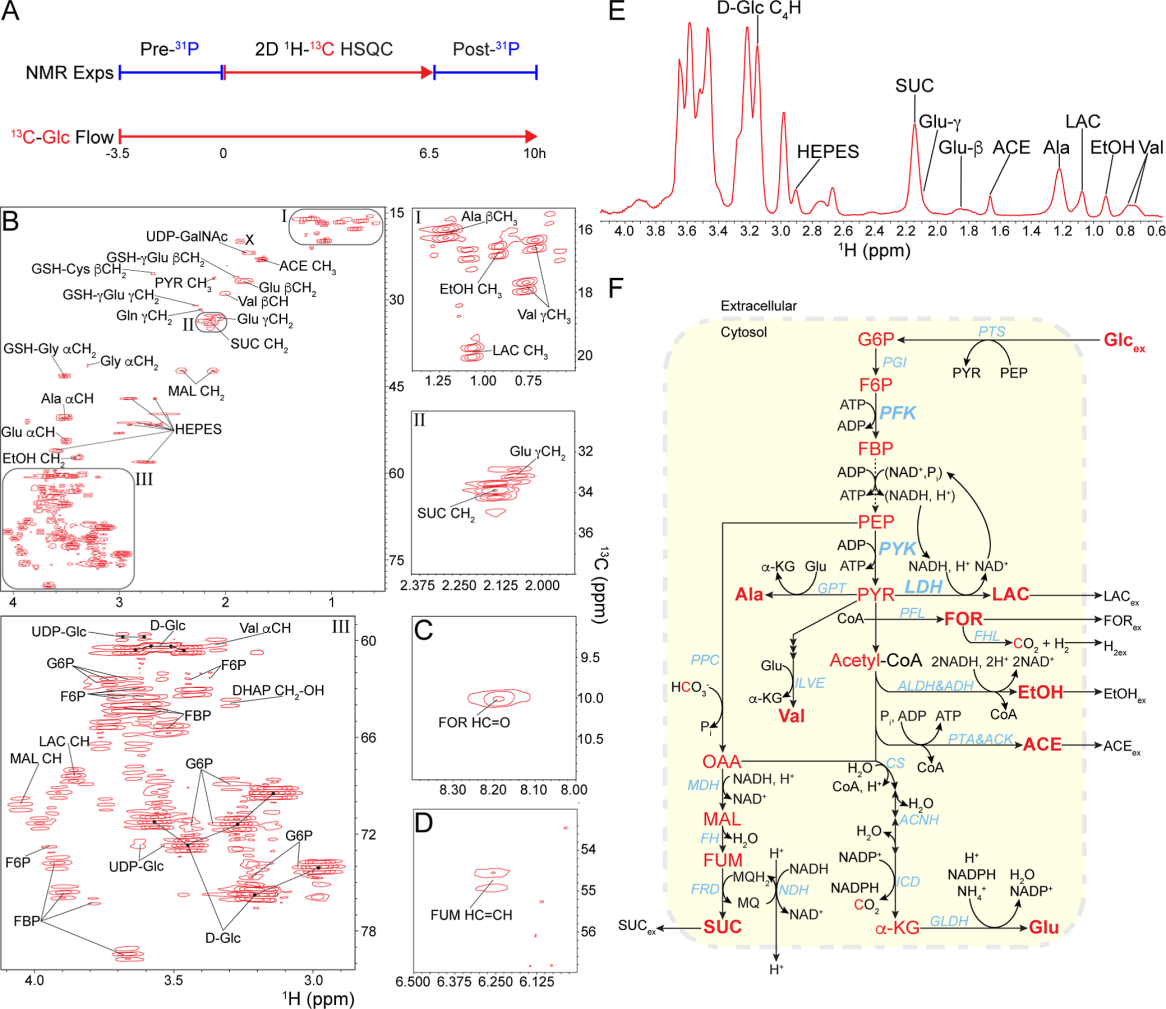
Metabolic profiling of encapsulated E. coli cells. (A) Schematic of continuous ^13^C glucose flow and ^1^H–^13^C HSQC NMR experiments used to determine intracellular metabolites. (B) 2D ^1^H–^13^C HSQC NMR spectrum of identified intracellular metabolites. (C) Formate, FOR, was detected outside of the main spectrum shown in panel B. (D) Fumarate, FUM, was observed at a much lower contour level compared to panels B and C, also displayed outside the main spectrum. (E) Major metabolites identified in the envelope of 2D ^1^H–^13^C HSQC spectrum in panel B are indicated: Glc, LAC, EtOH, ACE, SUC, Ala, Val, and Glu. (F) Mixed acid fermentation of E. coli BL21­(DE3) fed with glucose and NH_4_Cl inside NMR tube at 283 K. Major metabolites identified in panel C and E are bolded. ^13^C-labeled metabolic intermediates from extracellular glucose, Glc_ex_, are indicated in red. Metabolic enzymes are displayed in light blue. Abbreviations: ACE, acetate; ACK, acetate kinase; ACNH, aconitate hydratase B; ADH, alcohol dehydrogenase; Ala, alanine; ALDH, acetaldehyde dehydrogenase; CS, citrate synthase; d-Glc, d-glucose; DHAP, dihydroxyacetone phosphate; EtOH, ethanol; FH; fumarate hydratase B, anaerobic; FHL, formate hydrogen lyase complex; FRD, fumarate reductase; GLDH; NADP-specific glutamate dehydrogenase; Gln, glutamine; Glu, glutamate; Gly, glycine; GPT, glutamate-pyruvate aminotransferase; GSH, glutathione; G6P, glucose 6-phosphate; ICD isocitrate dehydrogenase; ILVE, branched-chain-amino-acid aminotransferase; LAC, lactate; MAL, malate; MDH, malate dehydrogenase; NDH, NADH dehydrogenase I PAT, phosphate acetyltransferase; PFL, pyruvate-formate lyase; PGI, phosphoglucose isomerase; PPC, phosphoenolpyruvate carboxylase; PTS, glucose-specific phosphotransferase system; SUC, succinate; UDP-GalNAc, UDP-Glc, UDP-glucose; UDP-GalNAc, UDP-*N*-acetylglucosamine; Val, valine; and an unknown X triggered by Cam treatments.

The combination of intracellular and extracellular metabolites identified revealed that the metabolic phenotype of the BL21­(DE3) cells was consistent with mixed acid fermentation, characterized by the excretion of multiple acidic end-products (Figures 4E,F, and S10).^30,54,78,79^ Fermentation was anticipated because the growth medium contains ∼260 μM dissolved oxygen at standard conditions,^7^ which is insufficient to meet the metabolic demands of the cells and support rapid growth. Oxygen will be quickly consumed by cells at the outer surface of the beads, leaving most of the internalized cells under anaerobic conditions. Since NMR measures the average metabolic state, the phenotype represents the collective contribution of the encapsulated cells. As a facultative anaerobe, E. coli can grow on HGM supplemented with glucose as the sole carbon source and NH_4_Cl as the sole nitrogen source by utilizing mixed acid fermentation in the absence of exogenous electron acceptors, such as oxygen.^80,81^ E. coli does not contain cytosolic hexokinase but instead transfers extracellular glucose, Glc_ex_, into the cytosol through the phosphotransferase system, PTS, coupled to a phosphorylation cascade powered by PEP and converting transported glucose into G6P.^82^ Hence, G6P is the starting point for glycolytic metabolism in the cytosol and is further oxidized via the Embden–Meyerhof–Parnas, EMP, pathway, rather than the Entner–Doudoroff, ED, pathway, into pyruvate (Figure 4F).

### Real Time Pulse Chase NMR

Alginate encapsulated E. coli BL21­(DE3) cells were placed into the bioreactor and the flow of fresh HGM salts supplemented with ^12^C-glucose and NH_4_Cl at 200 μL/min^26^ was initiated. To assess the energy state of the cells, proton-decoupled ^31^P spectra were recorded for the first 3.5 h of the experiment. A ^13^C-glucose pulse was initiated at time *T*_0_ and lasted for approximately 3 h followed by a ^12^C-glucose chase. The pulse-chase took approximately 6.5 h to complete during which time a total of 500 one-dimensional ^13^C-edited proton NMR spectra were acquired to monitor the appearance and disappearance of metabolites with proton-attached ^13^C bonds corresponding to a temporal resolution of 47 s for the metabolic fluxes. ^31^P spectra were recorded for the final 3.5 h of the experiment (Figure 5A). Projection of two-dimensional ^1^H–^13^C HSQC cross peaks onto the proton dimension identified 8 metabolites that produced nonoverlapping signals of sufficient strength for quantitative analysis (Figures 5B and 4E). The ^13^C-edited proton spectra included the peaks resolved for the CH of formate, FOR (8.19 ppm), CH_2_ of succinate, SUC (2.14 ppm), γ-CH_2_ of glutamate, Glu-γ (2.09 ppm), CH_3_ of acetate, ACE (1.66 ppm), β-CH_3_ of alanine, Ala (1.22 ppm), CH_3_ of lactate, LAC (1.07 ppm), CH_3_ of ethanol, EtOH (0.92 ppm), and γ-CH_3_ of valine, Val (0.74 ppm).

**Figure 5:**
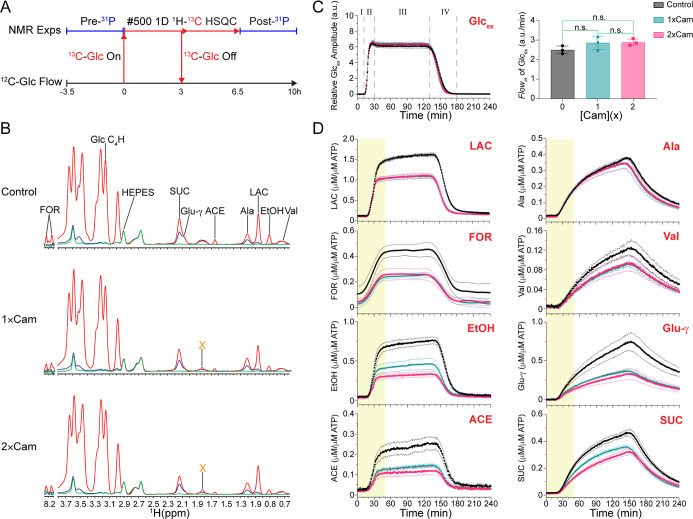
Kinetic flux profiles of intracellular metabolites. (A) Experimental procedure for pulse-chase analysis of living cells by NMR spectroscopy. (B) Superimposed 1D ^13^C-edited proton HSQC metabolite spectra from the early (green), middle (red) and late (blue) portion of the kinetic flux profile within the ^13^C-pulse window in the absence and presence of 1 × Cam and 2 × Cam. An unknown metabolite X was induced by Cam treatment. (C) Left, normalized extracellular glucose, ^13^C-Glc_ex_, pulses. Glucose flow occurs in four phases: (I) prepulse, (II) incorporation (leading edge), (III) plateau, and (IV) clearance (trailing edge). Right, flow rate of ^13^C-Glc_ex_ without and with Cam. (Error bars represent mean ± SEM; n.s., nonsignificant). (D) Kinetic flux profiles of intracellular metabolites resulting from ^13^C-Glc metabolism. The first 50 min of the pulse following initiation at *T*_0_ were modeled (highlighted) corresponding to ∼30 min of Cam exposure. All kinetic flux profiles and the^13^C-glucose profile were displayed as mean (solid points) ± SEM (transparent dashed lines) from at least three independent trails. The kinetic flux profiles are displayed in micromolar per micromolar ATP.

Initially only the natural ^13^C abundance of metabolites and HEPES from the HGM buffer were observed. The chemical shift and intensity of the HEPES peak at 2.91 ppm did not change over time and was not internalized by the cells, making it an ideal internal reference. Within 30 min of initiating the pulse the ^13^C-labeled glucose metabolic products of glycolysis and the TCA cycle were evident (Figure 5C,D) and by the end of the 3 h ^13^C pulse lactate, formate, ethanol and acetate had largely disappeared while alanine, valine, glutamate and succinate persisted until the end of the 6 h run. The leading edge of the extracellular ^13^C-Glc_ex_ pulse was modeled as a one-phase association (eqs 8–10), to yield estimates for the rate constant, *K*, and the plateau value (Figure S11 and Table S5).

Treating the cells with Cam did not grossly change the flux profile but resulted in smaller plateau values that reflect the steady state concentrations for all metabolites except alanine (Figure 5D). Glucose uptake remained constant at 30–35% in the absence and presence of Cam and the introduction of Cam did not alter the time scale of the ^13^C-glucose pulse nor did it alter the glucose uptake (Figure S12A) indicating that the phosphotransferase system, PTS, of the cells was not affected by Cam. Therefore, the proportion of PEP engaged in the PTS, ∼70% under aerobic conditions, remained unchanged, which will result in the same level of PYR with and without Cam treatments.^83^ The amount of Cam retained by the cells, ∼20%, was determined by monitoring the H16 proton peak of Cam at 6.02 ppm in the flow through (Figure S12B,C)^84^ corresponding to an intracellular concentration of ∼7 μg/mL and ∼14 μg/mL for 1 × Cam and 2 × Cam, respectively. Note that the minimum inhibitory concentration, MIC, for Cam is 6 μg/mL, ∼20 μM.^85^ Under conditions where the Cam concentration is near the MIC, the survival rate of E. coli remains high even after prolonged incubation. This is because Cam is a bacteriostatic antibiotic that does not immediately kill the cells but inhibits protein synthesis allowing the cells to survive at a diminished growth rate.^52,85–88^ Treating the cells with Cam resulted in an unidentified singlet peak, X, that overlapped with the β-CH_2_ peak of glutamate (Figure 5B). At higher Cam concentrations both the intra- and extra-cellular concentrations of the unknown metabolite increased (Figure S12D).

Except for alanine, valine, and glutamate, no other amino acid biosynthesis was detectable. Ala, and Val are both directly synthesized from PYR, while Glu, is maintained at millimolar concentrations in the cytosol as a universal amino donor.^29,89^ Glutamate dehydrogenase, GLDH, catalyzes the formation of Glu from α-KG, with nitrogen assimilation in the form of NH_4_^+^ coupled with NADPH.^90,91^ Given that the ammonia concentration in the HGM medium is almost 10-fold higher than the *K*_M_ for GLDH, and the activity of GLDH is nearly 100% even at 4 °C,^90^ and the flux is favored in the forward direction toward glutamate synthesis. The contribution of gluconeogenesis and the backward reaction was neglected since glucose was continuously supplied to the cells.

### Cam Affects Substrate-Level Phosphorylation, SLP

To assess the energy status of the cells over the course of the experiment proton-decoupled ^31^P spectra were collected prior to and after introducing the ^13^C-glucose pulse. Phosphate-containing biomolecules, including phosphorylated sugars and nucleotides, inorganic phosphate, phospholipids and redox couples NAD­(H) and NADP­(H) were identified (Figure 6A). Free extracellular inorganic phosphate, P_i_^Ex^, exhibited the highest intensity peak. The cytosolic inorganic phosphate peak, P_i_^Cyt^, had the second-strongest intensity and resonated at nearly the same chemical shift as that of P_i_^Ex^. No changes in the chemical shifts of P_i_^Cyt^ and P_i_^Ex^ were observed in either the absence and presence of 2 × Cam, indicating that both cytosolic and extracellular pH were maintained over the course of the experiments.^82,92,93^

**Figure 6:**
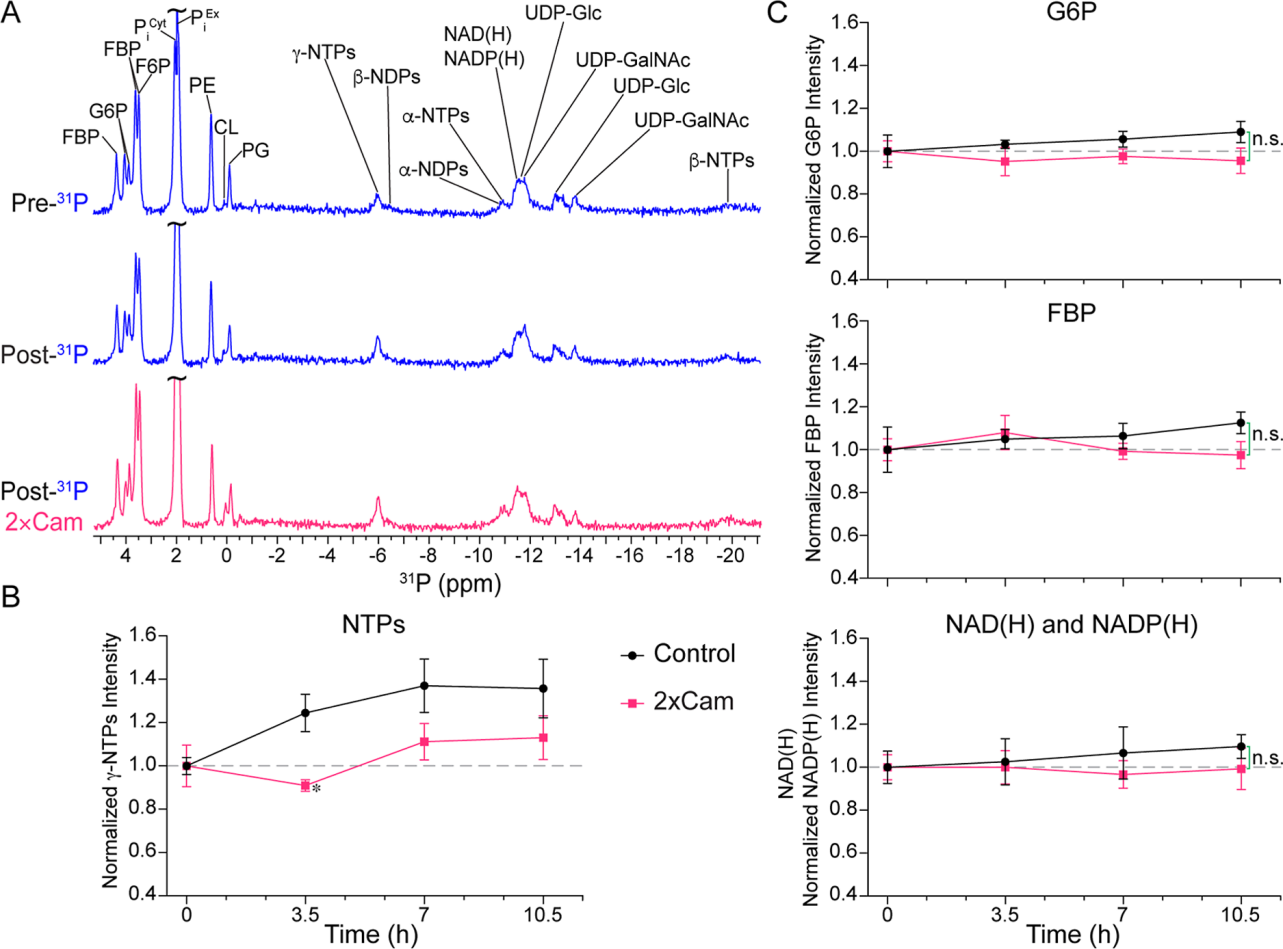
^31^P spectra and energy status of cells. (A) Phosphate-containing biomolecules of E. coli under Pre-^31^P, Post-^31^P, and Post-^31^P treated with 2 × Cam. Abbreviations: FBP, fructose-1,6-biphosphate; G6P, glucose-6-phosphate; F6P, fructose-6-phosphate; P_i_^Cyt^, cytosolic inorganic phosphate; P_i_^Ex^, extracellular inorganic phosphate; γ-NTPs, γ-phosphate group of ribonucleotide triphosphates; β-NDPs, β-phosphate group of ribonucleotide diphosphates; β-NTPs, β-phosphate group of ribonucleotide triphosphates; α-NDPs, α-phosphate group of ribonucleotide diphosphates; α-NTPs, α-phosphate group of ribonucleotide triphosphates; NAD­(H), oxidized (and reduced) forms of nicotinamide adenine dinucleotide; NADP­(H), oxidized (and reduced) forms of nicotinamide adenine dinucleotide phosphate; UDP-Glc, UDP-Glucose; UDP-GalNAc, UDP-*N*-acetylglucosamine; PE, phosphatidylethanolamie; PG, phosphatidylglycerol; CL, cardiolipin. These three phospholipids were determined based on their relative composition in E. coli.^94^ (B) Time-course of NTPs level with and without 2 × Cam treatment. The lines were added for ease of viewing and error bars represent mean ± SEM from three independent trails. (*, *p* < 0.05 and *d* > 1.0). (C) Time-course measurements of G6P, FBP and NAD­(H) plus NADP­(H) levels with and without 2 × Cam treatments. Lines were added for clarity and error bars represent mean ± SEM from three independent trails. n.s., nonsignificant.

The viability of the encapsulated cells was confirmed from the presence of glycolytic intermediates G6P, F6P and FBP. The size of the G6P and FBP pools were notably larger than other phosphorylated intermediates, essential for a high glycolytic flux and in accordance with intracellular concentrations estimated to be in the millimolar range under both aerobic and anaerobic conditions.^29,81,82,89,95^ The intracellular concentrations of NAD­(H) and NADP­(H) were the highest among the nucleotides, consistent with elevated levels of NAD^+^ enhancing the flux of anaerobic glycolysis.^82^ Unlike NTP levels that increased slightly over time both in the absence and presence of 2 × Cam (Figure 6B), G6P, FBP, NAD­(H) and NADP­(H) levels remained unaltered (Figure 6C).

The constant levels of FBP and G6P upon Cam treatments indicate that the activity of PFK-A is not affected by Cam in vivo, consistent with the outcomes of the in vitro assay (Figure S5E,F). The persistent high level of FBP imparts the same degree of allosteric activation to downstream PYK-F and phosphoenolpyruvate carboxylase, PPC, activity, and therefore does not contribute to changes in PYK-F or PPC activity upon Cam addition in vivo. Other glycolytic enzymes, upstream of PYK-F, lack direct evidence of ribosomal binding in E. coli (e.g., ALD and PGM, Figure 1) or exhibit ribosomal binding sites that are distinct from that of PYK-F (e.g., TPI, GAPDH, PGK, Table S4) indicating that Cam may not influence the activity of these enzymes. Collectively these observations suggest that the PEP pool size is maintained at a constant level.

Three phospholipids, phosphatidylethanolamine, PE, phosphatidylglycerol, PG, and cardiolipin, CL were identified based on their relative composition in E. coli: ∼77% PE, ∼21% phosphatidylglycerol, and ∼1% cardiolipin,^94^ and two major precursors derived from G6P^29,54,89^ that are involved in the biosynthesis of cell wall components, UDP-glucose, UDP-Glc, and UDP-*N*-acetylglucosamine, UDP-GalNAc, were also detected (Figure 6A). The identification of sugar phosphates, phospholipids and cell wall biosynthesis precursors suggests a high degree of structural integrity, catabolic and anabolic activity, and cell viability.

The most important indicator of energy status, ATP, serves as a universal energy currency inside living cells.^27^ However, the γ-phosphate groups of the other NTPs, which are also maintained in the millimolar range inside E. coli, all resonate at −6 ppm.^29,89^ thereby rendering the γ-ATP peak unresolvable from other γ-NTPs. Hence, the singlet at −6 ppm represents the total pool of NTPs (Figure 6A). The differences in peak intensity between γ-NTPs and β-NDPs were consistent with the intracellular ATP/ADP ratio of 8–15:1 observed during fermentation.^81,82,96^ ATP levels within the total NTP pool is 40–55% under aerobic respiration where ATP acts as a universal precursor for synthesis of the other NTPs.^89,97–99^ Changes in ATP levels directly affect the overall NTP pool, which undergoes a considerable decline during anaerobic fermentation. However, because ATP is rapidly generated by substrate-level phosphorylation to a greater extent than the other NTPs^82,96^ even under anaerobic conditions, ATP will still constitute the majority of the NTP pool at a ratio comparable to that observed under aerobic conditions due to its irreplaceable role in cellular maintenance and biomass synthesis. Note that the overall NTP pool is diminished when E. coli is grown at 10 °C due to reduced metabolic activity and a slower growth rate.^57^

The intensity of the γ-NTP peak decreased steadily in the presence of 2 × Cam relative to untreated cells during the 3 h pulse phase reaching a significant, ∼30%, decrease before increasing over the next 7 h during the chase phase where it remained ∼15% below control levels (Figure 6B). This observation is consistent with in vitro results that showed PYK-F activity decreased significantly in the presence of ribosomes upon introducing 2 × Cam (Figure 2C) and can be explained by the ongoing inhibition of protein synthesis resulting from the binding of Cam to ribosomes in the early chase phase leading to reduced ATP and GTP consumption.^85,87,98^ In the late chase phase as Cam is depleted PYK-F will reassociate with ribosomes resulting in RAMBO-induced ATP generation. Both mechanisms enhance the level of ATP as reflected in the overall NTP levels. Given that PYK-F catalyzes the last irreversible step of glycolysis for ATP generation, and that substrate-level phosphorylation, SLP, drives ATP levels during anaerobic fermentation, a decreased RAMBO effect is expected to reduce SLP.^99^ Because ATP dominates the NTP pool, the diminished efficiency of SLP and consequent reduction in NTP levels can be attributed to Cam.

Overall, the glycolysis pathway remains well-balanced up to PEP, but treating the cells with Cam affected SLP (Figure 6B). Notably, changes in SLP should not be attributed to a reduction in the number of cells following Cam treatment, as the pools of other phosphate-containing biomolecules remain unchanged with the introduction of Cam. Given that the effective concentration of Cam in this experiment is close to the MIC, previous studies have shown that even at 4–5 × MIC, cells can maintain over 90% viability after 2 h of Cam exposure.^85,87^ This evidence strongly suggests that the observed decrease in SLP is primarily due to the direct impact of Cam on PYK-F activity and its subsequent effects on ATP production, rather than a significant loss of cell viability. The consistency of peak patterns in pre- and post-^31^P spectra validates the efficacy of the NMR bioreactor to maintain cell viability.

### Kinetic Flux Analysis of Pyruvate Metabolism Validates the RAMBO Effect In Vivo

To demonstrate the RAMBO effect in vivo, results from the ^31^P experiments must be corroborated with real-time metabolic flux analysis. All metabolites observed are downstream from pyruvate (Figure 4). Because no PYR could be detected on the 1D spectrum due to its small pool size, the influx rate of PYR was assumed to be equal to its efflux rate. Accordingly, the overall PYR efflux was estimated by combining the influx rates of downstream metabolites using flux balance equations under metabolic steady state.^26,100–102^

The profiles shown in Figure 5D are scaled data averaged from three independent experiments and grouped into two categories: predominantly secreted and predominantly intracellular. The first exhibits a pulse shape similar to that of ^13^C-Glc_ex_ (Figure 5A,C), which includes LAC, FOR, EtOH and ACE. These metabolites are all end-products of pyruvate metabolism and are secreted from the cell (Figure S10), resulting in relatively small intracellular pools that quickly reach plateau levels.^28^ The second consists of amino acids and SUC. These metabolites generate larger intracellular pools that are continuously channeled into anabolic pathways, causing the signals to rise more slowly and requiring much longer times to reach isotopic steady state.^28^

The rapid exchange system in the NMR bioreactor, removes extracellular metabolites from the 200 μL NMR window volume at a rate of 200 μL/min achieving a turnover rate of 0.5–1 min^–1^.^26^ Comparing this with the fastest influx rate of lactate, which equals the rate of excretion (∼0.13 min^–1^, Table S6), it is evident that the excretion rate is much slower than the turnover rate of exchange system. This indicates that only intracellular metabolites labeled with ^13^C were observed during the RTPC-NMR experiments. Hence, the rates shown in Figure 7A reflect the ^13^C influx of intracellular metabolites in the absence and presence of 1× and 2× Cam.

**Figure 7:**
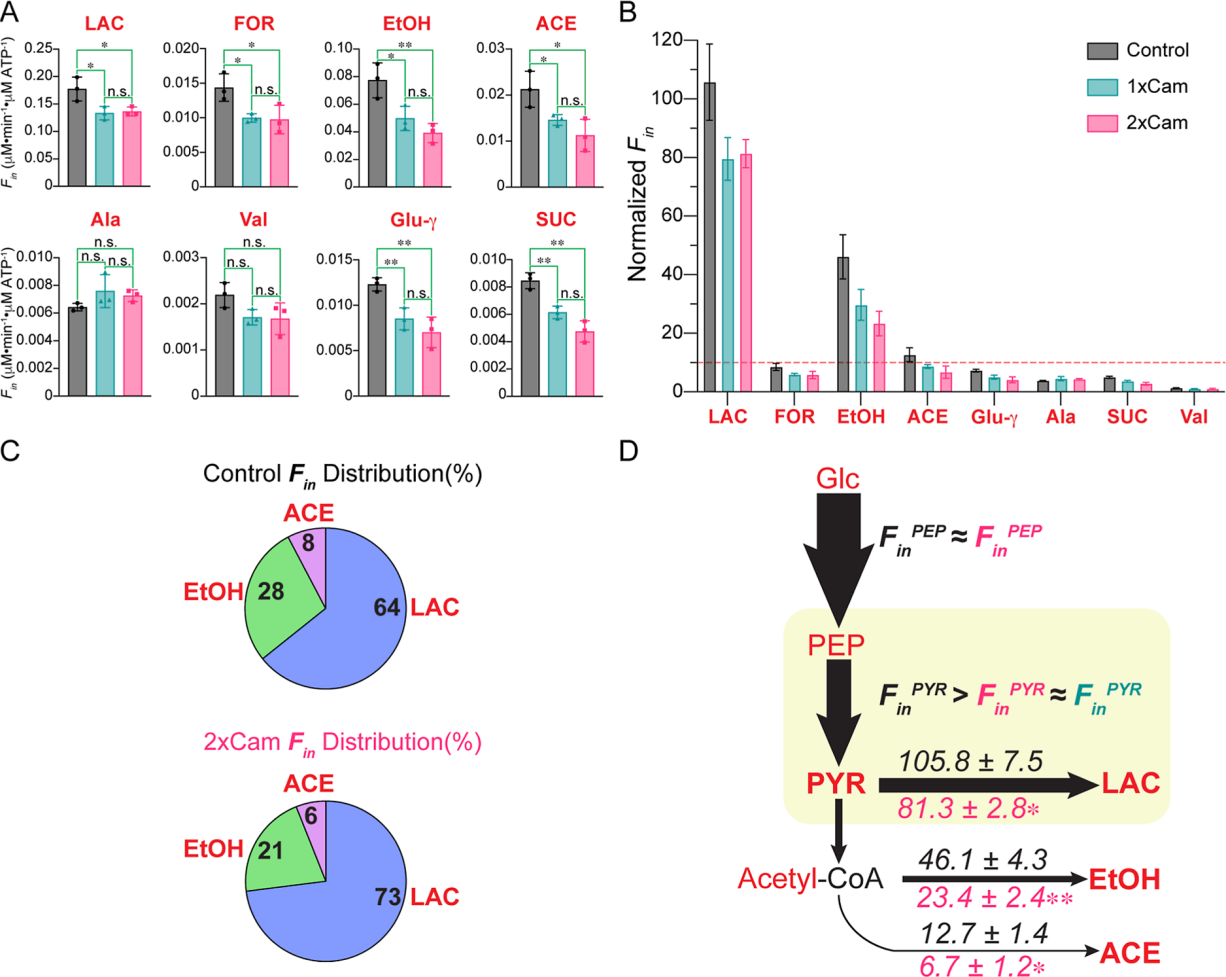
Flux analysis of Pyruvate metabolism. (A) Influx rate, *F*_in_, of intracellular ^13^C-metabolites. Relative *F*_in_ values are displayed in bar diagrams for control, 1 × Cam and 2 × Cam treatments. (B) Normalized influx rates relative to the lowest rate observed (*F*_in_ for Val at 2 × Cam). A value of 10 was set as a threshold (light red dash line). Error bars represent mean ± SEM. (C) Distribution of metabolite influx rates above the threshold for control (top) and 2 × Cam (bottom). (D) Flux analysis of PYR without (black) and with 2 × Cam (light pink) treatment. The size of arrows corresponds to the ratio of *F*_in_ values shown in panel C. Values are mean ± SEM, with significance levels indicated as *, *p* < 0.05 and *d* > 1.0; **, *p* < 0.01 and *d* > 1.0; n.s., nonsignificant.

Cam-induced metabolism can lead to a significant increase in ATP levels due to the inhibition of protein synthesis, which typically consumes over 60% of the ATP generated by the cell.^85,87,103^ A significant decrease in NTP’s was observed in the first 3 h of Cam treatment relative to control cells followed by an increase over the next 3 h comparable to what was seen for control cells (Figure 6B). The exact timing of the Cam-induced increase during the pulse phase cannot be precisely determined because NMR only detects the average concentration present in the target volume. However, the early decrease suggests that a primary metabolic response to Cam was the reduction of SLP, likely due to disruption of the PYK-F-ribosome interaction. To maximize the separation between the Cam-induced decrease and increase in the NTP pool, only the first 50 min of the kinetic flux profiles, corresponding to 30 min of Cam exposure, were modeled as a one-phase association (eq 8, Figures S13–S20 and Table S6–S13). This 30 min window is consistent with the Cam exposure times performed in vitro assays and reported in most studies.^85–87^ Identifying the temporal course of ATP levels is important because a significant increase would lead to allosteric inhibition of PYK-F making it difficult to distinguish from a diminished RAMBO effect.

The end-products of pyruvate metabolism, LAC, FOR, EtOH and ACE, all exhibited a consistent and statistically significant reduction in influx rates when E. coli were exposed to Cam. In contrast, the amino acids displayed variable responses: the influx rates of Ala and Val were unaffected by Cam despite being directly associated with PYR. This suggests that aminotransferase activity is regulated by factors other than precursor levels, possibly due to feedback inhibition by the amino acids themselves given the larger ^12^C pool and slower consumption rate at the low experimental temperature. On the other hand, the influx rate of Glu-γ and SUC mirrored the reductions observed in LAC, FOR, EtOH and ACE in response to Cam exposure implying that the turnover rate of Glu and SUC is closely linked to the carbon flow rate from PYR. Note that both Glu and SUC are key intermediates of the TCA cycle. Although E. coli maintains a high intracellular concentration of Glu, which acts as a universal amino donor and is integral to biomass synthesis, its rate of consumption is much faster than that of Ala and Val. This could be due to the activation of NADP-specific glutamate dehydrogenase, GLDH, in the forward direction to generate Glu, driven by the high ammonia concentration in the hybrid growth medium. Note that no dose dependence was observed for any metabolites. This is in line with the observation that Cam is rapidly concentrated two to 4-fold inside cells^104^ effectively saturating the RAMBO effect (see Figure 2C).

To evaluate the changes in the glycolytic flux resulting from Cam binding to the ribosome, the influx rates were normalized to the lowest influx rate observed, Val at 2 × Cam (Figure 7B), and a threshold of 10% of the influx rate of LAC was set. The influx rate of LAC exceeded the threshold by ∼9-fold followed by EtOH at ∼3-fold, and ACE, which just reached the threshold. The influx rates of LAC, EtOH, and ACE were used to assess the effect of Cam on the PYR flux. The proportional distribution of these influx rates showed that the influx of LAC consistently remained the predominant factor in both the absence and presence of Cam treatments (Figure 7C). The influx rates of the three metabolites decreased in the presence of Cam aligning with experimental expectations, however the changes were not uniform: LAC decreased by 23% while EtOH and ACE decreased by 49% and 47% respectively (Figure 7D). In the absence of Cam, the sum of the influxes was 164.6 ± 13.2 and in the presence of 2 × Cam that value dropped to 111.4 ± 6.4 representing 32% decrease in PYR-F activity when PYR-F binding to the ribosome is restricted. The percentage reduction for all the metabolite fluxes is somewhat greater than the ∼10% decrease in *k*_cat_ observed in the absence of ribosomes in vitro (Table 1) suggesting the possibility of feedback regulation by decreasing ATP levels immediately following introduction of Cam.

## Discussion

The binding of PYK-F to ribosomes results in an increase in kinetic activity in vitro due to the RAMBO effect. This effect is abated by the ribosomal binding antibiotic chloramphenicol. Analysis of metabolic fluxes in E. coli glycolysis using RTPC-NMR spectroscopy revealed a decrease in PYK activity in the presence of Cam consistent with the loss of the RAMBO effect and validating the phenomenon in vivo. Because pyruvate flux could not be detected, lactate production was used as an indicator of pyruvate activity. The activity of lactate dehydrogenase A, LDH-A, which converts pyruvate to lactate and was previously shown to be detectable by RTPC-NMR spectroscopy,^26^ was characterized in vitro. The activity was unaffected by the presence of ribosomes or Cam validating the use of lactate flux to assess pyruvate activity.

Unlike the majority of enzymes in glycolysis that exhibit steady levels of activity, the two rate-limiting enzymes in E. coli glycolysis, PFK and PYK, are subject to allosteric regulation that can increase or decrease basal activity depending upon prevailing metabolic conditions. Control experiments showed that NTP production increased significantly over the first 3.5 h before reaching steady state levels. In the presence of Cam the production of NTPs dropped significantly over the first 3.5 h while the increase in NTP production over the remainder of the experiment was similar for the cells in both the absence and presence of Cam indicating no allosteric modulation in PFK and PYK activity. Thus, the loss of the RAMBO effect can be attributed to alteration of the PYK-ribosome quinary structure.

It was estimated that the cells were in early log phase during the pulse-chase analysis, meaning that protein synthesis was active and the RAMBO effect observed was based on ribosomes functioning under normal physiological conditions.^105^ This hypothesis is supported by the continuously rising NTP levels observed during the first 7 h as protein synthesis consumes significant amounts of ATP and GTP, and the synthesis of its substrates, including mRNA and tRNA, requires a large NTP pool size to sustain these biochemical reactions.^95^ Ribosomal translation is primarily driven by GTP hydrolysis, particularly during elongation, while ATP consumption mainly supports overall cellular energy demands and tRNA charging. As a result, both ATP and GTP levels exhibit a similar trend in relation to ribosome function, where inhibition of ribosomal activity results in a significant increase in both ATP and GTP levels.

Since only ATP is directly involved in central carbon metabolism, and its level is closely related to PYK activity, monitoring ATP levels alone was deemed sufficient to effectively assess changes in ribosome function and PYK activity. It should also be noted that the change in the RAMBO effect on PYK activity upon exposure to Cam occurred much faster than the inhibition of ribosomal translation activity. As shown in Figure 6B, the overall NTP levels decreased rapidly, primarily due to reduced ATP generation resulting from the deactivation of the RAMBO effect on PYK activity. Therefore, this study focused on fast metabolic responses rather than the slower changes associated with ribosome translation inhibition.

Structural studies have shown that neither the occupancy of the A and P sites by deacylated tRNAs, nor the presence of mRNA, alters the binding of Cam to the 70S ribosome.^106^ Moreover, high-salt washing does not compromise the integrity of the 70S ribosome, as demonstrated in previous work.^9,10^ In living cells, the structure of 70S ribosomes has been visualized using cryo-electron tomography, and the structural dynamics of translation classified into 13 distinct conformational states.^107^ Upon Cam treatment for a short, ∼20 min, duration, it was observed that over 70% of ribosomes become trapped in a single state immediately preceding the peptidyl transfer reaction, due to Cam’s inhibitory effect.^107^ We propose that Cam binding to vacant 70S ribosomes may trap the ribosomes in a stalled conformation that is unfavorable for quinary interactions between PYK-F and its putative ribosomal protein, RP, binding partners, as identified in Figure 3C. It is important to note that these putative RPs are not directly involved in translation but instead contribute to the structural stabilization of the 50S subunit.

Based on the experimental conditions of this study, which include low temperature, low oxygen levels and the reported relationships between these parameters,^58–60^ the observed fluxes were minimally affected by factors related to cell growth. These factors, such as increased cell number and morphological changes, as well as bead volume expansion, were considered negligible. At low temperatures in glucose minimal medium, the cell doubling time is ≥10 h. This was further increased by low oxygen levels, extending the doubling time to ∼30 h due to the low efficiency of ATP generation via fermentation.^30,57–60^ Therefore, the time scale of the RTPC-NMR experiment was significantly shorter than the cell doubling time.

## Conclusions

The fluxes observed for lactate production aligned with experimental predictions and in vitro findings to confirm that the RAMBO effect occurs in vivo. The results are consistent with ribosomal regulation of glycolysis in living cells, where ribosomes may act as organizing centers, analogous to eukaryotic supramolecular assemblies, functioning to compartmentalize and coordinate metabolic pathways.^6,10^

## Supplementary Material



## Notes

Figure S1. Quality control of cell beads and modified RTPC-NMR bioreactor setup. Figure S2. Metabolite calibration curves. Figure S3. Reduction of ATPase and dehydrogenase contamination in ribosome preparations. Figure S4. PYK-F purification. Figure S5. PFK-A kinetics in the absence and presence of ribosomes and Cam. Figure S6. LDHA kinetics in the absence and presence of ribosomes and Cam. Figure S7. Dependence of PYK-F activity on ribosomal antibiotics. Figure S8. Representative high-energy collision MS spectra. Figure S9. Possible ribosomal protein interactors. Figure S10. Distribution of intracellular and extracellular metabolites. Figure S11. Extracellular [U–^13^C_6_]-glucose flux profiles. Figure S12. Flow-through, FT, of ^13^C-Glc_ex_, Cam, and unidentified metabolite X. Figure S13. Intracellular ^13^C-lactate flux profiles. Figure S14. Intracellular ^13^C-formate flux profiles. Figure S15. Intracellular ^13^C-ethanol flux profiles. Figure S16. Intracellular ^13^C-acetate flux profiles. Figure S17. Intracellular ^13^C-alanine flux profiles. Figure S18. Intracellular ^13^C-valine flux profiles. Figure S19. Intracellular ^13^C-glutamate-γ flux profiles. Figure S20. Intracellular ^13^C-succinate flux profiles. Table S1. Steady-state kinetic parameters resolved for PFK-A. Table S2. Steady-state kinetic parameters resolved for LDH-A. Table S3. Possible intermolecular cross-links between PYK-F and ribosome proteins. Table S4. Glycolytic enzymes interacting with ribosomal proteins or ribosome-associated factors. Table S5. Glc_ex_ best-fit parameters. Table S6. Lactate flux profile best fit parameters. Table S7. Formate flux profile best fit parameters. Table S8. Ethanol flux profile best fit parameters. Table S9. Acetate flux profile best fit parameters. Table S10. Alanine flux profile best fit parameters. Table S11. Valine flux profile best fit parameters. Table S12. Glu-γ flux profile best fit parameters. Table S13. Succinate flux profile best fit parameters (PDF)
