# Supplementary material for: In Vivo Ribosome-Amplified MetaBOlism, RAMBO, Effect Observed by Real Time Pulse Chase, RTPC, NMR Spectroscopy
Source: Biochemistry. 2025 May 27;64(12):2660–78. doi: 10.1021/acs.biochem.5c00086 (PMC12177922; doi:10.1021/acs.biochem.5c00086)
Supplement: Supplementary file 1 [file bi5c00086_si_001.pdf]

Supporting Information

In vivo Ribosome-Amplified MetaBOlism, RAMBO, effect observed by real time pulse chase, RTPC, NMR Spectroscopy

*Jianchao Yu[1], Nicholas Sciolino[1], Leonard Breindel[1], Qishan Lin[2], David S. Burz[1], and Alexander Shekhtman[1]**

[1]Department of Chemistry, University at Albany, State University of New York, Albany, New York, 12222, United States

[2]RNA Epitranscriptomics & Proteomics Resource, University at Albany, State University of New York, Albany, New York, 12222, United States

*Corresponding author, email: ashekhtman@albany.edu

KEYWORDS: Metabolic enzyme regulation; glycolysis; 70S ribosome; ribosomal antibiotics; chloramphenicol; chemical crosslinking coupled mass spectrometry; in-cell NMR; isotopic tracing; real-time kinetic flux profiling; pyruvate kinase; lactate dehydrogenase; phosphofructokinase; mixed acids fermentation.

Supporting Information

Figure S1. Quality Control of Cell Beads and Modified RTPC-NMR Bioreactor Setup. 3
Figure S2. Metabolite Calibration Curves. 4
Figure S3. Reduction of ATPase and Dehydrogenase Contamination in Ribosome Preparations. 5
Figure S4. PYK-F Purification. 6
Figure S5. PFK-A Kinetics in the Absence and Presence of Ribosomes and Cam. 7
Figure S6. LDHA Kinetics in the Absence and Presence of Ribosomes and Cam. 8
Figure S7. Dependence of PYK-F Activity on Ribosomal Antibiotics. 9
Figure S8. Representative High-energy Collision MS Spectra. 10
Figure S9. Possible Ribosomal Protein Interactors. 11
Figure S10. Distribution of Intracellular and Extracellular Metabolites. 12
Figure S11. Extracellular [*U*-$^{13}C_6$]-glucose Flux Profiles. 13
Figure S12. Flow-through, FT, of ^{13}C-Glc_{ex}, Cam, and Unidentified Metabolite X. 14
Figure S13. Intracellular ^{13}C-lactate Flux Profiles. 15
Figure S14. Intracellular ^{13}C-formate Flux Profiles. 16
Figure S15. Intracellular ^{13}C-ethanol Flux Profiles. 17
Figure S16. Intracellular ^{13}C-acetate Flux Profiles. 18
Figure S17. Intracellular ^{13}C-alanine Flux Profiles. 19
Figure S18. Intracellular ^{13}C-valine Flux Profiles. 20
Figure S19. Intracellular ^{13}C-glutamate-γ Flux Profiles. 21
Figure S20. Intracellular ^{13}C-succinate Flux Profiles. 22

Table S1. Steady-state kinetic parameters resolved for PFK-A. 23
Table S2. Steady-state kinetic parameters resolved for LDH-A. 24
Table S3. Possible intermolecular crosslinks between PYK-F and ribosome proteins. 25
Table S4. Glycolytic enzymes interacting with ribosomal proteins or ribosome-associated factors. 27
Table S5. Glc_{ex} Best-fit Parameters. 29
Table S6. Lactate flux profile best fit parameters. 30
Table S7. Formate flux profile best fit parameters. 31
Table S8. Ethanol flux profile best fit parameters. 32
Table S9. Acetate flux profile best fit parameters. 33
Table S10. Alanine flux profile best fit parameters. 34
Table S11. Valine flux profile best fit parameters. 35
Table S12. Glu-γ flux profile best fit parameters. 36
Table S13. Succinate flux profile best fit parameters. 37
References. 38

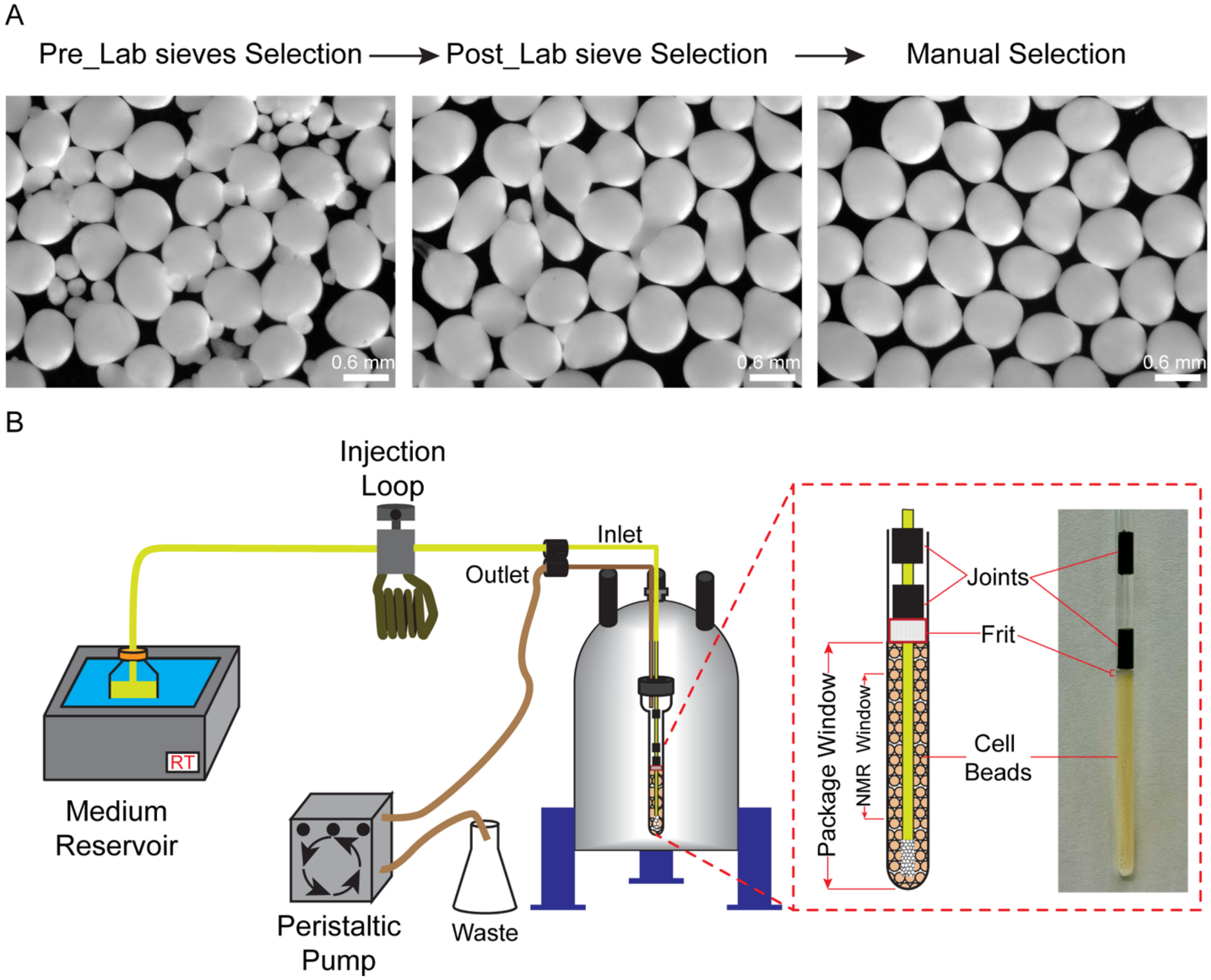

Figure S1. Quality Control of Cell Beads and Modified RTPC-NMR Bioreactor Setup.

(A) Uniform cell bead size was achieved by selection using lab sieves and manual picking. Only beads with a diameter of 0.91 ± 0.017 mm were loaded into the NMR tube. (B) Basic RTPC-NMR platform setup. A hydrophilic column frit (highlighted in the red mesh block) was introduced for immobilizing package cell beads. The resulting package window (~5.5 cm) is sufficiently wide to fully cover the NMR detection window (~2 cm), ensuring optimal signal capture during experiments.

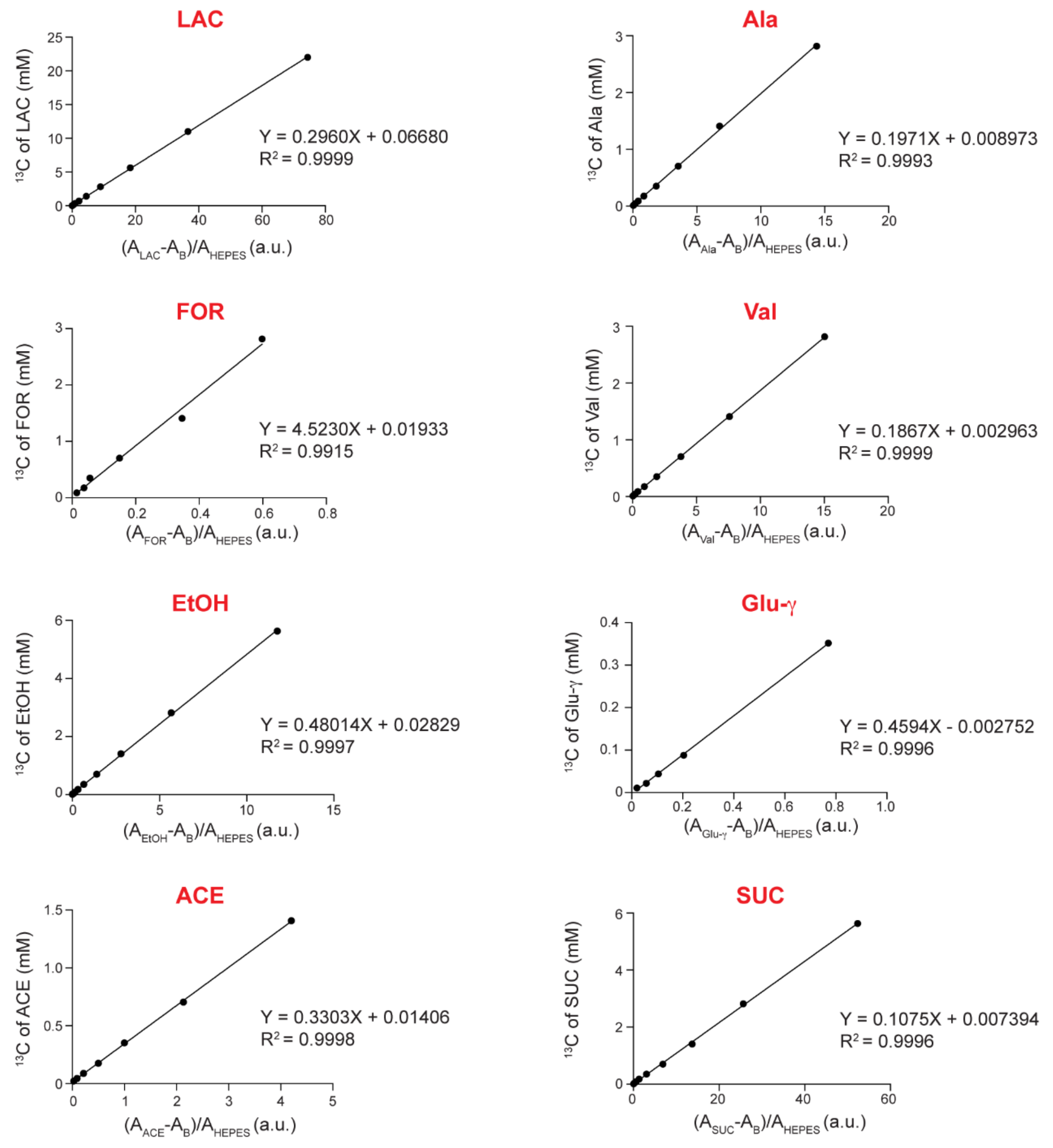

Figure S2. Metabolite Calibration Curves.

Linear regression of the natural ^{13}C abundance of in vitro samples were performed to calculate molar concentrations of metabolites. All calculations were performed using GraphPad Prism 9.

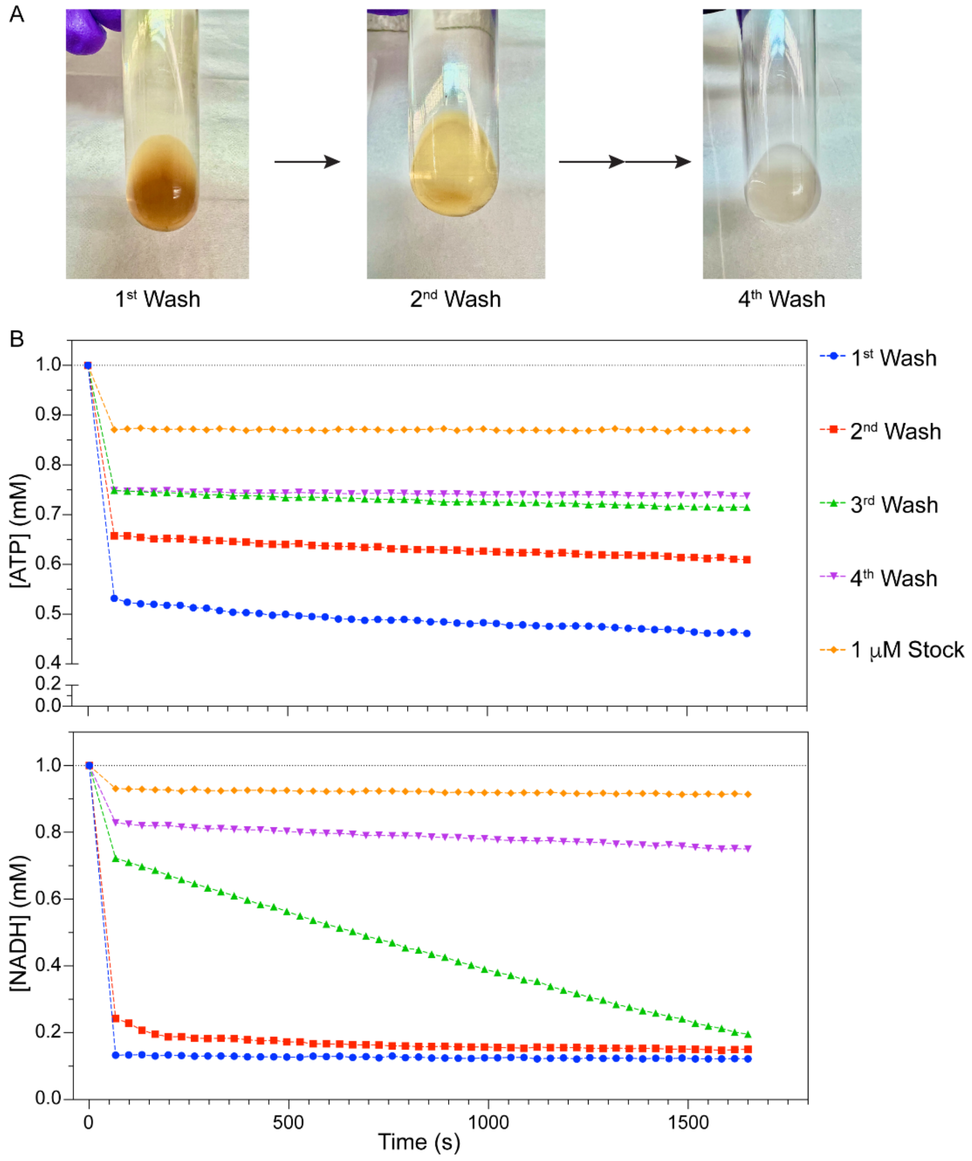

Figure S3. Reduction of ATPase and Dehydrogenase Contamination in Ribosome Preparations.

(A) Visual monitoring of the ribosome pellet during high-salt washing. (B) Quantitative assessment of residual ATPase and dehydrogenase contamination using a proton NMR-based direct assay. Initial concentrations of 1 mM ATP (top) and NADH (bottom), respectively, were incubated with 1 μM of intact ribosome solution at 298K for ~ 30 min, corresponding to the enzyme activity assay duration.

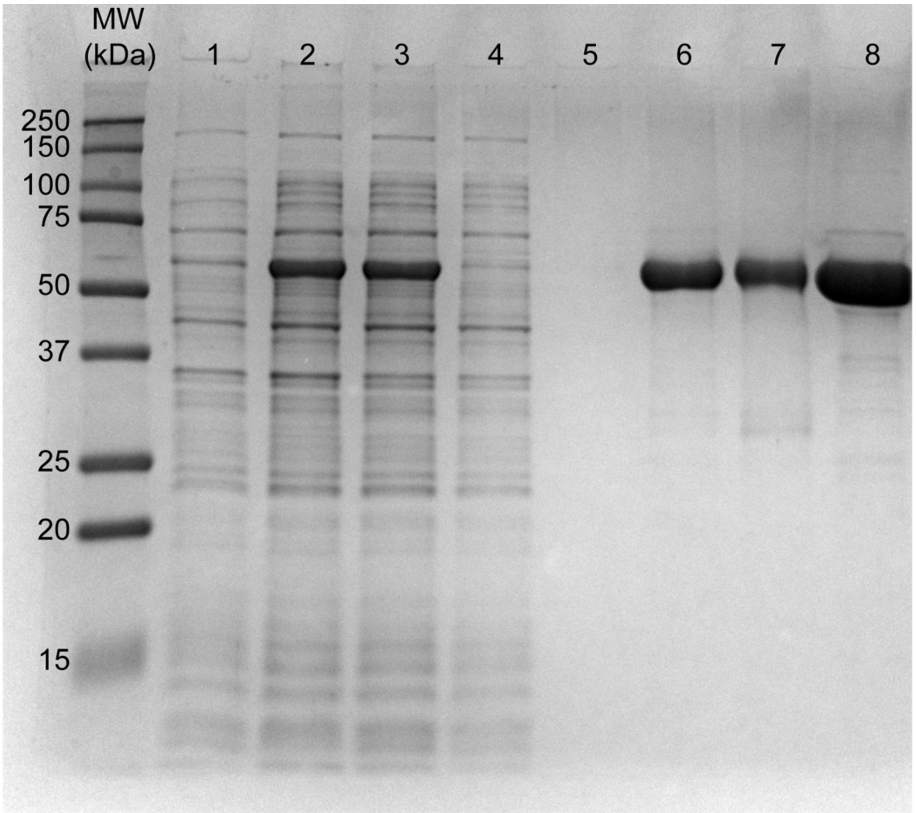

Figure S4. PYK-F Purification.

MW: molecular weight markers. Lane 1: whole cell lysate before IPTG induction. Lane 2: whole cell lysate ~6 h post-induction. Lane 3: whole cell lysate supernatant. Lane 4: Ni-NTA batch column flow through. Lane 5: last wash step. Lane 6: 6th elution fraction. Lane 7: 7th elution fraction. Lane 8: Final product ~2 μM.

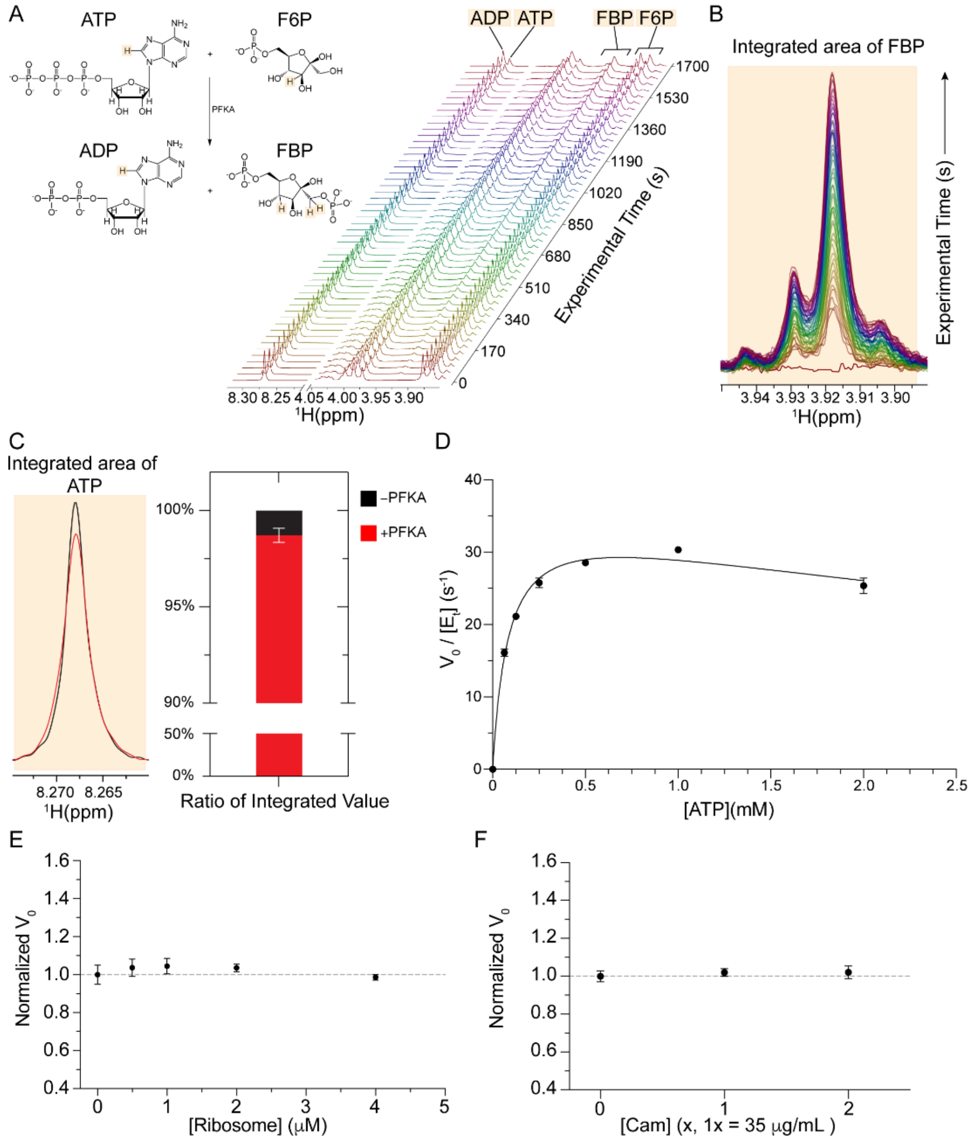

Figure S5. PFK-A Kinetics in the Absence and Presence of Ribosomes and Cam.

(A) Left, the reaction catalyzed by PFK-A. Right, pseudo-2D ^{1}H NMR stacked spectra showing the proton signals of reactants and products from the PFK-A reaction. (B) Spectra were analyzed by superimposing and integrating the proton peak of FBP and converting the values to concentrations. (C) Superimposed H6 proton peaks of 1 mM ATP integrated over the same range before (black) and after (red) the addition of PFK-A. The ratios of integrated values show a loss of <10% ATP during the reaction dead time. (D) ATP saturation curve at 1 mM F6P. The curve was fit using the Michaelis-Menten equation modified to include a binding site for substrate inhibition, $V_0 = V_{max}[S]/(K_M + [S](1+[S]/K_I))$. (E) PFK-A activity is unaffected by ribosomes. (F) PFK-A activity is unaffected by Cam. All error bars represent means ± SEM from three independent trials.

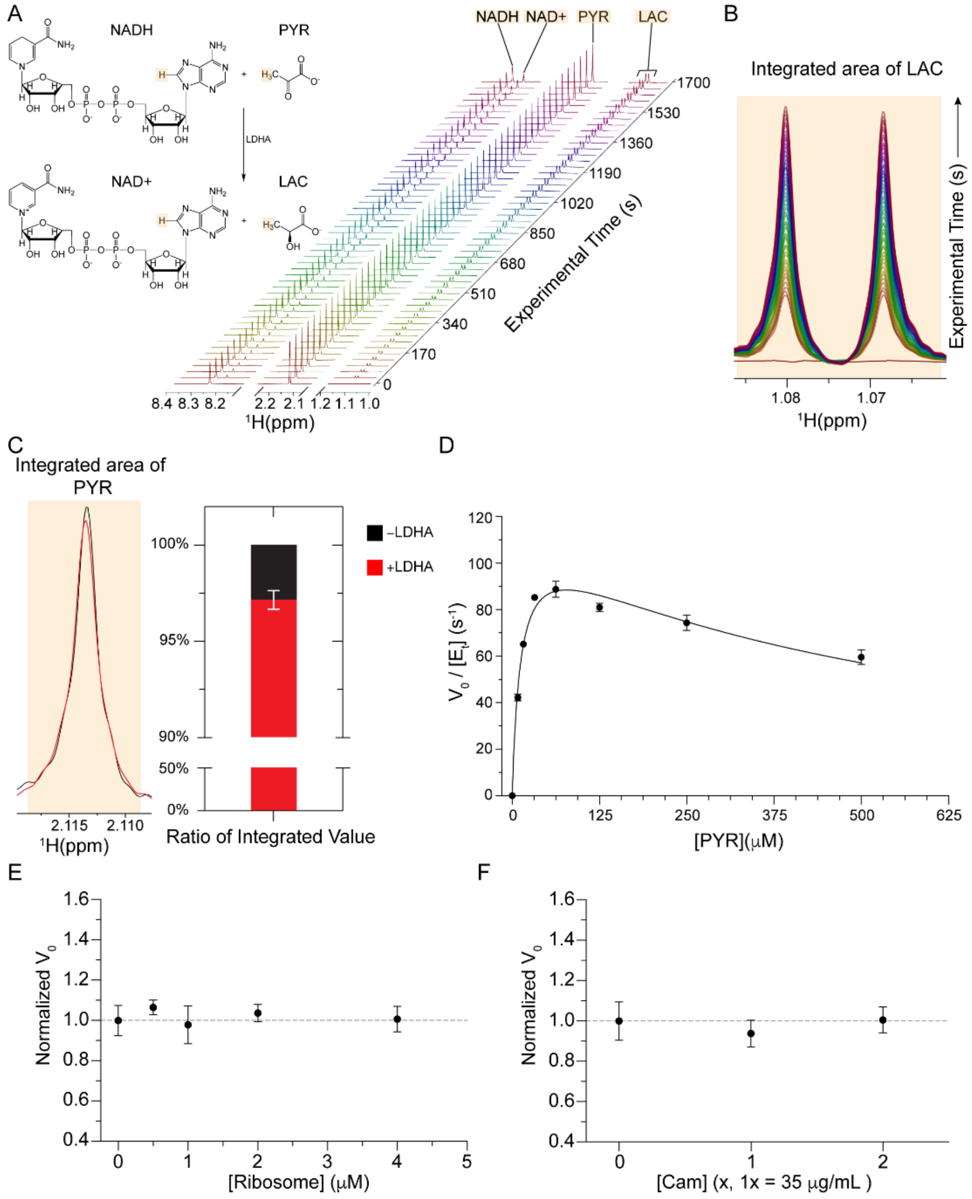

Figure S6. LDHA Kinetics in the Absence and Presence of Ribosomes and Cam.

(A) Left, the reaction catalyzed by LDH-A. Right, pseudo-2D ^{1}H NMR stacked spectra showing the proton signals of reactants and products from the LDH-A reaction. (B) Spectra were analyzed by superimposing and integrating the methyl proton peak of LAC and converting the values to concentrations. (C) Superimposed methyl proton peaks of 1 mM PYR integrated over the same range before (black) and after (red) the addition of LDH-A. The ratios of integrated values show a loss of <10% PYR during the reaction dead time. (D) PYR saturation curve at 0.5 mM NADH. The curve was fit using the Michaelis-Menten equation modified to include a binding site for substrate inhibition, $V_0 = V_{max}[S]/(K_M + [S](1+[S]/K_I))$. (E) LDH-A activity is unaffected by ribosomes. (F) LDH-A activity remains unaffected by chloramphenicol. All error bars represent means ± SEM from three independent trials.

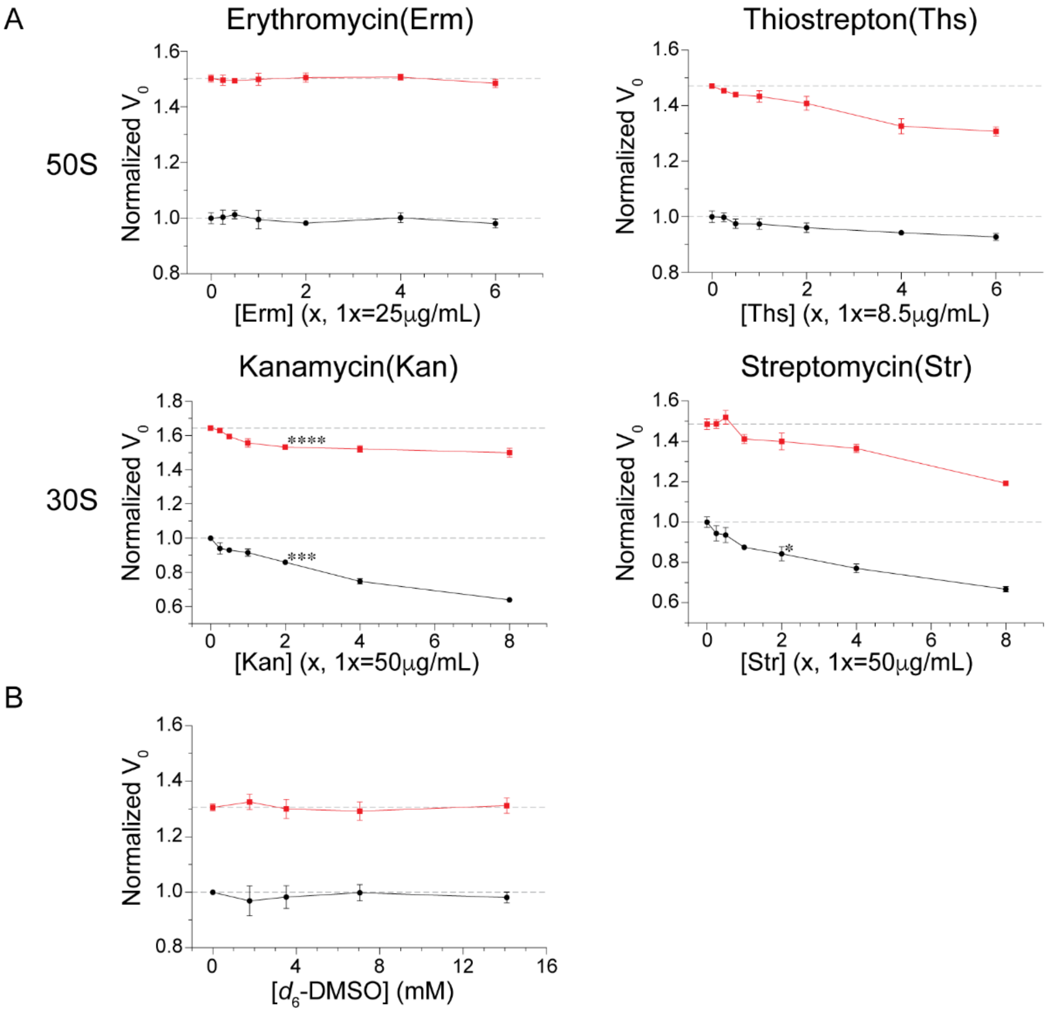

Figure S7. Dependence of PYK-F Activity on Ribosomal Antibiotics.

PYK-F activity measured for free (black) and ribosome-bound (red) enzyme: (A) Top, Erythromycin and thiostrepton, bind to the 50S subunit of the ribosome. Bottom, Kanamycin and streptomycin bind to the 30S subunit of the ribosome. The concentrations of ADP and PEP were 4 and 2 mM, respectively. All initial velocities, V_0, were normalized to the initial velocity of PYK-F in the absence of intact ribosomes and ribosomal antibiotics. The statistical significance of the normalized velocities, V_0, at 2× antibiotic concentration is indicated where applicable. (*, p <0.05 and d >1.0; ***, p <0.001 and d >1.0; ****, p <0.0001 and d >1.0). (B) Dependence of PYK-F activity on d^6-DMSO in the presence (red) and absence (black) of 3 μM ribosomes. All error bars represent means ± SEM from three independent trials.

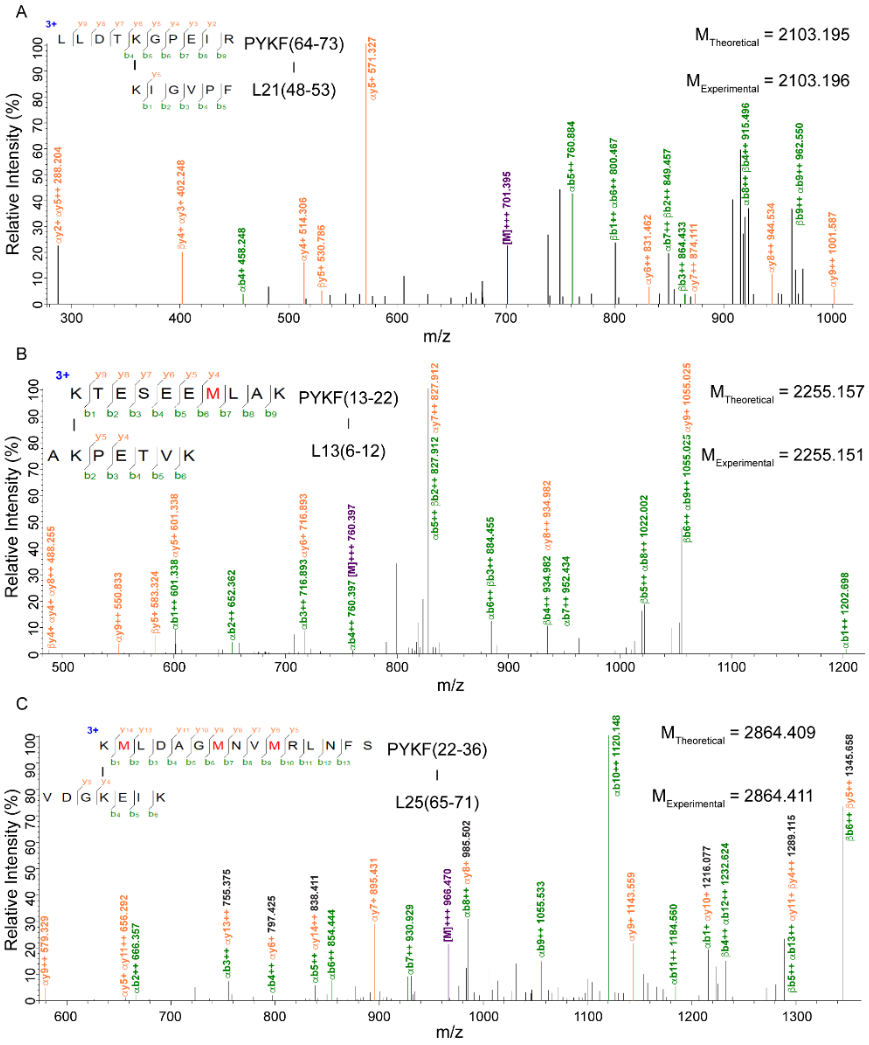

Figure S8. Representative High-energy Collision MS Spectra.

(A) Cross-linked peptide between ribosomal protein L21 and PYK-F is detected at m/z 701.395 (purple). The experimental mass ($M_{Experimental}$) of this triple charged precursor ion, 2103.196, matched the theoretical mass ($M_{Theoretical}$) of 2103.195. Image was prepared using pLabel with 15 Da tolerance. (B) Cross-linked peptide between ribosomal protein L13 and PYK-F is seen at m/z 760.397 (purple). The experimental mass ($M_{Experimental}$) of this triple charged precursor ion, 2255.151, matched the theoretical mass ($M_{Theoretical}$) of 2255.157. Image was prepared using pLabel with 25 Da tolerance. (C) Cross-linked peptide between ribosomal protein L25 and PYK-F is detected at m/z 966.470 (purple). The experimental mass ($M_{Experimental}$) of this triple charged precursor ion, 2864.411, matched the theoretical mass ($M_{Theoretical}$) of 2864.409. Image was prepared using pLabel with 20 Da tolerance. In all images: peptide sequences with cross-linked lysines are shown (top left). The b fragmentation ions (green) that extend from the amino terminus and y fragmentation ions (orange) that extend from the carboxyl terminus are labeled.

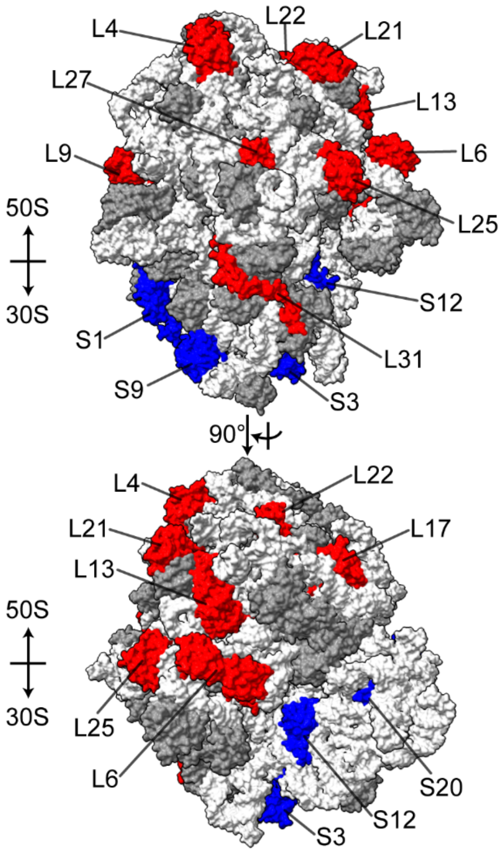

Figure S9. Possible Ribosomal Protein Interactors.

Surface model of intact ribosome (PDB entry 6BU8[1]) with rRNA in light grey and ribosomal proteins in dark grey. Potential crosslinked RPs L4, L6, L9, L13, L17, L21, L22, L25, L27 and L31 from the 50S subunit are in red and RPs S1, S3, S9, S12 and S20 from the 30S subunit are in blue.

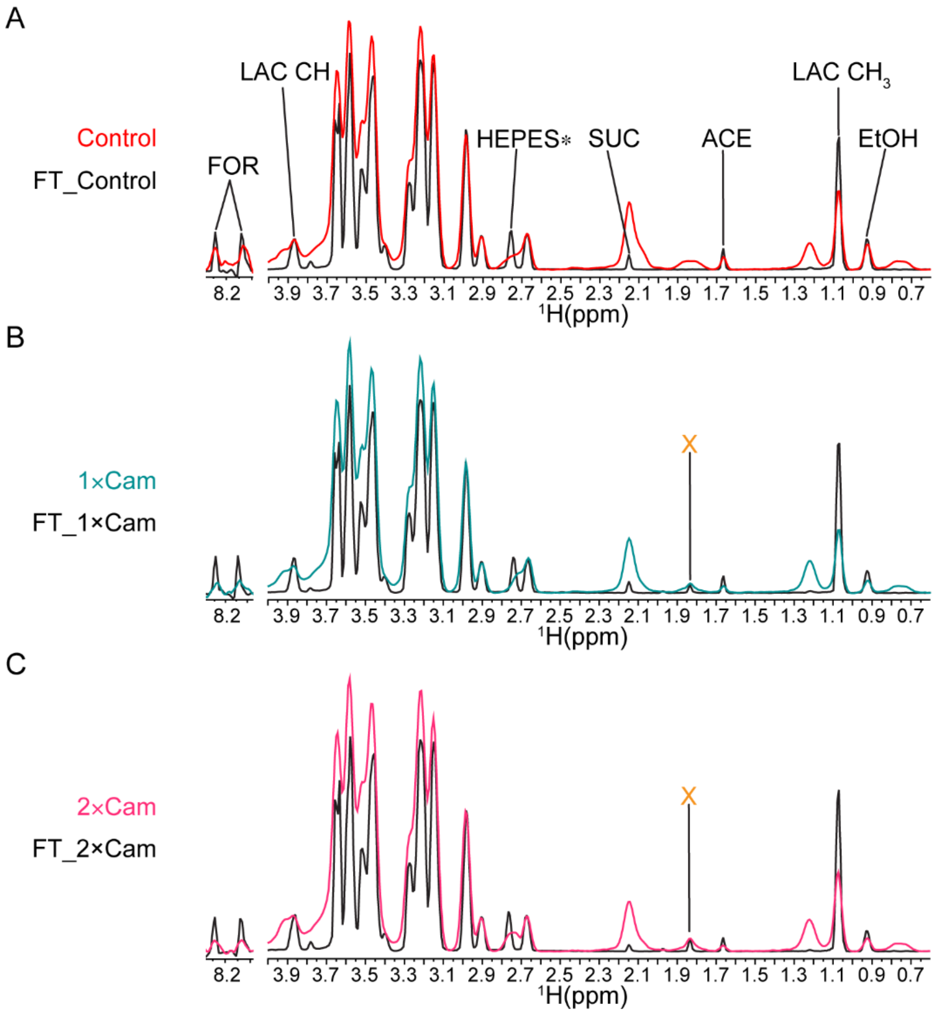

Figure S10. Distribution of Intracellular and Extracellular Metabolites.
Overlay of intracellular (black) and extracellular (colored) metabolite 1D ^{13}C-edited proton HSQC spectra at the mid-point of the ^{13}C-glucose pulse window under (A) control, (B) 1×Cam, and (C) 2×Cam conditions. Intracellular spectra were scaled to the HEPES peak at 2.9 ppm relative to the extracellular spectra before adjusting to γ-NTP levels. The peak at 2.75 ppm, HEPES*, arises from the natural ^{13}C abundance but is shifted in the presence of Cam due to the presence of various fermentation acids in the FT fraction.

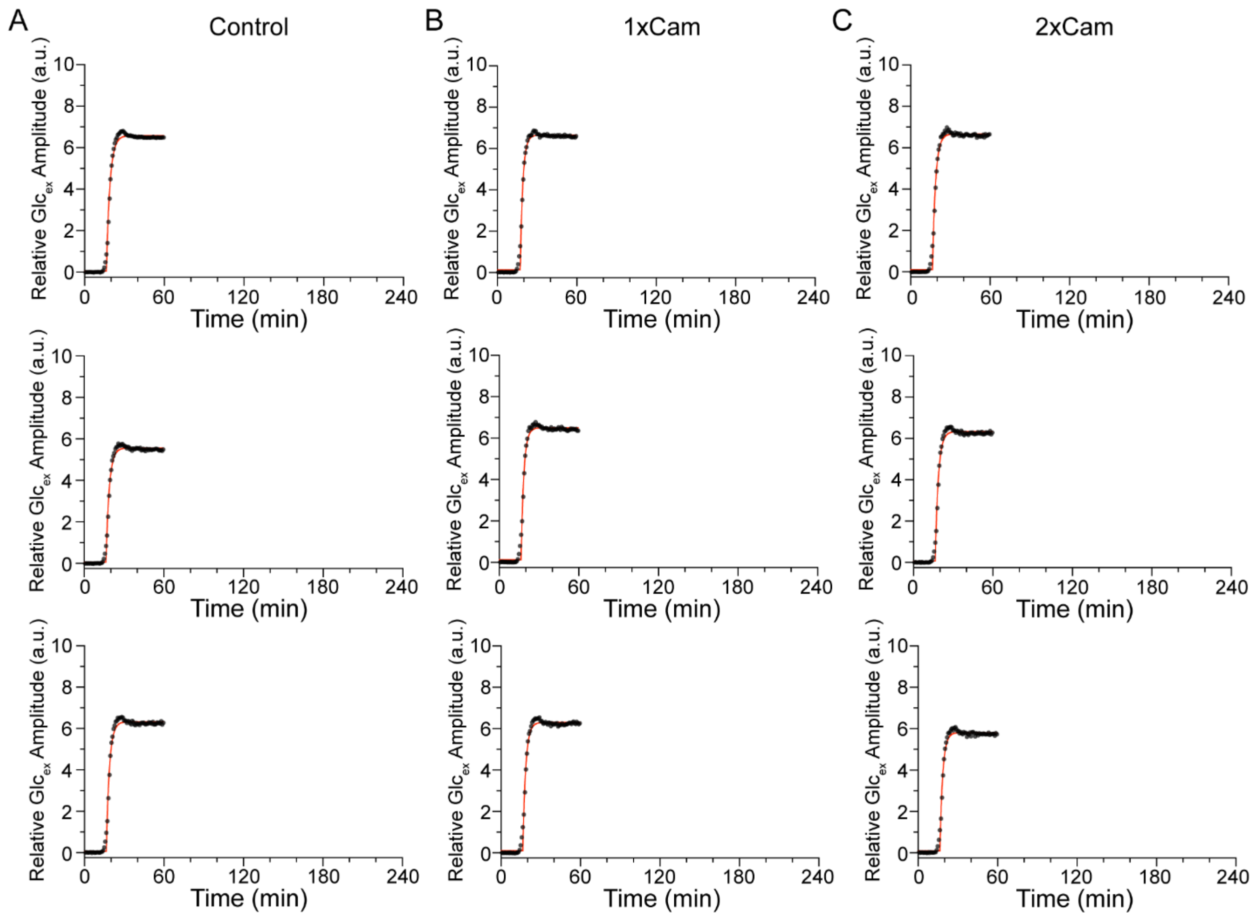

Figure S11. Extracellular [U-$^{13}C_6$]-glucose Flux Profiles.

The leading edges of the glucose flux profiles were fit to a one phase association model (**Eq. 4**) using GraphPad Prism 9. (A) control; (B) 1×Cam and (C) 2×Cam. Best-fit parameters are summarized in **Table S5**.

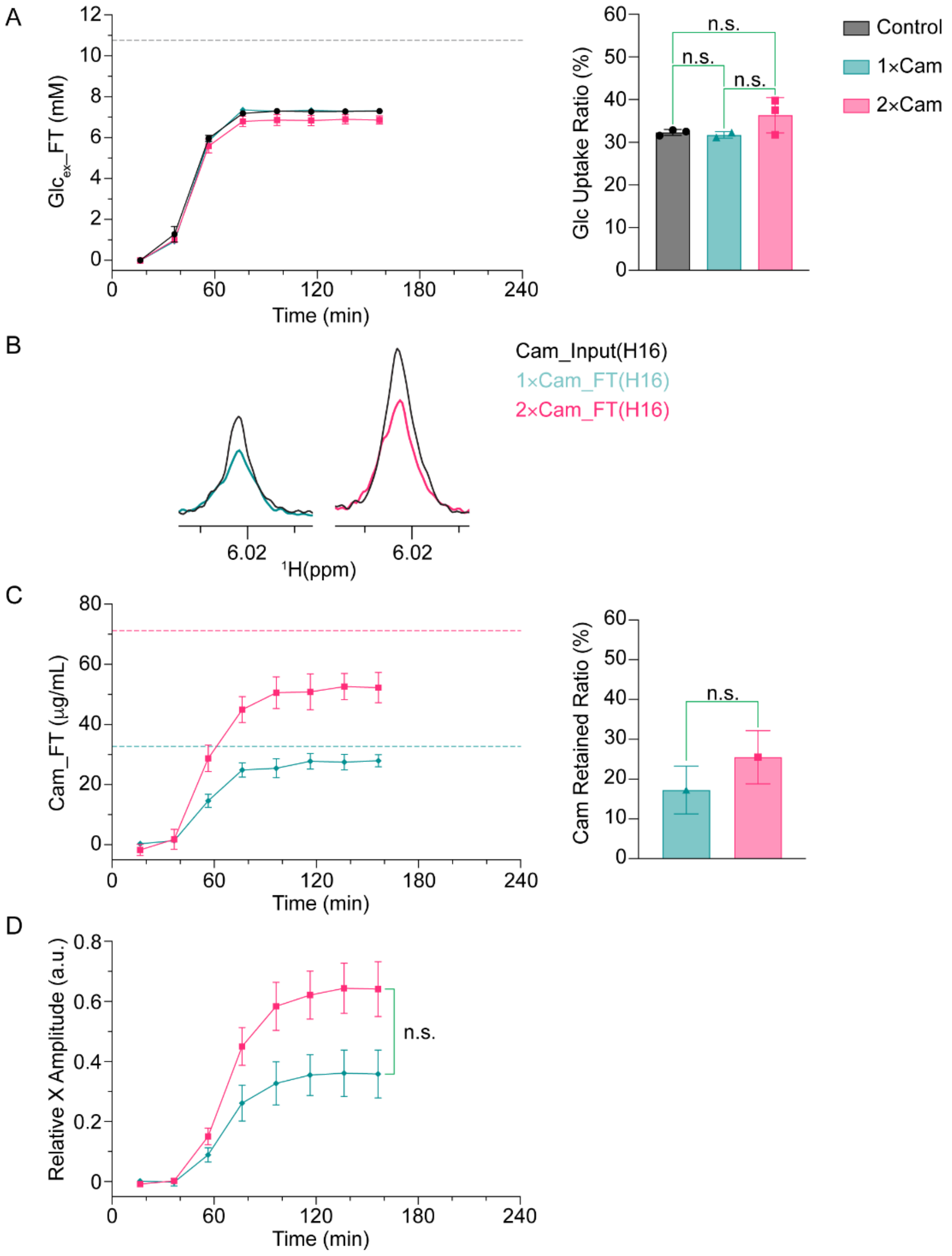

Figure S12. Flow-through, FT, of ^{13}C-Glc$_{ex}$, Cam, and Unidentified Metabolite X.
(A) Left, time-course of ^{13}C-Glc$_{ex}$ FT fractions. Right, glucose uptake of cells with and without Cam treatments. (B) Comparison of intensity of Cam proton H16 peak between Cam input and FT. (C) Left, concentration of Cam in FT over time. Right, % of Cam retained. (D) Peak amplitude of unidentified metabolite X in FT over time. All error bars represent means ± SEM from three independent trials. n.s., nonsignificant.

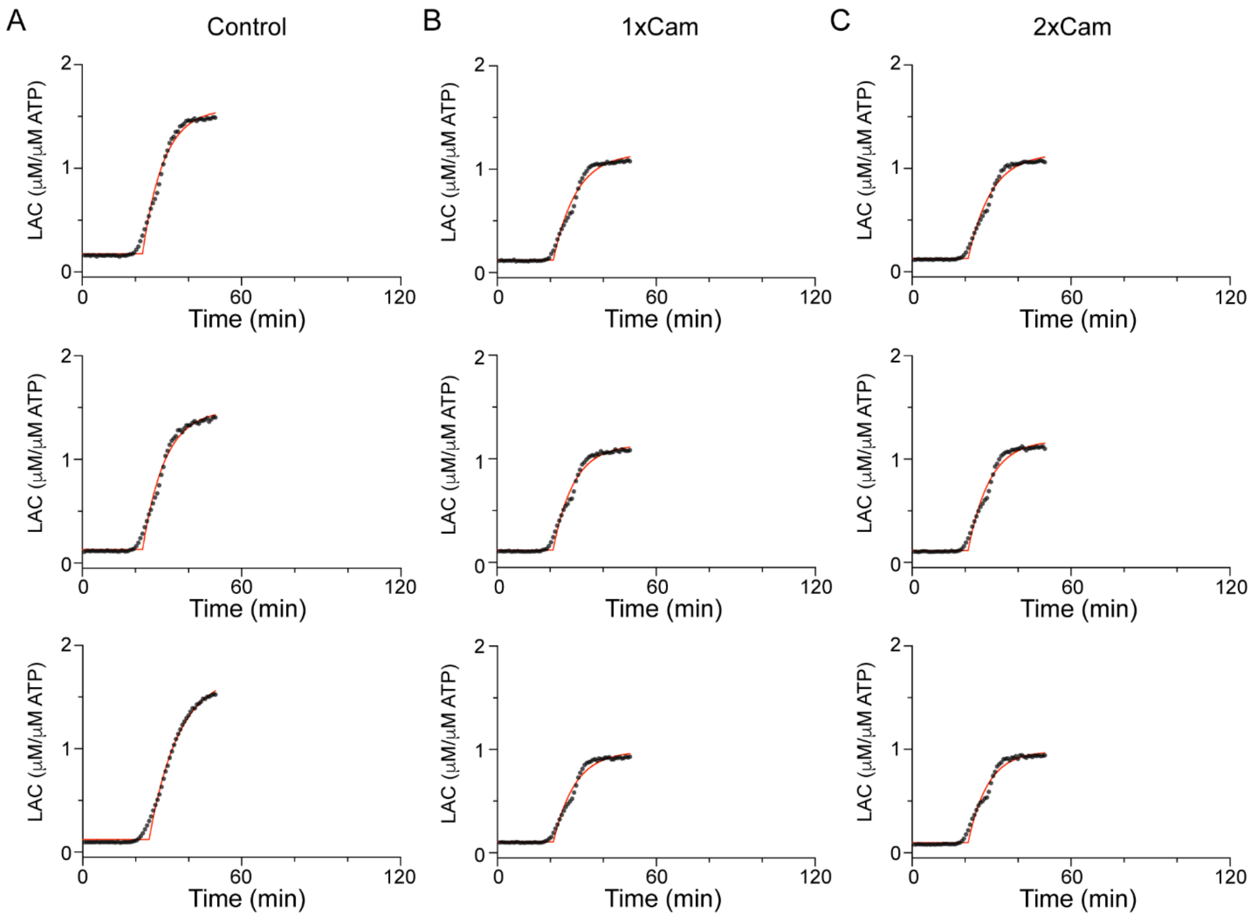

Figure S13. Intracellular ^{13}C-lactate Flux Profiles.

The leading edges (black) of the lactate flux profiles were fit (red) to a one phase association model (**Eq. 4**) using GraphPad Prism 9. (A) control; (B) 1×Cam and (C) 2×Cam. Best-fit parameters are summarized in **Table S6**.

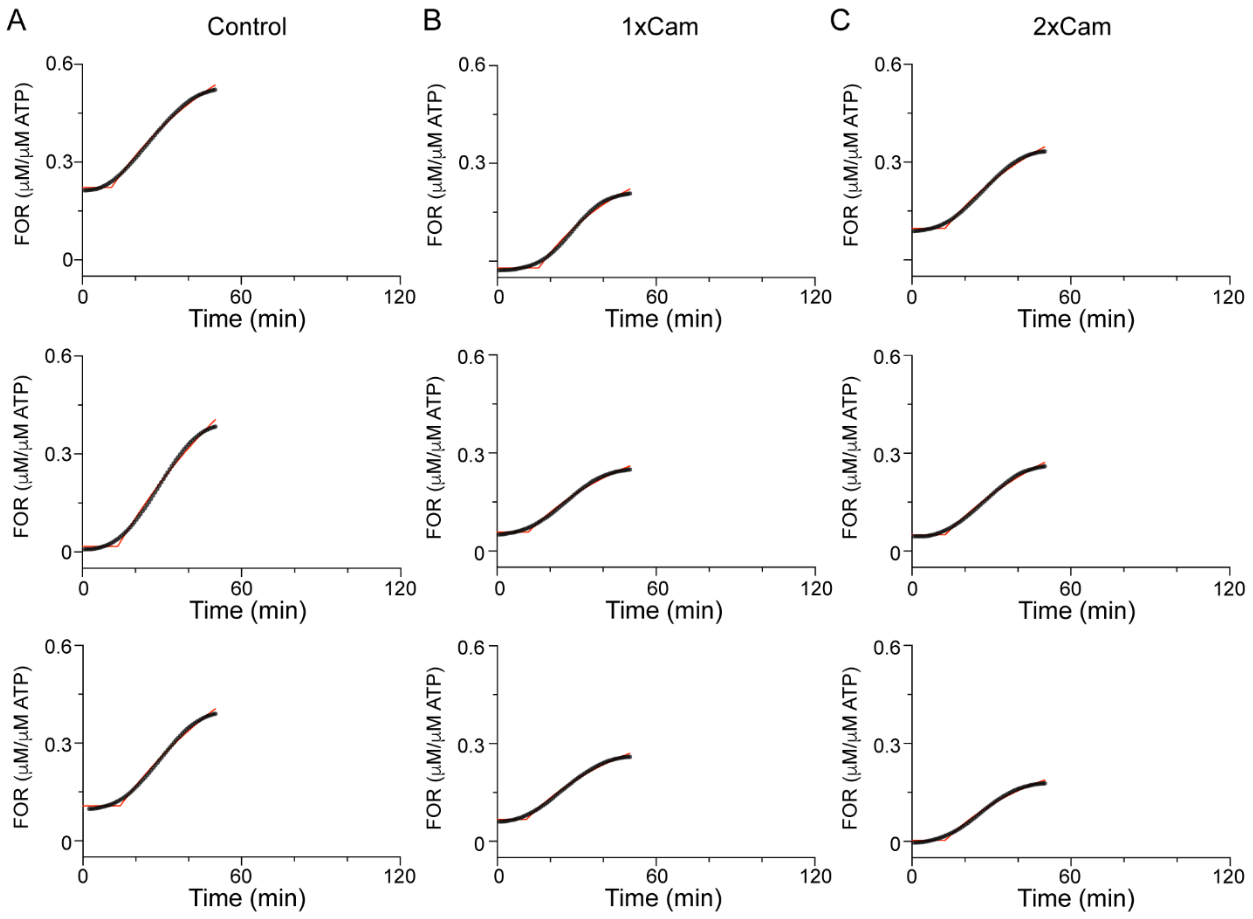

Figure S14. Intracellular ^{13}C-formate Flux Profiles.

The leading edges (black) of the formate flux profiles were fit (red) to a one phase association model (**Eq. 4**) using GraphPad Prism 9. (A) control; (B) 1×Cam and (C) 2×Cam. Best-fit parameters are summarized in **Table S7**.

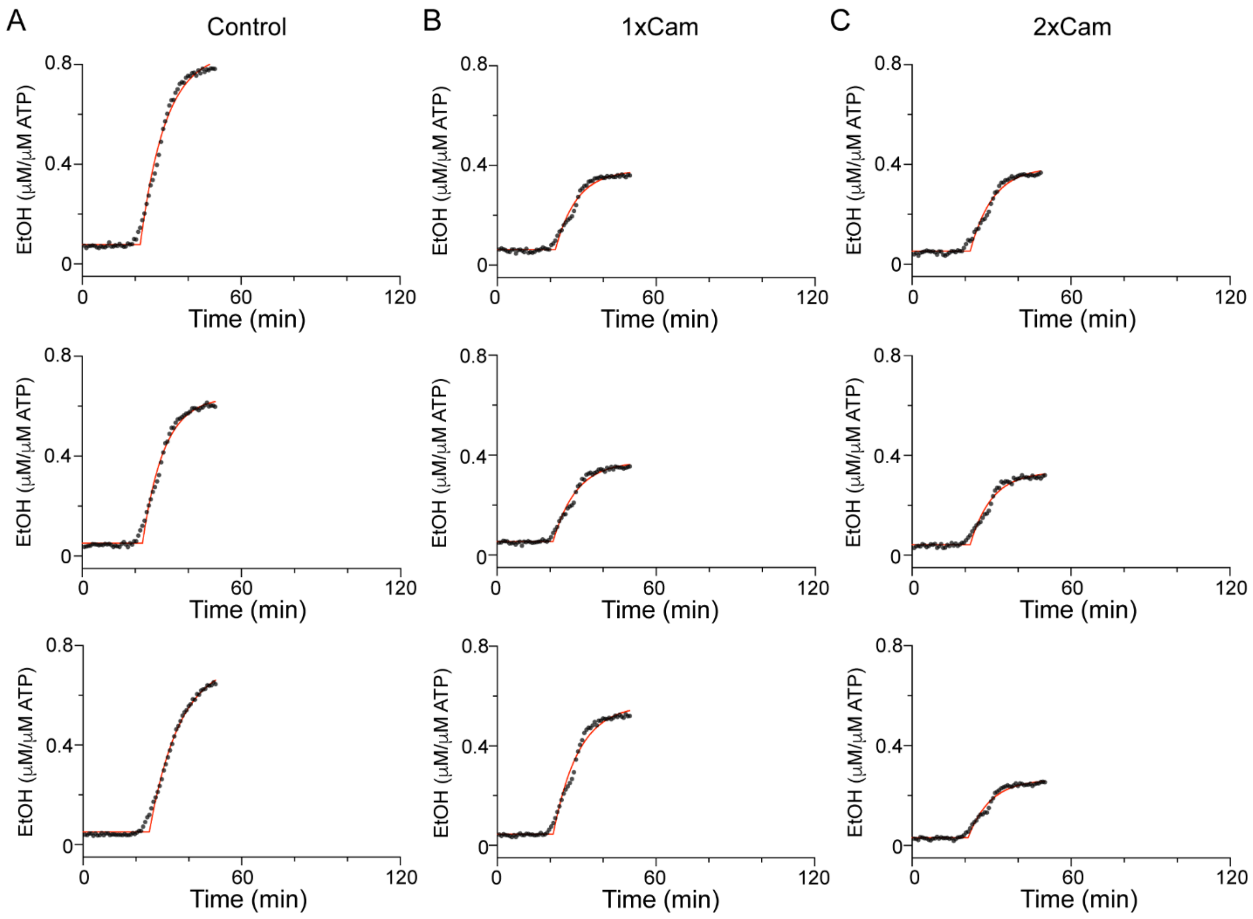

Figure S15. Intracellular ^{13}C-ethanol Flux Profiles.

The leading edges (black) of the ethanol flux profiles were fit (red) to a one phase association model (**Eq. 4**) using GraphPad Prism 9. (A) control; (B) 1×Cam and (C) 2×Cam. Best-fit parameters are summarized in **Table S8**.

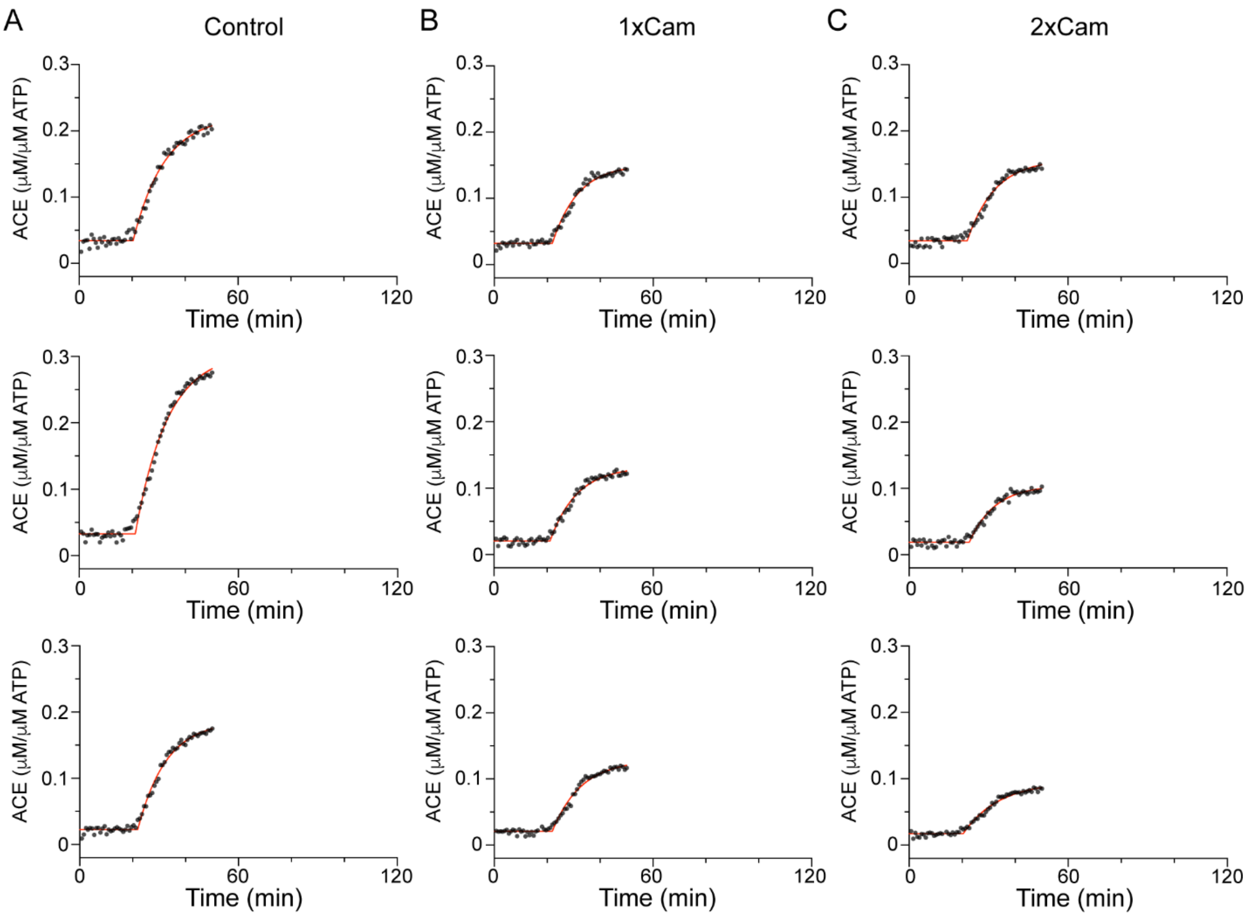

Figure S16. Intracellular ^{13}C-acetate Flux Profiles.

The leading edges (black) of the acetate flux profiles were fit (red) to a one phase association model **(Eq. 4)** using GraphPad Prism 9. (A) control; (B) 1×Cam and (C) 2×Cam. Best-fit parameters are summarized in **Table S9**.

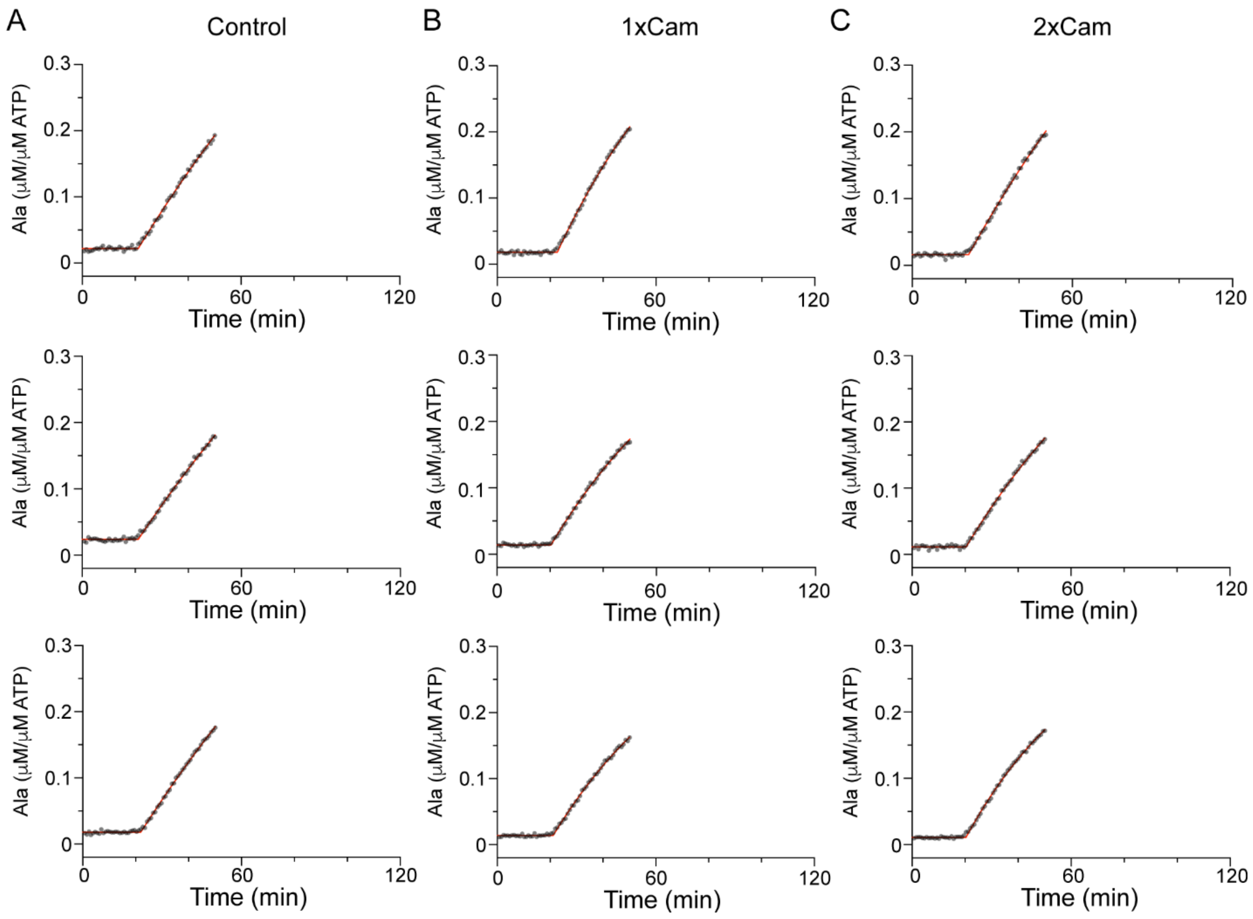

Figure S17. Intracellular ^{13}C-alanine Flux Profiles.

The leading edges (black) of the alanine flux profiles were fit (red) to a one phase association model (**Eq. 4**) using GraphPad Prism 9. (A) control; (B) 1×Cam and (C) 2×Cam. Best-fit parameters are summarized in **Table S10**.

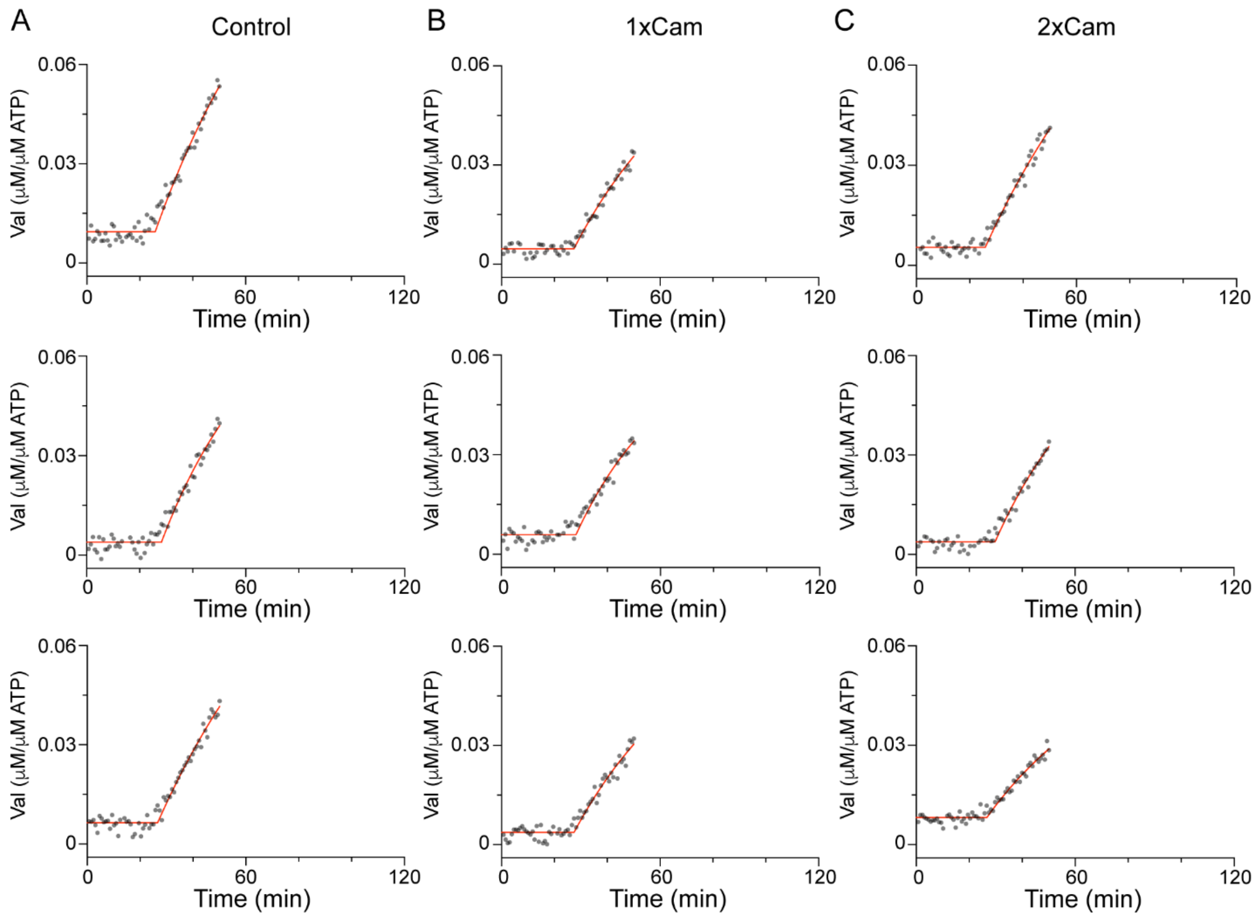

Figure S18. Intracellular ^{13}C-valine Flux Profiles.

The leading edges (black) of the valine flux profiles were fit (red) to a one phase association model (**Eq. 4**) using GraphPad Prism 9. (A) control; (B) 1×Cam and (C) 2×Cam. Best-fit parameters are summarized in **Table S11**.

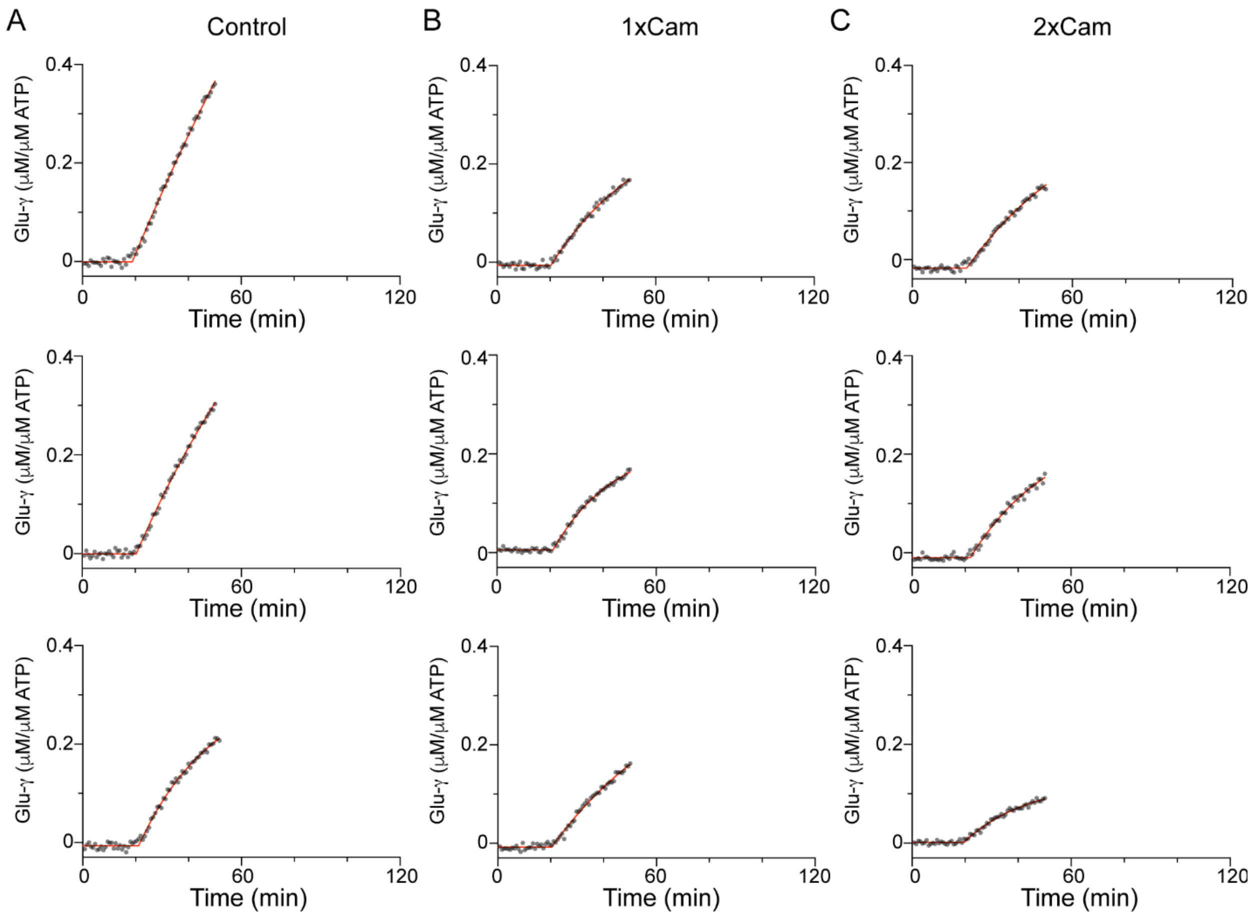

Figure S19. Intracellular ^{13}C-glutamate-γ Flux Profiles.

The leading edges (black) of the glutamate flux profiles were fit (red) to a one phase association model (**Eq. 4**) using GraphPad Prism 9. (A) control; (B) 1×Cam and (C) 2×Cam. Best-fit parameters are summarized in **Table S12**.

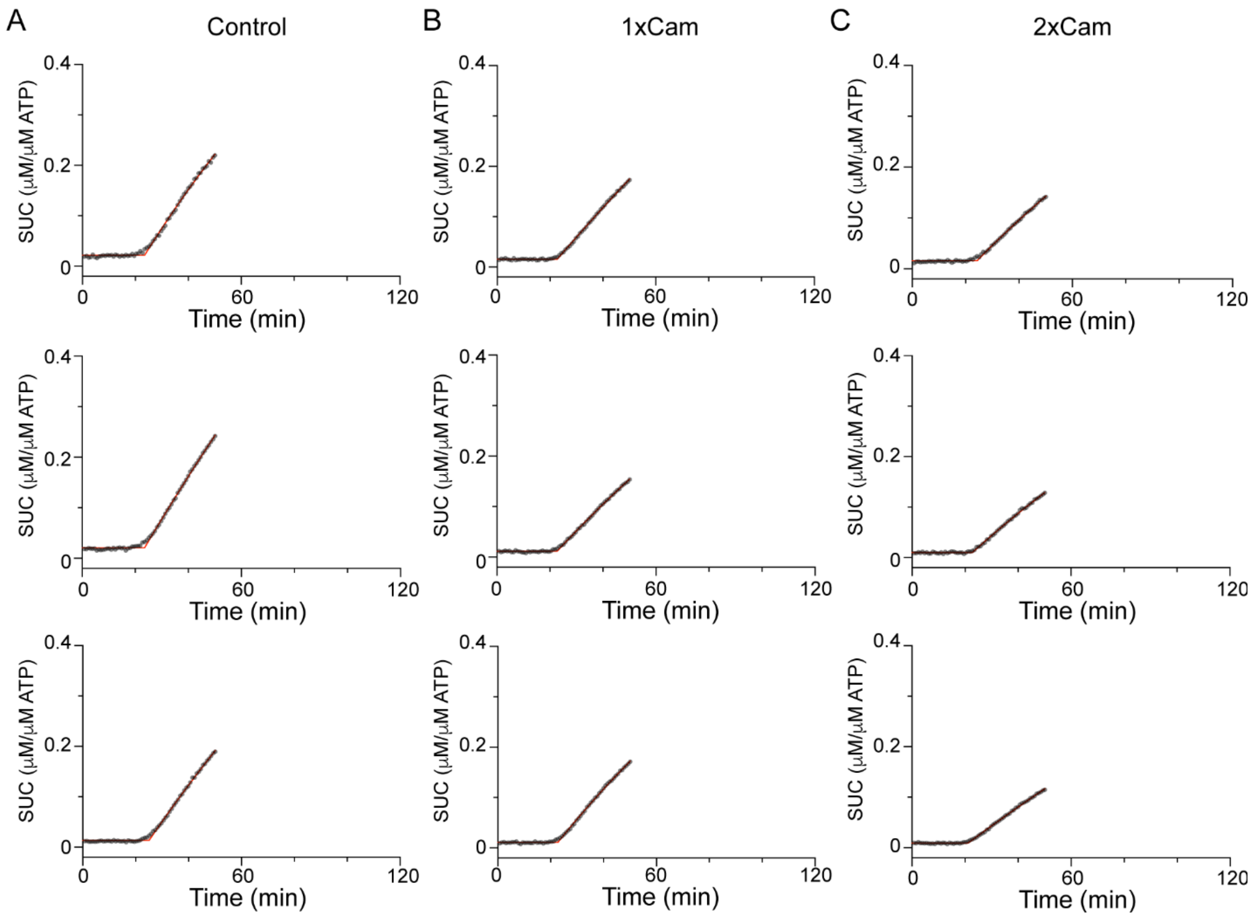

Figure S20. Intracellular ^{13}C-succinate Flux Profiles.
The leading edges (black) of the succinate flux profiles were fit (red) to a one phase association model (**Eq. 4**) using GraphPad Prism 9. (A) control; (B) 1×Cam and (C) 2×Cam. Best-fit parameters are summarized in **Table S13**.

Table S1. Steady-state Kinetic Parameters Resolved for PFK-A.

Substrate	K_M (μM)	k_{cat} (s^{-1})	K_I (mM)	R^2
ATP [a]	84 ± 9.8	36 ± 1.5	5.6 ± 1.2	0.99
ATP [b]	60	0.05 [c]	N/A [d]	N/A
ATP [e]	70 ± 10	100 [f]	N/A [g]	0.996

[a] This work, *B. stearothermophilus* PFK-A, pH 7.5, [F6P] were fixed at 1 mM, NMR-based direct assay at 10 °C.
[b] *E. coli* PFK-A, pH 8.5 coupled assay at 28 °C.[2]
[c] Units are OD/min.
[d] K_I was not cited and substrate inhibition was not observed because the Mg^{2+}/ATP ratio was kept constant at 10:1.
[e] *B. stearothermophilus* PFK-A, pH 8.2, [F6P] were fixed at 1 mM, coupled assay at 30 °C.[3]
[f] Units are % of k_{cat} (ATP).
[g] K_I was not provided but substrate inhibition was observed.

Table S2. Steady-state Kinetic Parameters Resolved for LDH-A.

Substrate	K_M (μM)	k_{cat} (s^{-1})	K_I (μM)	R^2
Pyruvate [a]	12 ± 1.6	116 ± 5.5	495 ± 74.0	0.98
Pyruvate [b]	7200	286 [c]	N/A [d]	N/A
Pyruvate [e]	30	100 [f]	N/A [d]	N/A

[a] This work, LDH-A (Sigma -Aldrich), pH 7.5, [NADH] were fixed at 0.5 mM, NMR-based direct assay at 10 °C.
[b] LDH-A *E. coli*, pH 7.5, [NADH] were fixed at 0.33 mM, direct assay at 23 °C.[4]
[c] Units are mmoles NADH oxidized per min per mg of LDHA.
[d] K_I was not provided but substrate inhibition was observed.
[e] LDH-A *human*, pH 7.0, [NADH] were fixed at 0.15 mM, direct assay at 25 °C.[5]
[f] Units are % of maximal activity.

Table S3. Possible Intermolecular Crosslinks between PYK-F and Ribosome Proteins.

Sample Order	RPs	Crosslinked Sequences: PYK-RP [a]	Crosslinked Residues	$M_{theoretical}$	$M_{experimental}$	Error (ppm)	PYK Residues Solvent Accessibility [b,c]	RPs Residues Solvent Accessibility [b,d]		
								PDB entry 6BU8 [e]	PDB entry 4YBB [f]	PDB entry 5UYK [g]
XLinked_1	L4	KVVITATQMLDSMIKNP(15)-APKTK(3)	PYK(286)-L4(130)	2750.493	2750.487	-2.1	87.6%	82.2%	48.3%	42.5%
	L6 [h]	LDTKGPEIR(4)-ITIKGKN(4)	PYK(68)-L6(27)	2103.191	2103.199	4.0	46.9%	51.6%	56.1%	50.9%
	L9 [i]	KVVITATQMLDSMIKNP(15)-PATKK(4)	PYK(286)-L9(41)	2750.493	2750.487	-2.1	87.6%	88.0%	94.8%	92.7%
	S1	LDAPLIVVATQGGKS(14)-VKGIVK(2)	PYK(382)-S1(196)	2413.416	2413.413	-1.4	66.2%	N/A	N/A	N/A
	S3 [j]	MMIEKCIR(5)-GEDVEKLR(6)	PYK(266)-S3(86)	2302.133	2302.126	-3.6	36.4%	57.5%	47.9%	45.1%
XLinked_2	L13	KTESEEMLAK(1)-AKPETVK(2)	PYK(13)-L13(7)	2255.157	2255.151	-2.7	52.9%	64.6%	49.2%	43.1%
	L17	MLDSMIKN(7)-MAYIELVDRSEKAEAA(12)	PYK(286)-L17(121)	3064.477	3064.482	1.5	87.6%	N/A	100.0%	N/A
	L21 [i]	LLDTKGPEIR(5)-KIGVPF(1)	PYK(68)-L21(48)	2103.195	2103.196	0.6	46.9%	7.0%	52.5%	37.8%
	L22 [h]	LLDTKGPEIR(5)-KKVSQA(2)	PYK(68)-L22(28)	2103.191	2103.196	2.5	46.9%	48.0%	70.3%	52.1%
	L25 [i]	KMLDAGMNVMRLNFS(1)-VDGKEIK(4)	PYK(22)-L25(68)	2864.409	2864.411	0.8	51.4%	84.1%	75.9%	84.6%
	L27	KAGQTFTFTTDK(1)-DHTLFAKA(7)	PYK(86)-L27(62)	2548.282	2548.275	-2.6	74.1%	66.3%	49.5%	62.9%
	L31	TQGGKSARAV(5)-GHDLNLDVCSKC(11)	PYK(382)-L31(39)	2693.287	2693.284	-1.0	66.2%	84.1%	N/A	65.0%
	S9	LLDTKGPEIR(5)-VFIKPG(4)	PYK(68)-S9(22)	2103.195	2103.196	0.6	46.9%	54.8%	75.0%	50.1%
	S12 [i]	DVSLKAG(5)-LDCSGVKDRKQ(7)	PYK(86)-S12(108)	2239.148	2239.144	-2.0	74.1%	89.8%	92.6%	88.9%
	S20	LLDTKGPEIR(5)-SAKKRA(3)	PYK(68)-S20(8)	2103.202	2103.196	-2.8	46.9%	67.1%	58.3%	58.6%

[a] Green-labeled M residues were modified by oxidation and C residues were modified by carbamidomethylation. Bold red-lableled K residues indicate crosslinked lysines.

[b] Residues are considered solvent exposed if the ratio value is over 35% and solvent inaccessible if the ratio is less than 20%.

[c] Solvent accessibility of residues from each monomer of PYK was calculated together and input as average values.

[d] Solvent accessibility of residues from ribosomal proteins was calculated by using three published structural models of intact ribosome, PDB entry 6BU8 (aminoacyl-tRNA and mRNA-bound format with first two domains S1), PDB entry 4YBB (Free-bound format) and PDB entry 5UYK (aminoacyl-tRNA and mRNA-bound format), respectively.

[e] The structural model of intact ribosome, PDB entry 6BU8, lacks information about residue K1196 of S1 and the residue K121 of L17.

[f] The structural model of intact ribosome, PDB entry 4YBB, lacks information about ribosomal proteins S1 and L31.

[g] The structural model of intact ribosome, PDB entry 5UYK, lacks information about ribosomal proteins S1, and the residue K121 of L17.

[h] Ribosomal protein L6 and L22 were identified by searching under modified settings: nonspecific in enzyme channel and carbamidomethyl [C] as fixed modification

[i] Ribosomal proteins L9, L21, L25, and S12 were identified using modified settings: nonspecific in enzyme channel and oxidation [M] as variable modification.

[j] Additional ribosomal protein L22 was identified by pLink 2.0 using modified settings: trypsin in enzyme channel allowing up to five missed cleavages and oxidation [M] as variable modification.

Table S4. Glycolytic Enzymes Interacting with Ribosomal Proteins or Ribosome-associated Factors.

Glycolytic Enzymes	Gene Name	Uniport Entry	Interacted RPs or RFs	Gene Name	Uniport Entry	Involved Ribosome	Reference
PFK	*pfkA*	O34529	S2	rpsB	P21464	70S Ribosome of *Bacillus subtilis*	[6]
			IF-2	infB	P17889		
			EF-Tu	tuf	P33166		
			EF-Ts	tsf	P80700		
			EF-G	fusA	P80868		
			TF	tig	P80698		
GAPDH	*gapA*	P09124	L5	rplE	P12877		
PGK	*pgk*	P40924	S2	rpsB	P21464		
			L5	rplE	P12877		
ENO	*eno*	P37869	S11	rplK	P04969		
			IF-3	infC	P55872		
			EF-Tu	tuf	P33166		
			EF-Ts	tsf	P80700		
PGK	*pgk*	P0A799	L2	rplB	P60422	70S Ribosome of *E. coli*	[7]
			L9	rplI	P0A7R1		
			S6	rpsF	P02358		
			S14	rpsN	P0AG59		
			S20	rpsT	P0A7U7		
PYK-F	*pykF*	P0AD61	L1	rplA	P0A7L0		
			L2	rplB	P60422		
			L4	rplD	P60723		
			L9	rplI	P0A7R1		
			L13	rplM	P0AA10		
			L16	rplP	P0ADY7		
			L17	rplQ	P0AG44		
			L19	rplS	P0A7K6		
			L24	rplX	P60624		
			L25	rplY	P68919		
			S13	rpsM	P0A7S9		
			S19	rpsS	P0A7U3		
			TF	tig	P0A850		
PYK	*pyk*	Q02499	L1	rplA	P0A7L0		[8]
			L13	rplM	P0AA10		
			L25	rplY	P68919		
			S2	rpsB	P0A7V0		
			S3	rpsC	P0A7V3		

			S4	rpsD	P0A7V8		
			S13	rpsM	P0A7S9		
			S19	rpsS	P0A7U3		
TPI	*TPI1*	P00940	L11	rplK	P0A7J7		[9]

Table S5. Glc_{ex} Best-fit Parameters.

	Control_1	Control_2	Control_3	1×Cam_1	1×Cam_2	1×Cam_3	2×Cam_1	2×Cam_2	2×Cam_3
T_0 (min) [a]	= 16.45	= 16.45	= 16.45	= 17.23	= 16.45	= 16.45	= 16.45	= 16.45	= 16.45
Y_0 (a. u.) [b]	0.058 ± 0.0354	0.052 ± 0.0395	0.073 ± 0.0408	0.130 ± 0.0395	0.131 ± 0.0451	0.105 ± 0.0436	0.106 ± 0.0438	0.095 ± 0.0450	0.099 ± 0.0406
Plateau (a. u.) [c]	5.56 ± 0.025	6.58 ± 0.028	6.33 ± 0.028	6.65 ± 0.028	6.50 ± 0.031	6.30 ± 0.030	6.67 ± 0.030	6.65 ± 0.031	5.81 ± 0.028
K (min^{-1}) [d]	0.41 ± 0.015	0.38 ± 0.013	0.43 ± 0.016	0.50 ± 0.020	0.49 ± 0.021	0.46 ± 0.019	0.43 ± 0.017	0.46 ± 0.019	0.47 ± 0.020
Tau (1/*K*, min) [e]	2.42	2.66	2.34	1.98	2.05	2.17	2.34	2.17	2.13
Half-life (min) [f]	1.68	1.85	1.62	1.37	1.42	1.51	1.62	1.50	1.48
Span (a. u.) [g]	5.51 ± 0.043	6.53 ± 0.048	6.26 ± 0.049	6.52 ± 0.048	6.37 ± 0.054	6.20 ± 0.052	6.56 ± 0.052	6.55 ± 0.054	5.71 ± 0.049
R^2	0.9957	0.9961	0.9955	0.9961	0.9948	0.9948	0.9953	0.9951	0.9947

[a] T_0 is the time at which the ^{13}C-glc_{ex} begins. T_0 was set as a constant time point when the highest R^2 value was achieved.

[b] Y_0 is the average of relative peak amplitudes up to time T_0.

[c] *Plateau* is the relative peak amplitudes at infinite times.

[d] K is the rate constant.

[e] *Tau* is the time constant, which was computed as the reciprocal of *K*.

[f] *Half-life* was computed as *ln* (2)/*K*.

[g] *Span* is the difference between Y_0 and *Plateau*.

Values were expressed as mean ± SEM.

Table S6. Lactate Flux Profile Best Fit Parameters.

	Control_1	Control_2	Control_3	1×Cam_1	1×Cam_2	1×Cam_3	2×Cam_1	2×Cam_2	2×Cam_3
T_0 (min) [a]	= 22.72	= 22.72	= 25.07	= 21.15	= 21.15	= 21.15	= 21.15	= 21.15	= 21.15
Y_0 (mM/mM ATP) [b]	0.177 ± 0.0090	0.132 ± 0.0082	0.121 ± 0.0075	0.120 ± 0.0077	0.121 ± 0.0074	0.106 ± 0.0068	0.128 ± 0.0071	0.117 ± 0.0080	0.097 ± 0.0075
Plateau (mM/mM ATP) [c]	1.58 ± 0.013	1.48 ± 0.022	1.73 ± 0.038	1.16 ± 0.019	1.14 ± 0.016	0.98 ± 0.015	1.15 ± 0.018	1.19 ± 0.018	0.99 ± 0.015
K (min^{-1}) [d]	0.13 ± 0.006	0.12 ± 0.006	0.09 ± 0.005	0.11 ± 0.006	0.13 ± 0.006	0.12 ± 0.007	0.11 ± 0.006	0.12 ± 0.006	0.13 ± 0.007
Tau (1/K, min) [e]	7.94	8.33	11.12	8.70	7.80	8.10	8.76	8.14	7.43
Half-life (min) [f]	5.50	5.77	7.71	6.03	5.41	5.62	6.08	5.64	5.15
Span (mM/mM ATP) [g]	1.40 ± 0.023	1.35 ± 0.022	1.61 ± 0.037	1.04 ± 0.019	1.02 ± 0.017	0.88 ± 0.016	1.02 ± 0.018	1.07 ± 0.019	0.89 ± 0.016
R^2	0.9929	0.9934	0.9944	0.9908	0.9918	0.9904	0.9918	0.9910	0.9892
F_{in} (mM•min^{-1}•mM ATP^{-1}) [h]	0.199	0.178	0.155	0.133	0.146	0.121	0.131	0.146	0.133

[a] T_0 is the time at which the ^{13}C-labeled signal begins. T_0 was set as a constant time point when the highest R^2 value was achieved.

[b] Y_0 is the average of relative ^{13}C-labeled concentration up to time T_0.

[c] *Plateau* is relative ^{13}C-labeled concentration at infinite times.

[d] K is the rate constant.

[e] *Tau* is the time constant, which was computed as the reciprocal of K.

[f] *Half-life* was computed as *ln* (2)/K.

[g] *Span* is the difference between Y_0 and *Plateau*.

[h] F_{in} is the flux-in rate of ^{13}C-labeled signal, which was computed as the product of the mean *Plateau* and the mean K.

Values were expressed as mean ± SEM.

Table S7. Formate Flux Profile Best Fit Parameters.

	Control_1	Control_2	Control_3	1×Cam_1	1×Cam_2	1×Cam_3	2×Cam_1	2×Cam_2	2×Cam_3
T_0 (min) [a]	= 10.97	= 13.32	= 14.10	= 16.67	= 11.75	= 10.97	= 12.53	= 12.53	= 12.53
Y_0 (mM/mM ATP) [b]	0.222 ± 0.0017	0.017 ± 0.0021	0.108 ± 0.0017	-0.020 ± 0.0016	0.058 ± 0.0013	0.067 ± 0.0012	0.097 ± 0.0015	0.050 ± 0.0013	0.003 ± 0.0012
Plateau (mM/mM ATP) [c]	0.78 ± 0.031	0.96 ± 0.105	0.78 ± 0.066	0.35 ± 0.023	0.40 ± 0.022	0.39 ± 0.017	0.54 ± 0.029	0.48 ± 0.034	0.29 ± 0.016
K (min^{-1}) [d]	0.021 ± 0.0017	0.014 ± 0.0020	0.016 ± 0.0021	0.030 ± 0.0028	0.024 ± 0.0022	0.025 ± 0.0020	0.022 ± 0.0021	0.019 ± 0.0020	0.027 ± 0.0024
Tau (1/*K*, min) [e]	46.97	69.55	60.96	33.34	42.11	39.52	44.56	52.32	36.40
Half-life (min) [f]	32.56	48.21	42.25	23.11	29.19	27.40	30.88	36.27	25.23
Span (mM/mM ATP) [g]	0.56 ± 0.031	0.94 ± 0.104	0.67 ± 0.066	0.37 ± 0.023	0.34 ± 0.021	0.32 ± 0.016	0.44 ± 0.029	0.43 ± 0.034	0.29 ± 0.016
R^2	0.9959	0.9950	0.9950	0.9927	0.9936	0.9948	0.9944	0.9947	0.9933
F_{in} (mM•min^{-1}•mM ATP^{-1}) [h]	0.0166	0.0138	0.0127	0.0106	0.0094	0.0099	0.0120	0.0092	0.0080

[a] T_0 is the time at which the ^{13}C-labeled signal begins. T_0 was set as a constant time point when the highest R^2 value was achieved.

[b] Y_0 is the average of relative ^{13}C-labeled concentration up to time T_0.

[c] *Plateau* is relative ^{13}C-labeled concentration at infinite times.

[d] K is the rate constant.

[e] *Tau* is the time constant, which was computed as the reciprocal of K.

[f] *Half-life* was computed as *ln* (2)/K.

[g] *Span* is the difference between Y_0 and *Plateau*.

[h] F_{in} is the flux-in rate of ^{13}C-labeled signal, which was computed as the product of the mean *Plateau* and the mean K.

Values were expressed as mean ± SEM.

Table S8. Ethanol Flux Profile Best Fit Parameters.

	Control_1	Control_2	Control_3	1×Cam_1	1×Cam_2	1×Cam_3	2×Cam_1	2×Cam_2	2×Cam_3
T_0 (min) [a]	= 21.93	= 22.72	= 25.07	= 21.93	= 21.15	= 21.15	= 21.93	= 21.93	= 21.15
Y_0 (mM/mM ATP) [b]	0.078 ± 0.0042	0.051 ± 0.0034	0.051 ± 0.0031	0.062 ± 0.0026	0.054 ± 0.0024	0.045 ± 0.0036	0.052 ± 0.0028	0.043 ± 0.0026	0.031 ± 0.0020
Plateau (mM/mM ATP) [c]	0.85 ± 0.013	0.64 ± 0.009	0.74 ± 0.016	0.38 ± 0.006	0.37 ± 0.006	0.57 ± 0.010	0.39 ± 0.008	0.33 ± 0.006	0.27 ± 0.005
K (min^{-1}) [d]	0.10 ± 0.005	0.12 ± 0.005	0.09 ± 0.004	0.12 ± 0.007	0.11 ± 0.006	0.11 ± 0.006	0.12 ± 0.008	0.12 ± 0.008	0.12 ± 0.007
Tau (1/K, min) [e]	9.53	8.11	11.53	8.14	8.76	9.51	8.61	8.21	8.41
Half-life (min) [f]	6.61	5.62	7.99	5.64	6.07	6.59	5.97	5.69	5.83
Span (mM/mM ATP) [g]	0.77 ± 0.013	0.59 ± 0.009	0.69 ± 0.016	0.32 ± 0.006	0.32 ± 0.006	0.52 ± 0.010	0.34 ± 0.008	0.29 ± 0.006	0.23 ± 0.005
R^2	0.9945	0.9939	0.9947	0.9885	0.9910	0.9915	0.9877	0.9867	0.9897
F_{in} ($mM \bullet min^{-1} \bullet mM\ ATP^{-1}$) [h]	0.0892	0.0788	0.0641	0.0469	0.0428	0.0596	0.0452	0.0408	0.0315

[a] T_0 is the time at which the ^{13}C-labeled signal begins. T_0 was set as a constant time point when the highest R^2 value was achieved.

[b] Y_0 is the average of relative ^{13}C-labeled concentration up to time T_0.

[c] *Plateau* is relative ^{13}C-labeled concentration at infinite times.

[d] K is the rate constant.

[e] *Tau* is the time constant, which was computed as the reciprocal of K.

[f] *Half-life* was computed as *ln* (2)/K.

[g] *Span* is the difference between Y_0 and *Plateau*.

[h] F_{in} is the flux-in rate of ^{13}C-labeled signal, which was computed as the product of the mean *Plateau* and the mean K.

Values were expressed as mean ± SEM.

Table S9. Acetate Flux Profile Best Fit Parameters.

	Control_1	Control_2	Control_3	1×Cam_1	1×Cam_2	1×Cam_3	2×Cam_1	2×Cam_2	2×Cam_3
T_0 (min) [a]	= 21.15	= 21.15	= 21.93	= 21.93	= 21.93	= 22.72	= 21.93	= 22.72	= 21.15
Y_0 (mM/mM ATP) [b]	0.035 ± 0.0013	0.033 ± 0.0015	0.023 ± 0.0009	0.032 ± 0.0010	0.021 ± 0.0009	0.022 ± 0.0008	0.034 ± 0.0011	0.019 ± 0.0009	0.018 ± 0.0006
Plateau (mM/mM ATP) [c]	0.22 ± 0.004	0.31 ± 0.006	0.19 ± 0.003	0.15 ± 0.003	0.13 ± 0.002	0.13 ± 0.002	0.16 ± 0.004	0.10 ± 0.003	0.09 ± 0.002
K (min^{-1}) [d]	0.10 ± 0.006	0.08 ± 0.004	0.09 ± 0.005	0.10 ± 0.007	0.11 ± 0.007	0.11 ± 0.007	0.09 ± 0.007	0.10 ± 0.009	0.09 ± 0.007
Tau (1/*K*, min) [e]	10.22	12.16	10.75	9.67	8.70	9.38	10.55	9.63	11.60
Half-life (min) [f]	7.08	8.43	7.45	6.71	6.03	6.50	7.31	6.68	8.04
Span (mM/mM ATP) [g]	0.18 ± 0.004	0.27 ± 0.006	0.16 ± 0.003	0.12 ± 0.003	0.11 ± 0.002	0.10 ± 0.002	0.12 ± 0.004	0.09 ± 0.003	0.07 ± 0.002
R^2	0.9904	0.9940	0.9936	0.9875	0.9870	0.9888	0.9847	0.9798	0.9855
F_{in} (mM•min^{-1}•mM ATP^{-1}) [h]	0.0212	0.0252	0.0174	0.0156	0.0148	0.0133	0.0149	0.0109	0.008

[a] T_0 is the time at which the ^{13}C-labeled signal begins. T_0 was set as a constant time point when the highest R^2 value was achieved.

[b] Y_0 is the average of relative ^{13}C-labeled concentration up to time T_0.

[c] *Plateau* is relative ^{13}C-labeled concentration at infinite times.

[d] *K* is the rate constant.

[e] *Tau* is the time constant, which was computed as the reciprocal of *K*.

[f] *Half-life* was computed as *ln* (2)/*K*.

[g] *Span* is the difference between Y_0 and *Plateau*.

[h] F_{in} is the flux-in rate of ^{13}C-labeled signal, which was computed as the product of the mean *Plateau* and the mean *K*.

Values were expressed as mean ± SEM.

Table S10. Alanine Flux Profile Best Fit Parameters.

	Control_1	Control_2	Control_3	1×Cam_1	1×Cam_2	1×Cam_3	2×Cam_1	2×Cam_2	2×Cam_3
T_0 (min) [a]	= 21.15	= 21.15	= 21.93	= 22.72	= 20.37	= 21.15	= 21.15	= 20.37	= 20.37
Y_0 (mM/mM ATP) [b]	0.022 ± 0.0005	0.024 ± 0.0005	0.018 ± 0.0004	0.018 ± 0.0004	0.014 ± 0.0004	0.014 ± 0.0004	0.016 ± 0.0005	0.011 ± 0.0004	0.011 ± 0.0004
Plateau (mM/mM ATP) [c]	0.91 ± 0.201	0.87 ± 0.189	0.88 ± 0.150	0.53 ± 0.038	0.44 ± 0.031	0.38 ± 0.025	0.85 ± 0.143	0.53 ± 0.053	0.36 ± 0.016
K (min^{-1}) [d]	0.007 ± 0.0018	0.007 ± 0.0017	0.007 ± 0.0014	0.017 ± 0.0002	0.016 ± 0.0014	0.018 ± 0.0015	0.009 ± 0.0016	0.013 ± 0.0015	0.021 ± 0.0012
Tau (1/K, min) [e]	135.74	140.26	138.27	59.21	62.78	55.29	115.55	78.63	46.90
Half-life (min) [f]	94.09	97.22	95.84	41.04	43.52	38.32	80.09	54.51	32.51
Span (mM/mM ATP) [g]	0.89 ± 0.201	0.84 ± 0.189	0.87 ± 0.150	0.51 ± 0.038	0.42 ± 0.031	0.37 ± 0.025	0.84 ± 0.143	0.52 ± 0.053	0.35 ± 0.016
R^2	0.9976	0.9978	0.9987	0.9986	0.9986	0.9985	0.9981	0.9983	0.9989
F_{in} (mM•min^{-1}•mM ATP^{-1}) [h]	0.0067	0.0062	0.0064	0.0090	0.0070	0.0069	0.0074	0.0068	0.0076

[a] T_0 is the time at which the ^{13}C-labeled signal begins. T_0 was set as a constant time point when the highest R^2 value was achieved.

[b] Y_0 is the average of relative ^{13}C-labeled concentration up to time T_0.

[c] *Plateau* is relative ^{13}C-labeled concentration at infinite times.

[d] K is the rate constant.

[e] *Tau* is the time constant, which was computed as the reciprocal of K.

[f] *Half-life* was computed as ln (2)/K.

[g] *Span* is the difference between Y_0 and *Plateau*.

[h] F_{in} is the flux-in rate of ^{13}C-labeled signal, which was computed as the product of the mean *Plateau* and the mean K.

Values were expressed as mean ± SEM.

Table S11. Valine Flux Profile Best Fit Parameters.

	Control_1	Control_2	Control_3	1×Cam_1	1×Cam_2	1×Cam_3	2×Cam_1	2×Cam_2	2×Cam_3
T_0 (min) [a]	= 25.85	= 28.20	= 26.63	= 27.42	= 28.20	= 27.42	= 25.85	= 29.77	= 26.63
Y_0 (mM/mM ATP) [b]	0.009 ± 0.0004	0.004 ± 0.0004	0.006 ± 0.0003	0.005 ± 0.0003	0.006 ± 0.0003	0.004 ± 0.0003	0.005 ± 0.0003	0.004 ± 0.0003	0.008 ± 0.0002
Plateau (mM/mM ATP) [c]	0.13 ± 0.040	0.08 ± 0.025	0.12 ± 0.047	0.08 ± 0.024	0.07 ± 0.019	0.08 ± 0.027	0.12 ± 0.042	0.08 ± 0.025	0.06 ± 0.021
K (min^{-1}) [d]	0.019 ± 0.0077	0.027 ± 0.0105	0.016 ± 0.0080	0.021 ± 0.0086	0.028 ± 0.0114	0.022 ± 0.0106	0.016 ± 0.0068	0.025 ± 0.0109	0.021 ± 0.0096
Tau (1/*K*, min) [e]	52.30	37.48	61.40	46.63	35.46	45.72	64.05	39.47	48.52
Half-life (min) [f]	36.26	25.98	42.56	32.32	24.58	31.69	44.39	27.36	33.63
Span (mM/mM ATP) [g]	0.12 ± 0.040	0.08 ± 0.025	0.11 ± 0.047	0.07 ± 0.024	0.06 ± 0.019	0.07 ± 0.027	0.11 ± 0.042	0.07 ± 0.025	0.05 ± 0.021
R^2	0.9745	0.9655	0.9746	0.9743	0.9602	0.9605	0.9801	0.9699	0.9646
F_{in} ($mM \bullet min^{-1} \bullet mM\ ATP^{-1}$) [h]	0.0024	0.0022	0.0019	0.0017	0.0019	0.0016	0.0018	0.0019	0.0013

[a] T_0 is the time at which the ^{13}C-labeled signal begins. T_0 was set as a constant time point when the highest R^2 value was achieved.

[b] Y_0 is the average of relative ^{13}C-labeled concentration up to time T_0.

[c] *Plateau* is relative ^{13}C-labeled concentration at infinite times.

[d] *K* is the rate constant.

[e] *Tau* is the time constant, which was computed as the reciprocal of *K*.

[f] *Half-life* was computed as *ln* (2)/*K*.

[g] *Span* is the difference between Y_0 and *Plateau*.

[h] F_{in} is the flux-in rate of ^{13}C-labeled signal, which was computed as the product of the mean *Plateau* and the mean *K*.

Values were expressed as mean ± SEM.

Table S12. Glu-γ Flux Profile Best Fit Parameters.

	Control_1	Control_2	Control_3	1×Cam_1	1×Cam_2	1×Cam_3	2×Cam_1	2×Cam_2	2×Cam_3
T_0 (min) [a]	= 18.80	= 20.37	= 21.15	= 20.37	= 21.15	= 20.37	= 20.37	= 21.93	= 19.58
Y_0 (mM/mM ATP) [b]	-0.0010 ± 0.0013	-0.0005 ± 0.0012	-0.0065 ± 0.0011	-0.0060 ± 0.0010	0.0056 ± 0.0008	-0.0080 ± 0.0010	-0.0180 ± 0.0010	-0.0099 ± 0.0010	0.0016 ± 0.0007
Plateau (mM/mM ATP) [c]	2.00 ± 0.527	0.94 ± 0.130	0.32 ± 0.017	0.31 ± 0.026	0.23 ± 0.009	0.37 ± 0.005	0.36 ± 0.005	0.26 ± 0.024	0.12 ± 0.007
K (min^{-1}) [d]	0.006 ± 0.0019	0.013 ± 0.0021	0.036 ± 0.0029	0.027 ± 0.0031	0.043 ± 0.0030	0.020 ± 0.0032	0.021 ± 0.0033	0.032 ± 0.0041	0.042 ± 0.0041
Tau (1/K, min) [e]	154.48	75.98	27.99	36.73	23.17	50.29	48.21	31.01	23.80
Half-life (min) [f]	107.08	52.67	19.40	25.46	16.06	34.86	33.42	21.49	16.49
Span (mM/mM ATP) [g]	2.00 ± 0.526	0.94 ± 0.130	0.33 ± 0.017	0.31 ± 0.026	0.22 ± 0.009	0.38 ± 0.048	0.37 ± 0.046	0.27 ± 0.024	0.12 ± 0.007
R^2	0.9970	0.9967	0.9946	0.9935	0.9951	0.9928	0.9924	0.9907	0.9895
F_{in} (mM•min^{-1}•mM ATP^{-1}) [h]	0.0130	0.0124	0.0115	0.0084	0.0098	0.0073	0.0074	0.0085	0.0052

[a] T_0 is the time at which the ^{13}C-labeled signal begins. T_0 was set as a constant time point when the highest R^2 value was achieved.

[b] Y_0 is the average of relative ^{13}C-labeled concentration up to time T_0.

[c] *Plateau* is relative ^{13}C-labeled concentration at infinite times.

[d] K is the rate constant.

[e] *Tau* is the time constant, which was computed as the reciprocal of K.

[f] *Half-life* was computed as ln (2)/K.

[g] *Span* is the difference between Y_0 and *Plateau*.

[h] F_{in} is the flux-in rate of ^{13}C-labeled signal, which was computed as the product of the mean *Plateau* and the mean K.

Values were expressed as mean ± SEM.

Table S13. Succinate Flux Profile Best Fit Parameters.

	Control_1	Control_2	Control_3	1×Cam_1	1×Cam_2	1×Cam_3	2×Cam_1	2×Cam_2	2×Cam_3
T_0 (min) [a]	= 23.50	= 23.50	= 25.07	= 22.72	= 22.72	= 22.72	= 24.28	= 22.72	= 21.15
Y_0 (mM/mM ATP) [b]	0.022 ± 0.0006	0.021 ± 0.0006	0.013 ± 0.0004	0.016 ± 0.0003	0.012 ± 0.0003	0.011 ± 0.0003	0.016 ± 0.0004	0.010 ± 0.0003	0.009 ± 0.0002
Plateau (mM/mM ATP) [c]	0.99 ± 0.216	2.09 ± 0.802	1.18 ± 0.317	1.87 ± 0.653	1.02 ± 0.231	0.87 ± 0.127	0.79 ± 0.226	0.73 ± 0.157	1.13 ± 0.369
K (min^{-1}) [d]	0.009 ± 0.0021	0.004 ± 0.0017	0.007 ± 0.0019	0.003 ± 0.0012	0.006 ± 0.0014	0.008 ± 0.0012	0.007 ± 0.0022	0.007 ± 0.0015	0.003 ± 0.0012
Tau (1/*K*, min) [e]	114.65	234.19	151.64	305.01	178.11	131.99	142.86	151.80	288.32
Half-life (min) [f]	79.47	162.33	105.11	211.42	123.45	91.49	99.03	105.22	199.84
Span (mM/mM ATP) [g]	0.97 ± 0.216	2.07 ± 0.802	1.17 ± 0.317	1.85 ± 0.653	1.00 ± 0.231	0.86 ± 0.136	0.77 ± 0.226	0.72 ± 0.157	1.13 ± 0.370
R^2	0.9973	0.9982	0.9981	0.9991	0.9988	0.9989	0.9973	0.9985	0.9990
F_{in} ($mM{\bullet}min^{-1}{\bullet}mM\ ATP^{-1}$) [h]	0.0086	0.0089	0.0078	0.0061	0.0057	0.0066	0.0055	0.0048	0.0039

[a] T_0 is the time at which the ^{13}C-labeled signal begins. T_0 was set as a constant time point when the highest R^2 value was achieved.

[b] Y_0 is the average of relative ^{13}C-labeled concentration up to time T_0.

[c] *Plateau* is relative ^{13}C-labeled concentration at infinite times.

[d] K is the rate constant.

[e] *Tau* is the time constant, which was computed as the reciprocal of K.

[f] *Half-life* was computed as *ln* (2)/*K*.

[g] *Span* is the difference between Y_0 and *Plateau*.

[h] F_{in} is the flux-in rate of ^{13}C-labeled signal, which was computed as the product of the mean *Plateau* and the mean K.

Values were expressed as mean ± SEM.

References.

(1) Loveland, A. B.; Korostelev, A. A., Structural dynamics of protein S1 on the 70S ribosome visualized by ensemble cryo-EM. *Methods* **2018,** *137*, 55-66.

(2) D. Blangy, H. B.; Monod, J. Kinetics of the Allosteric Interactions of Phosphofructokinase from *Escherichia coli*. *J Mol Biol* **1968**, 31, 13-35.

(3) Byrnes, M.; Zhu, X.; Younathan, E. S.; Chang, S. H. Kinetic Characteristics of Phosphofructokinase from *Bacillus stearothermophilus*: MgATP Nonallosterically Inhibits the Enzyme. *Biochemistry* **1994**, 33 (11), 3424-3431.

(4) Tarmy, E. M.; Kaplan, N. O. Kinetics of Escherichia coli B D-Lactate Dehydrogenase and Evidence for Pyruvate-controlled Change in Conformation. *Journal of Biological Chemistry* **1968**, 243 (10), 2587-2596. DOI: 10.1016/s0021-9258(18)93414-9.

(5) Levan, K. M.; Goldberg, E. Properties of human testis-specific lactate dehydrogenase expressed from Escherichia coli. *Biochem J* **1991**, 273, 587-592.

(6) Commichau, F. M.; Rothe, F. M.; Herzberg, C.; Wagner, E.; Hellwig, D.; Lehnik-Habrink, M.; Hammer, E.; Volker, U.; Stulke, J. Novel activities of glycolytic enzymes in Bacillus subtilis: interactions with essential proteins involved in mRNA processing. *Mol Cell Proteomics* **2009**, 8 (7), 1350-1360. DOI: 10.1074/mcp.M800546-MCP200.

(7) Chowdhury, S.; Hepper, S.; Lodi, M. K.; Saier, M. H., Jr.; Uetz, P. The Protein Interactome of Glycolysis in Escherichia coli. *Proteomes* **2021**, 9 (2), 16. DOI: 10.3390/proteomes9020016.

(8) Yu, J.; Ramirez, L. M.; Premo, A.; Busch, D. B.; Lin, Q.; Burz, D. S.; Shekhtman, A. Ribosome-Amplified Metabolism, RAMBO, Measured by NMR Spectroscopy. *Biochemistry* **2021**, 60 (24), 1885-1895. DOI: 10.1021/acs.biochem.1c00074.

(9) Yu, J.; Ramirez, L. M.; Lin, Q.; Burz, D. S.; Shekhtman, A. Ribosome External Electric Field Regulates Metabolic Enzyme Activity: The RAMBO Effect. *J Phys Chem B* **2024**, 128 (29), 7002-7021. DOI: 10.1021/acs.jpcb.4c00628.
